# Metal–Organic Framework-Based Chemiresistive Array Paired with Machine Learning Algorithms for the Detection and Differentiation of Toxic Gases

**DOI:** 10.1021/acssensors.5c02182

**Published:** 2025-10-06

**Authors:** Georganna Benedetto, Patrick Damacet, Elissa O. Shehayeb, Gbenga Fabusola, Cory M. Simon, Katherine A. Mirica

**Affiliations:** † 3728 Dartmouth College, Department of Chemistry, Hanover, New Hampshire 03755, United States; ‡ 508120 Oregon State University, School of Chemical, Biological, and Environmental Engineering, Corvallis, Oregon 97331, United States

**Keywords:** chemiresistive sensors, metal—organic frameworks, gaseous analyte differentiation, sensor selectivity, machine learning

## Abstract

The development of low-power, sensitive, and selective gas sensors capable of detecting and differentiating toxic gases is pivotal for safety and environmental monitoring. This paper describes a chemiresistive sensor array comprising a series of three conductive hexahydroxytriphenylene-based metal–organic frameworks (MOFs) (M_3_(HHTP)_2_ (M = Ni, Cu, Zn)) capable of detecting and differentiating parts-per-million (ppm) levels of carbon monoxide (CO), ammonia (NH_3_), sulfur dioxide (SO_2_), hydrogen sulfide (H_2_S), and nitric oxide (NO), as well as binary mixtures of SO_2_ and H_2_S in dry nitrogen at room temperature. This capability arises from variations in the identity of the linking metal and the framework packing pattern across the materials in the array. To visualize the response pattern of the sensor array and map it to a predicted gas composition, principal component analysis and random forest classification are employed. Both machine learning techniques confirm the ability to discriminate CO, NH_3_, SO_2_, H_2_S, and NO analytes as well as binary SO_2_/H_2_S mixtures at ppm concentrations using the response of the array. Moreover, a feature importance method applied to the classifier assigns importance scores to each sensor in the array to quantify the impact of individual materials on analyte discrimination. Spectroscopic investigations provide insight into how the structural features of the MOFs influence sensing performance and ascertain material–analyte interactions governing sensing selectivity for SO_2_/H_2_S binary mixtures.

## Graphical abstract

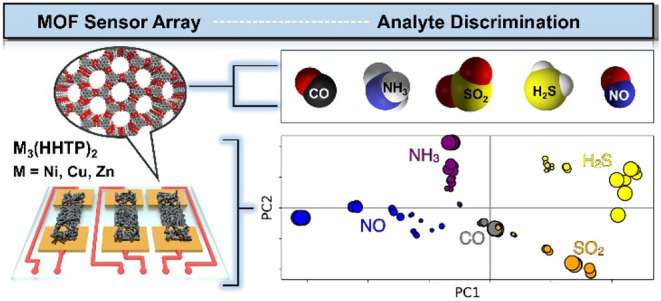

The widespread presence of toxic gases due to geological and anthropogenic activities can result in both acute and chronic exposure.^1^ The risk of exposure necessitates low-powered, portable, and reliable gas-sensing technology to monitor toxic gases and ensure equitable access to clean air.^2,3^ Various commercial wearable gas monitors enable simultaneous detection of up to six hazardous gases, including carbon monoxide (CO), ammonia (NH_3_), sulfur dioxide (SO_2_), hydrogen sulfide (H_2_S), and nitrogen dioxide (NO_2_).^4–6^ However, commercial multianalyte gas monitors feature a single electrochemical sensor dedicated to each analyte, resulting in the need for an increasing number of sensor nodes, larger size, and greater power requirements. Additionally, these sensors exhibit cross-sensitivity with interfering gases, necessitating the use of selectively permeable membranes or adsorbent layers to prevent cross-reactive gases from reaching the sensing material.^7,8^ When these barriers degrade or become damaged, the ingress of cross-reactive gases can produce false positives or false negatives.^9^ False positives cause costly, unnecessary disruptions and a loss of trust in sensors, while false negatives may lead to exposure to harmful concentrations of toxic gases.^10^ As such, it is imperative to (i) develop materials capable of multianalyte detection in complex mixtures and (ii) utilize machine learning to deconvolute cross-reactivity in an array of sensing materials.^11,12^

Sensors are particularly prone to false readings when multiple analytes are simultaneously present, as the sensor materials can cross-react with different species in a mixture, thereby obfuscating the readout. For instance, some commercial SO_2_ sensors have a 5% interference due to H_2_S, and some NO sensors have a 25% interference due to CO at select analyte concentrations.^13^ These gas mixtures are important to distinguish. First, SO_2_ and H_2_S may both be present in the parts-per-million (ppm) range^14–16^ in pulp and paper mills,^17,18^ biogas plants,^19^ as well as nearby volcanoes and hydrothermal systems.^20,21^ According to Occupational Safety and Health Administration (OSHA), the permissible exposure limits (PELs) of SO_2_ and H_2_S are 5 and 20 ppm,^22,23^ respectively, necessitating differentiation of these gases in the low to mid-ppm range, where human health is at risk and the margin for sensor error is small. Second, CO and NO are both present as air pollutants due to vehicle emissions, with PELs of 50 and 25 ppm, respectively.^24^ Binary mixtures pose unique health threats and require targeted actions depending on the concentration and ratio of gases present.^25,26^ Simultaneous detection and differentiation of gaseous mixtures like SO_2_ and H_2_S is crucial for assessing health risks, avoiding false positive and false negative alarms, diagnosing problems, and identifying the source of these gases in industrial settings. As such, monitoring and discriminating these gaseous mixtures in locations where they coexist is important for worker safety and environmental monitoring.

An effective sensing platform for monitoring gas mixtures is chemiresistive materials, which exhibit a change in electrical resistance in response to a changing chemical environment due to material–analyte interactions.^11,27^ Though chemiresistive materials such as conductive polymers^28^ and carbon nanotubes^29^ have successfully detected small molecule gases, conductive framework materials, namely metal–organic frameworks (MOFs), are particularly advantageous. MOFs offer tunable surface chemistry and charge transport enabled by reticular synthesis and rational linkage design. Conductive framework materials have successfully detected and differentiated single analyte exposures through chemiresistive means but, to the best of our knowledge, have never been used to detect binary mixtures.^30–51^ Metal oxide (MO) nanowire arrays have been used to discriminate binary mixtures of H_2_S and NO_2_,^52^ as well as stepwise exposures of CO_2_, NH_3_, H_2_S, and NO_2_.^53^ However, MOs typically require high operating temperatures and driving voltages, limiting their widespread application.^54^ There remains a need for sensors for toxic gases capable of functioning in complex gas mixtures with low limits of detection (LODs) at room temperature and with low power requirements.

This work describes an array of hexahydroxytriphenylene (HHTP)-based MOFs (M_3_(HHTP)_2_ (M = Ni, Cu, Zn)) as chemiresistive sensors, which, when coupled with machine learning algorithms to parse their response patterns, successfully differentiate CO, NH_3_, SO_2_, H_2_S, and NO, as well as binary mixtures of SO_2_ and H_2_S, in dry nitrogen at room temperature. We chose to focus on this class of MOFs due to their straightforward synthetic accessibility, exceptional stability afforded by the optimal d−π linkages between the organic linker and metal node, and abundant active sites for analytes. Utilization of a series of 2D MOFs featuring hexagonal pores as sensing materials enables the alteration of the metal node linking organic ligands, both directly modulating analyte-MOF interactions and resulting in variations in the packing pattern of the 2D sheets that influence the pore aperture and hydration. These structural differences lead to variations in the material–analyte interactions and endow the array with a response pattern that provides rich information about the gas composition. In turn, we leverage machine learning algorithms, including principal component analysis (PCA) and random forest classifiers, to parse the response pattern of the sensor array and assess its ability to detect and discriminate the analytes and mixtures. PCA allows for the visualization of the high-dimensional response pattern of the sensor array and the qualitative assessment of how well the responses are clustered according to the analyte that produced them. The random forest algorithm learns from examples to map the response pattern of the sensor array to a categorical prediction of the gas composition. Furthermore, through a feature importance method, analysis of the random forest enables the assignment of importance scores to each material in the array. The hardware (an array of conductive MOFs integrated into devices) and software (random forest trained on labeled examples of responses) together constitute an “electronic nose”.^11^ By relying on *in situ* diffuse reflectance infrared Fourier transform spectroscopy (DRIFTS) and *ex situ* X-ray photoelectron spectroscopy (XPS), we elucidate modes of interaction between the MOFs and analytes in binary mixtures of SO_2_ and H_2_S. Using these spectroscopic techniques, we demonstrate how variable MOF packing patterns and redox lability modulate analyte cross-reactivity and, therefore, the ability of the array to discriminate sulfur-containing species. The sensor array presented herein exhibits sensitive, wide-range analyte differentiation and is the first effective conductive MOF-based discriminator for binary mixtures with variable analyte selectivity profiles.

## Results and Discussion

### Material Characterization

First, the M_3_(HHTP)_2_ (M = Ni, Cu, Zn) materials were prepared via hydrothermal synthesis as previously established in the literature.^55–57^ Commercially sourced HHTP was mixed with nickel­(II) acetate tetrahydrate (Ni­(OAc)_2_·4H_2_O), copper­(II) nitrate tetrahydrate Cu­(NO_3_)_2_ **·**4H_2_O), or zinc­(II) nitrate hexahydrate (Zn­(NO_3_)_2_ **·**6H_2_O) to form three MOFs, respectively.

While no base additive was used for the formation of Ni_3_(HHTP)_2_, a solution of 35% concentrated ammonia and 0.25 M sodium acetate were used to promote Cu- and Zn_3_(HHTP)_2_ formation, respectively. See Section SII for complete synthetic information. Prior to all characterization and sensing experiments, the MOF material was rigorously activated by solvent exchange with ethanol for 2 days, with fresh solvent added every 12 h. Finally, the MOF powder was dried for 48 h in a vacuum oven at 75 °C.

The MOF materials exhibited high degrees of crystallinity, as evidenced via powder X-ray diffraction (pXRD), with spectra well-approximated by the simulated crystal packing patterns (Figures S1–S3). While the Ni-based MOF exhibited intercalated packing, the Cu- and Zn_3_(HHTP)_2_ materials aligned with an AA slipped parallel structure (Figure 1a). As assessed through scanning electron microscopy (SEM), Ni- and Zn_3_(HHTP)_2_ crystals exhibited nanorod morphology on the scale of microns, while Cu_3_(HHTP)_2_ crystals appeared as irregular flakes (Figures 1b–d and S4–S6). All three materials exhibited the expected chemical composition, lattice parameters, thermal stability, conductivity, and porosity as confirmed via energy-dispersive spectroscopy (EDS), transmission electron microscopy (TEM), thermogravimetric analysis (TGA), four-point probe analysis, and Brunauer–Emmett–Teller (BET) analysis, respectively. For full details, see Sections SV, SVI, SVII, SIX, and SX, respectively. By leveraging the unique features afforded by these HHTP-based 2D frameworks—adjustable metal center identity and redox lability, as well as variation in packing pattern and hydration—a suite of chemiresistive materials is accessible through facile synthesis.

**Figure 1:**
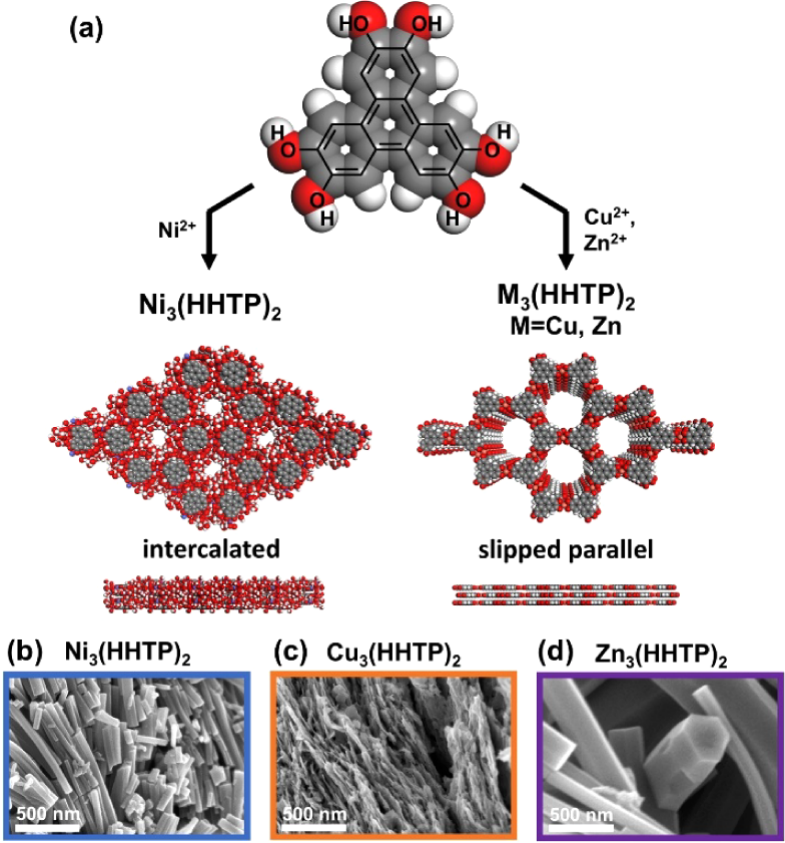
(a) Schematic of M_3_(HHTP)_2_ (M = Ni, Cu, Zn) synthesis accompanied by the simulated packing pattern of the MOF and (b–d) electron micrographs of the MOF morphology and crystallite size.

### Chemiresistive Sensing

We utilized M_3_(HHTP)_2_ (M = Ni, Cu, Zn) MOFs as chemiresistive materials for the detection and discrimination of reducing and oxidizing gases (CO, NH_3_, SO_2_, H_2_S, and NO) in dry nitrogen (N_2_) at room temperature. Devices were fabricated by sonicating each MOF into an aqueous suspension (concentration: 1.0 mg of MOF per 1.0 mL of H_2_O). The MOF suspension (10–15 μL) was dropcasted onto gold interdigitated electrodes (IDEs) with 10 μm interdigitation. Devices were then dried in an oven set to 85 °C for 10–15 min (see Section SXI for more details). The electrical resistances of typical devices ranged from 10 kΩ to 1.0 MΩ. Device response was reported as the negative normalized change in conductance (−Δ*G*/*G*_0_) over time (see Section SXI for experimental conditions). −Δ*G*/*G*_0_ is directly proportional to the change in resistance.

Devices were then exposed to varying concentrations (0.5–80 ppm) of analytes in N_2_ carrier gas (exposure time: 30 min), resulting in variable degrees, rates, and directions of response, as seen in Figures 2 and S18. All three MOF devices increased in conductance upon exposure to NO, an oxidizing gas. All three MOFs decreased in conductance upon exposure to CO, SO_2_, and H_2_S, which are reducing gases. These responses are consistent with the behavior of a p-type semiconductor.^37,58^ While some previous literature presented HHTP-based MOFs as n-type semiconductors, it is possible for the materials to switch types based on varying factors.^59,60^ The differences in directional response (i.e., an increase vs decrease in conductance) to NH_3_ suggest that the MOFs interact with this analyte in fundamentally distinct ways depending on the identity of the linking metal (see Section SXIV for further analysis). Over the course of analyte exposure, Ni-, Cu-, and Zn_3_(HHTP)_2_ devices exhibited the strongest response to SO_2_, H_2_S, and NO, respectively. Following CO exposure, all of the MOF devices exhibited reversibility. Conversely, following exposure to either H_2_S or NO, both Ni- and Cu_3_(HHTP)_2_ devices exhibited irreversible responses. All other analyte-MOF device pairings demonstrated semireversible responses (i.e., partial recovery of response) during the 17 min following cessation of analyte exposure.

**Figure 2:**
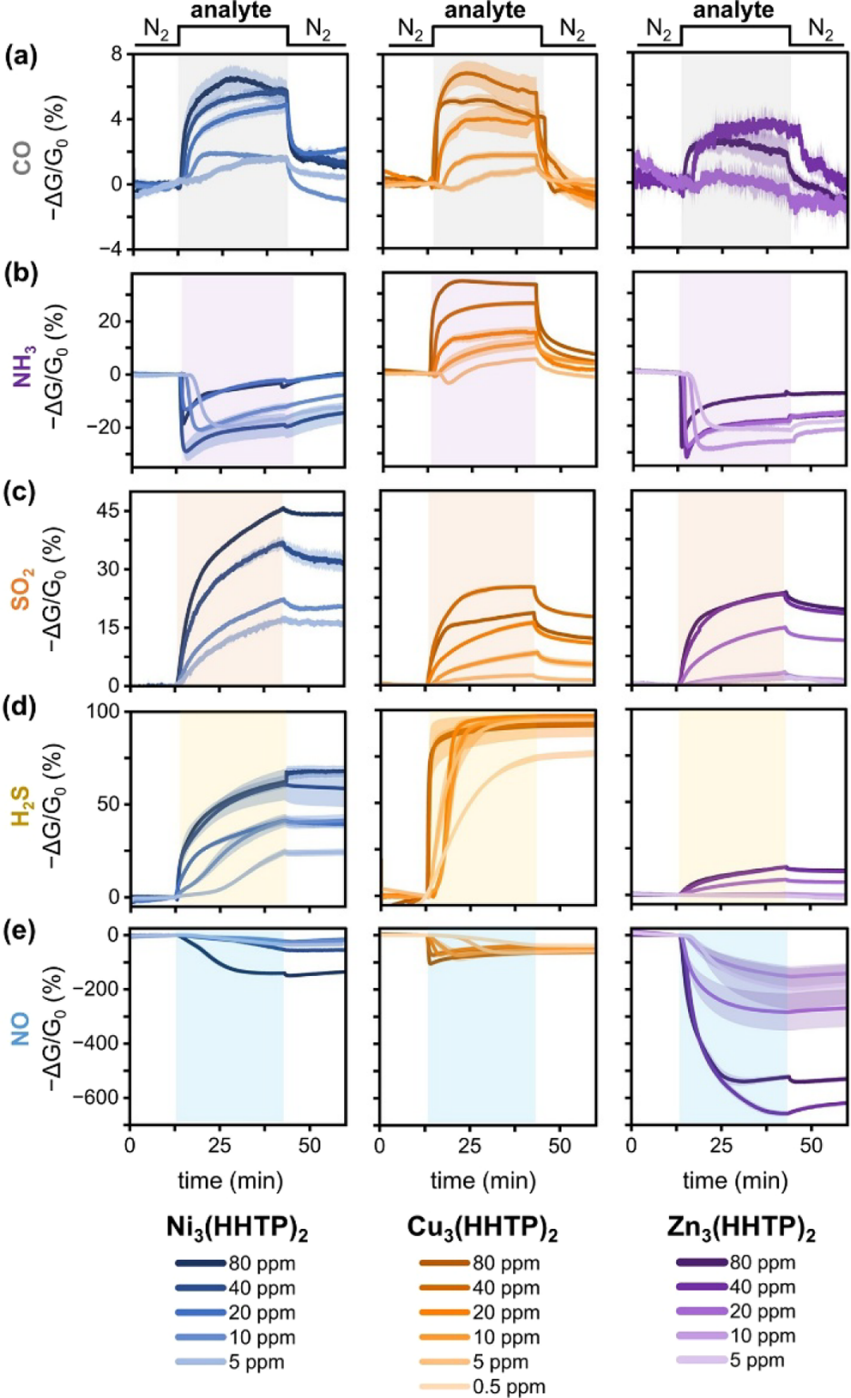
Sensing traces of M_3_(HHTP)_2_ (M = Ni, Cu, Zn) devices exposed to various concentrations of (a) CO, (b) NH_3_, (c) SO_2_, (d) H_2_S, and (e) NO in dry N_2_ carrier gas at room temperature over a 30 min exposure window. The analyte concentrations range from 0.5 to 80 ppm, 5 to 80 ppm, or 10 to 80 ppm depending on the device’s ability to respond to concentrations >20 ppm. Each curve represents the average sensing trace over a batch of devices exposed to an analyte at a specific concentration. The shaded area above and below the traces shows the standard deviation; the shaded rectangle indicates the analyte exposure time window.

The responses of the devices exhibited a clear dependence on the concentration of the analyte, as evidenced by the linear relationship between response features (maximum response at 1 min of analyte exposure and initial rate of response (RoR)) and concentration (Figure S19). In some instances, the linearity was not maintained at 80 ppm due to device saturation. In these cases, response values at 80 ppm were excluded. Subsequent exposures (2–4) of devices to an 80 ppm concentration of analytes demonstrated diminished responses compared to the initial exposure (Figures S20–S24 and Tables S2–S6). The reusability of each device was dependent on the reversibility of the response postexposure. Reproducibility tests showed that different synthetic batches of MOFs exhibited similar metrics across the first to fourth analyte exposures, as shown in Figures S30 and S31.

### Machine Learning to Parse the Response Pattern of the Gas Sensor Array

Here, we employed machine learning algorithms to parse the response pattern of the [Ni_3_(HHTP)_2_, Cu_3_(HHTP)_2_, Zn_3_(HHTP)_2_] sensor array and assess its ability to detect and discriminate both (i) pure analytes (CO, NH_3_, SO_2_, H_2_S, NO) and various concentrations thereof and (ii) binary mixtures of SO_2_ and H_2_S—both in dry N_2_ at room temperature. Our Python code and raw data are available at github.com/Fabuloski/gas_concentration_prediction. We first constructed a feature vector that characterized the response of the sensor array to exposure to a gas [mixture]. From the response curve of each sensor in the array, we extracted three features: the [all, signed] (1) maximum, (2) initial slope, and (3) area under the curve. Then, we concatenated these three response features from each of the three sensors in the array to obtain a sensor array response vector *x* ∈ $R^{9}$ . Each *x* is paired with a “label” *c* describing the analyte(s) and its concentration(s) that caused the observed response. We used machine learning algorithms to both visualize the response vectors and construct a map from the response vector to a predicted analyte/concentration.

### Principal Component Analysis (PCA) to Visually Assess Differentiation of Analytes

To evaluate the ability to differentiate single analytes in dry N_2_ at various concentrations (5, 10, 20, 40, 80 ppm) using the response of the [Ni_3_(HHTP)_2_, Cu_3_(HHTP)_2_, Zn_3_(HHTP)_2_] sensor array, we conducted principal component analysis (PCA) to visualize its response vectors (extracted from individual sensing traces averaged in Figure 2).^61,62^ Figure 3a displays the data matrix for PCA as a heatmap, with columns containing the sensor array response vectors under different analyte exposures, as indicated by the scatter plot below it (each feature was Yeo–Johnson transformed and then *z*-score normalized to achieve a standard normal-like distribution). We identified patterns in the response features indicative of the analyte and its concentration (e.g., responses to H_2_S and NO are opposite in sign and tend to increase in magnitude with concentration). Using PCA, we aimed to embed the 9D response vectors, located in the columns of the data matrix, into a 2D space for visualization while faithfully preserving the structure of the original vectors. Notably, PCA (unsupervised learning) does not use the analyte/concentration labels. Figure 3b displays this PCA embedding. We observed that the responses are well-clustered and separated according to the analyte. Along PC1, the clusters of responses to H_2_S (right) and NO (left) are significantly separated, reflecting the opposite direction of the responses to these two analytes observed in Figure 3a. Along PC2, the clusters of NH_3_ and SO_2_ responses are also clearly separated. The responses to CO lie near the origin, reflecting the minimal response to CO. Overall, all five response clusters (grouped by analyte) are well-separated, except for the response to 5 ppm of SO_2_, which lies within the CO cluster. Moreover, as the concentration of each analyte increases, the responses tend to grow in magnitude, i.e., move farther from the origin. In conclusion, the response vector of the [Ni_3_(HHTP)_2_, Cu_3_(HHTP)_2_, and Zn_3_(HHTP)_2_] sensor array contains sufficient information to detect and discriminate CO, NH_3_, SO_2_, H_2_S, and NO analytes in dry N_2_ at 5–80 ppm concentrations, as well as indicate the concentration of NH_3_, SO_2_, H_2_S, and NO.

**Figure 3:**
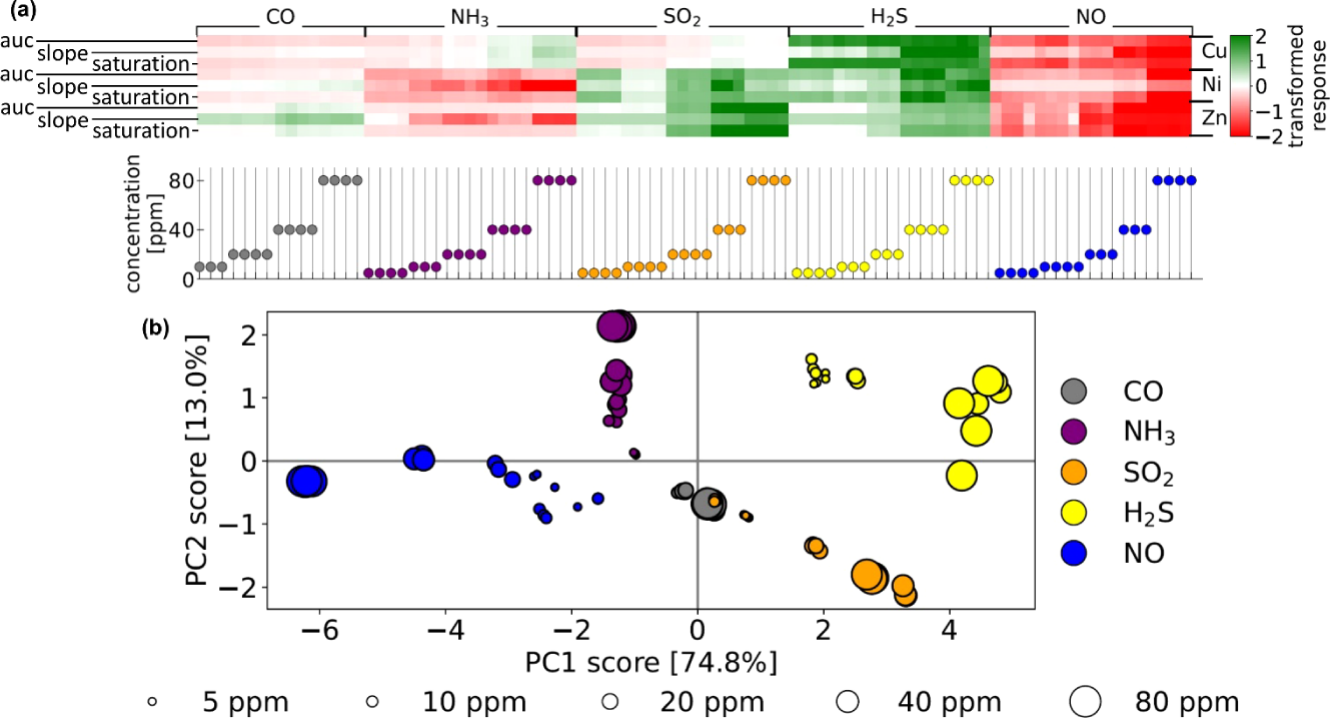
PCA (unsupervised learning) to evaluate the ability of the M_3_(HHTP)_2_ (M = Ni, Cu, Zn) sensor array to differentiate between various analytes at various concentrations in dry N_2_. (a) A heatmap of the data matrix containing, in its columns, the response vectors of the sensor array under different exposures, as indicated by the scatter plot below. (b) The 2D latent space containing the embeddings of the sensor array response vectors, colored and sized according to the analyte and its concentration, respectively, that produced it.

### Exposure to Binary Mixtures of Analytes

Inspired by the success in differentiating single analytes, we endeavored to explore exposing the sensor array to binary mixtures of analytes to (i) test the selectivity profile of the individual materials and (ii) probe the ability of the array to distinguish mixtures.

With exposure to binary mixtures of SO_2_ and H_2_S, the MOF devices exhibited a nuanced selectivity relationship. As seen in Figure 4, when exposed to both 40 ppm of SO_2_ and 40 ppm of H_2_S simultaneously, both Ni_3_(HHTP)_2_ and Cu_3_(HHTP)_2_ showed sensing traces resembling single H_2_S exposure, likely due to the preferential binding of H_2_S to the frameworks. However, upon exposure to 40 ppm of SO_2_ and 40 ppm of H_2_S simultaneously, Zn_3_(HHTP)_2_ devices exhibited a change in conductance of 36 ± 1%, as opposed to individual responses of 23.4 ± 0.3% to 40 ppm of SO_2_ and 14.9 ± 0.2% to 40 ppm of H_2_S. Based on this finding, the chemiresistive response of Zn_3_(HHTP)_2_ to binary SO_2_ and H_2_S exposure is a cumulative response to both analytes, as opposed to Ni- and Cu-based MOFs, which are selective for H_2_S. This result is further illuminated by the exposure to 5 ppm of SO_2_ and 5 ppm of H_2_S simultaneously. At this concentration, Zn_3_(HHTP)_2_ responded negligibly to H_2_S, yet it responded to SO_2_, meaning that the response during simultaneous exposure can be attributed to SO_2_. Ergo, at 5 ppm concentrations, the Cu-based MOF exhibited selectivity for H_2_S, while the Zn-based MOF exhibited selectivity for SO_2_. For all three MOFs exposed to 40 ppm analytes, crystallinity was retained, indicating structural integrity despite relatively harsh chemical environments (Figures S72–S77).

**Figure 4:**
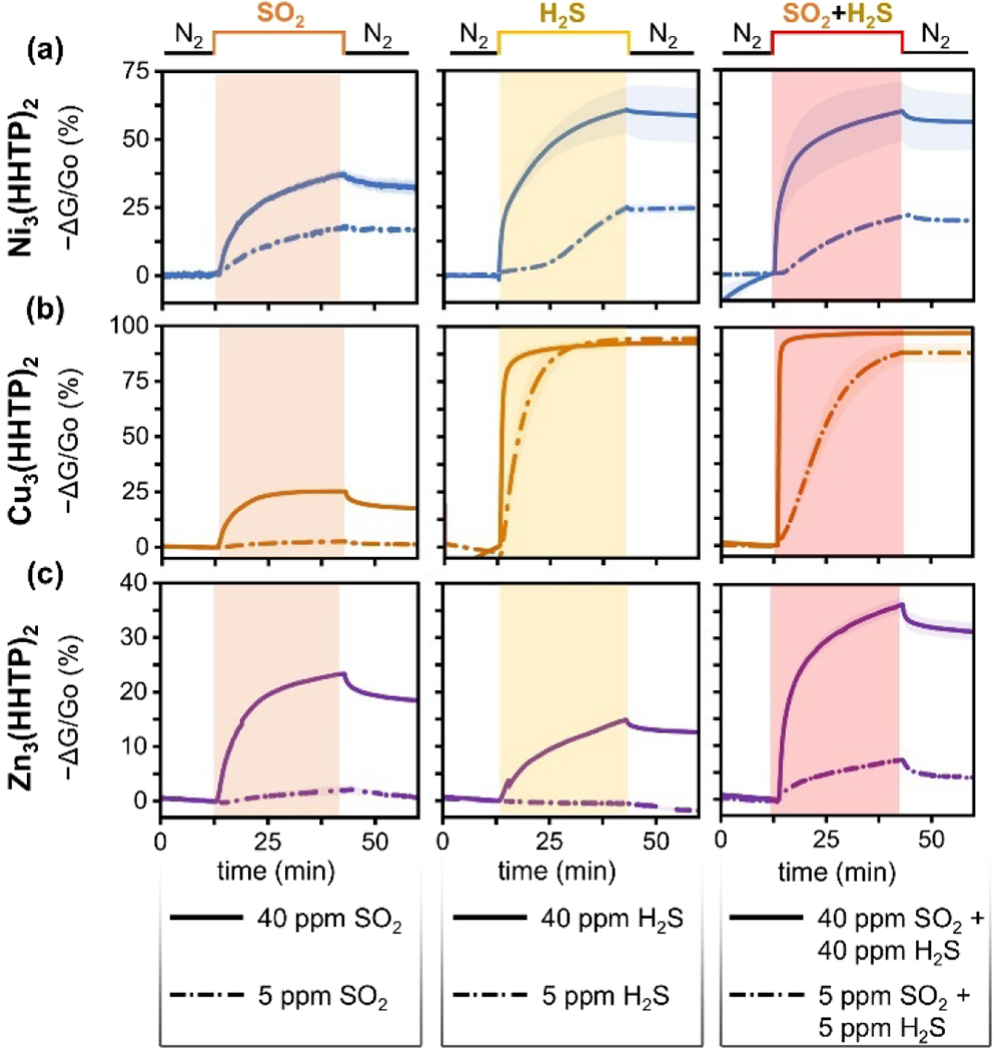
Chemiresistive sensing traces comparing the responses of (a) Ni_3_(HHTP)_2_, (b) Cu_3_(HHTP)_2_, and (c) Zn_3_(HHTP)_2_ devices to varying concentrations of SO_2_ (left, orange shading), H_2_S (middle, yellow shading), and binary SO_2_ and H_2_S mixtures (right, red shading).

Finally, when exposed to mixtures of 80 ppm of CO and 80 ppm of NO, as seen in Figure S42, all MOF devices exhibited preferential selectivity for NO over CO, indicating that the MOF materials serve as selective NO sensors in environments where both these gases are present. Overall, Ni- and Cu_3_(HHTP)_2_ selectively detected H_2_S, while Zn_3_(HHTP)_2_ was cross-reactive to SO_2_ and H_2_S, and all MOFs preferentially detected NO over CO.

### Machine Learning for Differentiation of Binary SO_2_/H_2_S Mixtures.

Next, we used machine learning to evaluate the ability to detect and discriminate binary SO_2_/H_2_S mixtures from the response of the [Ni_3_(HHTP)_2_, Cu_3_(HHTP)_2_, and Zn_3_(HHTP)_2_] sensor array. First, we employed space-filling Latin hypercube sampling to pick a diverse set of binary SO_2_/H_2_S mixture compositions to which we exposed our sensor array. The compositions are depicted in Figure 5b. We used all but two of these mixed-gas compositions to assess our ability to classify binary SO_2_/H_2_S mixtures according to the color scheme in Figure 5b: [SO_2_↑ and H_2_S↑], [SO_2_↓ and H_2_S↑], [SO_2_↑ and H_2_S↓], [SO_2_↓ and H_2_S↓], with the positive class ↑ (negative class ↓) signifying the concentration of that analyte is greater (less) than 20 ppm. We used the two remaining compositions that lie ambiguously on the classification boundary to test the uncertainty quantification of our classifier. We visualized the collection of sensor array response vectors to these binary SO_2_/H_2_S mixtures in the data matrix in Figure 5a. Again, each feature was Yeo–Johnson—then *z*-score—transformed.

**Figure 5:**
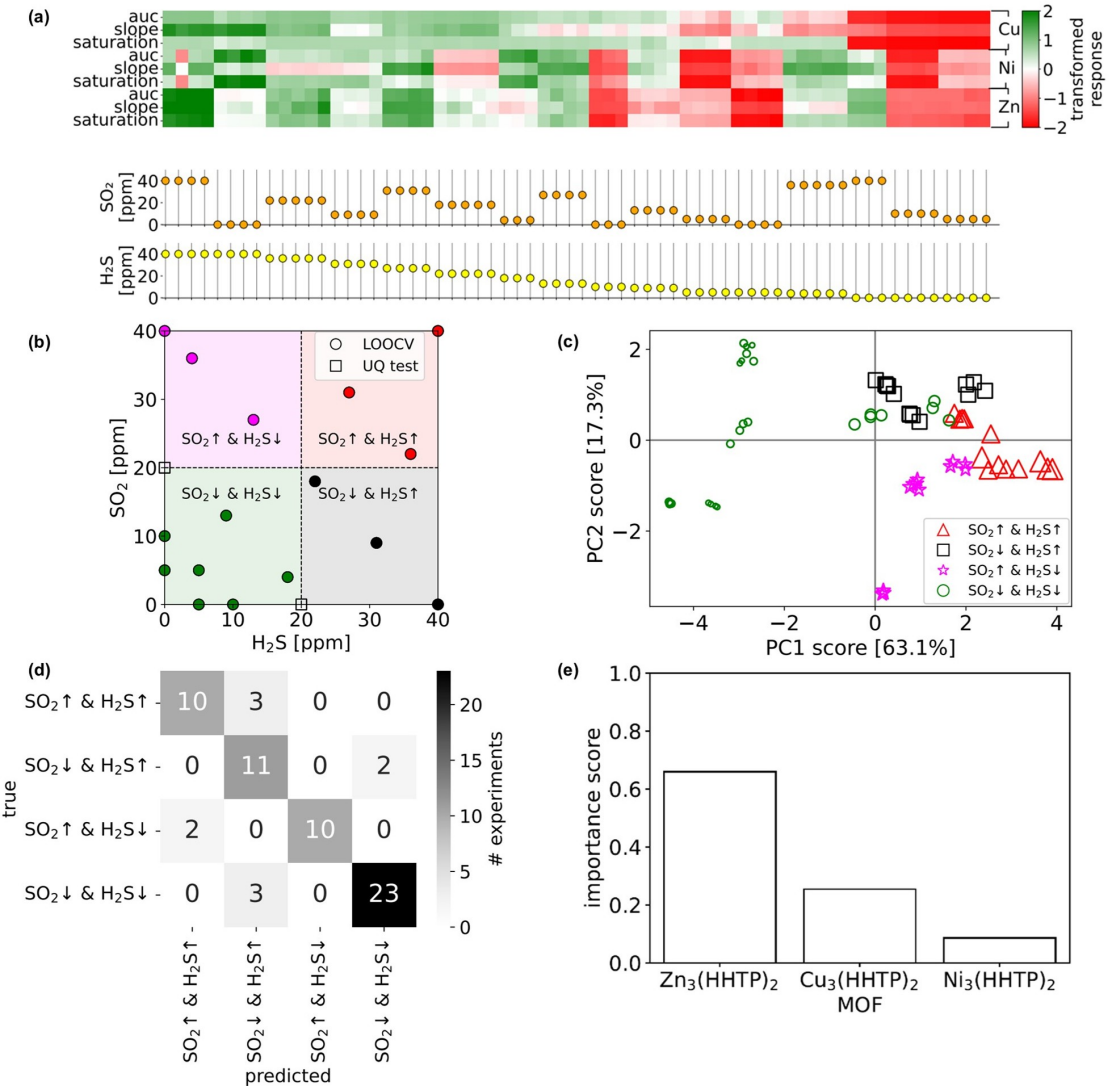
Supervised machine learning to evaluate the ability of the [Ni_3_(HHTP)_2_, Cu_3_(HHTP)_2_, and Zn_3_(HHTP)_2_] sensor array to discriminate classes of SO_2_/H_2_S mixtures in dry N_2_. (a) Heatmap of the data matrix containing response vectors of the array to binary SO_2_/H_2_S mixtures in its columns. The specific mixture compositions are shown in the scatter plot. (b) The experimental design space of SO_2_/H_2_S mixtures to which we exposed the array, partitioned into four regions defining mixture classes. (c) PCA embeddings of the response vectors, colored and shaped according to the mixture class and sized according to the total analyte concentration. (d) The confusion matrix of the random forest classifier on test data over leave-one-out cross-validation. (e) Sensor importance scores according to the random forest.

Concentration thresholds of 20 ppm were chosen for SO_2_ and H_2_S to test the ability of the sensor array to alert to various levels of gas with distinct effects on human life and health. For H_2_S, the 20 ppm threshold represents the PEL, which must not be exceeded.^23^ While 20 ppm of SO_2_ is four times the OSHA PEL, it approaches the third level of the acute exposure guideline level (AEGL-3), at which exposed individuals could experience life-threatening health effects and require more robust respiratory protection.^25,63^ The advantage of a sensor array technology capable of differentiating the identity of gases above and below 20 ppm enables pivotal assessment of the hazard present and can inform the targeted controls needed for an exposed individual.^25^

#### PCA

First, we conducted PCA on the data matrix containing the response vectors of the sensor array to binary SO_2_/H_2_S mixtures. Figure 5c shows that the PCA embeddings of the responses are quite well-separated according to the mixture class. Note that the markers are sized proportionally to the sum of the concentrations of both analytes. Intuitively, (i) the [SO_2_↑ and H_2_S↑] and [SO_2_↓ and H_2_S↓] response clusters appear at the far extremes along PC1, with [SO_2_↓ and H_2_S↓] responses moving toward the [SO_2_↑ and H_2_S↑] cluster as the sum of the analyte concentrations increases; and (ii) the [SO_2_↓ and H_2_S↑] and [SO_2_↑ and H_2_S↓] clusters appear on the positive and negative half-plane with respect to PC2. Hence, we loosely interpret PC1 as capturing the total concentration of analytes and PC2 as capturing the difference between SO_2_ and H_2_S. This foreshadows that we can discriminate between these four classes of SO_2_/H_2_S mixtures based on the response of the sensor array.

#### Random Forest Classification

Next, we turned to supervised machine learning to construct a map from the response vector of the [Ni_3_(HHTP)_2_, Cu_3_(HHTP)_2_, Zn_3_(HHTP)_2_] sensor array to a prediction of the SO_2_/H_2_S mixture class that caused it.^64,65^ This map, constructed from data constituting (*x*, *c*) pairs, is not only practically useful but also serves as a means for us to directly test our ability to discriminate between the SO_2_/H_2_S mixture classes (i.e., obtain false positive and false negative rates) from the response pattern of the array through cross-validation. Specifically, we trained a two-output (one for SO_2_, one for H_2_S) random forest classifier that takes as input the sensor array response vector *x* and outputs a predicted SO_2_/H_2_S mixture class *c* ∈ {[SO_2_↑ and H_2_S↑], [SO_2_↓ and H_2_S↑], [SO_2_↑ and H_2_S↓], [SO_2_↓ and H_2_S↓]}.

A random forest is composed of an ensemble of binary, decorrelated decision trees.^66^ The root node of each tree takes as its input the sensor array response vector *x*. Each internal node decides which of its two child nodes to send the vector to next, based on whether a particular response feature of a particular sensor exceeds a threshold. Once a leaf node is reached, a predicted class is assigned to the response vector based on the classes of the training data that land there. Our random forest consists of 500 trees, which are diverse because (i) each tree is grown on a bootstrap (sub)­sample of the training responses and (ii) each internal node is allowed to split the training data based on only a random subset of the nine features. Each tree in the ensemble casts a vote on the class of the input response vector, and the decision rule for the random forest is to pick the class with the most votes. The distribution of votes by the trees in the forest among the classes quantifies the uncertainty of the prediction. When growing a decision tree, at each internal node, the selection of the feature and threshold used for splitting the training data is chosen to maximize the class purity of the training data that falls into the two child nodes after the split; hence, we can allocate gains in purity, as splits are made, to specific features. The accumulated purity gain thanks to a feature is a measure of its importance.

Since our data set is relatively small (examples of the response to exposure to 16 distinct compositions), we made two decisions to prevent our classifier from overfitting the data. First, we extracted only three hand-crafted features from the sensing trace of each MOF using our domain knowledge; based on prior work, the magnitude of the response and the initial rate of response tend to be indicative of the identity and concentration of the gas.^43,51,67–69^ We also included the area under the sensing trace with the aim of capturing different response shapes with the same magnitude and initial slope. Our parsimonious feature engineering approach contrasts with learning informative features from the raw time-series data constituting the sensing trace, which is achievable by [data-hungry] convolutional^70,71^ or recurrent^72^ neural networks when provided with sufficient training data.^73^ Second, we employ a random forest for our classifier,^74^ which has been utilized for gas sensor arrays before.^75,76^ Compared to neural networks, random forests are less prone to overfitting (via averaging an ensemble of decorrelated trees) and tend to perform robustly out-of-the-box, outperforming neural networks on small tabular data.^64,77–79^

We then evaluated the performance of the *x*→*c* map (input: sensor array response vector; output: SO_2_/H_2_S mixture class) constituted by a two-output random forest via a confusion matrix obtained from leave-one-out cross-validation (LOOCV). During LOOCV, we assigned all (*x*, *c*) pairs in the data that correspond to a particular concentration of SO_2_ and H_2_S (multiple pairs, owing to replicates) to a test set and left the remaining (*x*, *c*) pairs as training data. Next, we trained a random forest on the training data and then used this random forest to predict the mixture class of the sensor response vectors in the test set. We iterated this process until each mixture composition in Figure 5a was used as a test set once. The confusion matrix in Figure 5d summarizes the performance of the random forest classifier on the test data for the LOOCV procedure. It compares the true mixture class labels in the test sets with the predicted labels. Most (*x*, *c*) test pairs fall on the diagonal, where the random forest correctly predicted the mixture class. The true positive rate (recall), false positive rate, and precision can be estimated for each mixture class, e.g., for [SO_2_↑ and H_2_S↑]: 10/13, 2/51, and 10/12, respectively (we expand on these metrics in Section SXII.) In conclusion, the random forest classifier can discriminate among the four SO_2_/H_2_S mixture classes using the sensor array response vectors with quite high recall, high precision, and low false positive rate.

Next, we computed the average feature importances over the leave-one-out cross-validation procedure^64^ to obtain sensor importance scores in Figure 5e. To obtain an importance score for each sensor, we summed the feature importance scores of its three response features. We observe that the Zn_3_(HHTP)_2_ sensor provided most of the information used to discriminate between the SO_2_/H_2_S mixture class. Satisfyingly, this is consistent with the responses in Figure 4 that highlight the cross-reactivity of Zn_3_(HHTP)_2_ to both SO_2_ and H_2_S, while the Cu- and Ni_3_(HHTP)_2_ sensors were both generally selective for H_2_S. However, the Cu- and Ni_3_(HHTP)_2_ sensors still contributed information for the classification.

Finally, we demonstrated uncertainty quantification by random forests on the seven sensor array responses to either 20 ppm of SO_2_ or 20 ppm of H_2_S that lie on the classification boundary. For the four responses to 20 ppm of SO_2_, the trees split their votes roughly 40%/40%/20% among [SO_2_↑ and H_2_S↓]/[SO_2_↓ and H_2_S↑]/[SO_2_↓ and H_2_S↓]. This high level of disagreement would be expected for a well uncertainty-calibrated random forest. For the three responses to 20 ppm of H_2_S, the trees split their votes roughly 90%/10% among [SO_2_↓ and H_2_S↑]/[SO_2_↓ and H_2_S↓]. As expected, the trees correctly cast their votes only for the two mixture classes that do not include SO_2_; unexpectedly, however, the forest is falsely confident. Generally, uncertainty quantification for machine learning on gas sensors is useful for making downstream decisions (e.g., evacuating a room) based on the confidence of the model’s prediction.^80^

### Spectroscopic Characterization of Material–Analyte Interactions

Inspired by the wide range of chemiresistive responses observed in the MOF array across individual doses of SO_2_ and H_2_S and their mixtures thereof, we endeavored to investigate the underlying material–analyte interactions. While there are examples of variations in sensing performance based on the crystallite size and morphology of triphenylene-based MOFs,^81^ we hypothesize that the dominant features influencing sensing profiles for the MOFs explored in this study are the metal center identity and the MOF packing pattern. All three MOFs exhibited crystals in the nanoscale range, and the sensing results we observe for Cu_3_(HHTP)_2_ with H_2_S align with previous reports despite differences in morphology between the two materials (i.e., nanoflakes in our study and nanowires in the published study).^39^ To test this hypothesis, we utilized *in situ* DRIFTS and *ex situ* XPS to gain insight into the chemical and oxidative changes of the MOFs as a direct result of analyte exposure, aiming to explain the emergent chemiresistive response.

#### SO_2_ Exposure

Ni_3_(HHTP)_2_ exhibited superior SO_2_ interaction compared to Cu- and Zn_3_(HHTP)_2_ as demonstrated by chemiresistive sensing results, DRIFTS, and XPS analyses, which we hypothesized to be due to the intercalated layer present in the Ni-based MOF. Chemiresistive measurements revealed that Ni_3_(HHTP)_2_ devices exhibited the highest sensitivity for SO_2_, while both Cu- and Zn_3_(HHTP)_2_ devices showed commensurately lower responses. These chemiresistive results parallel the results of DRIFTS spectra; after a 20 min 1% SO_2_ exposure followed by 60 min of purging with N_2_, Ni_3_(HHTP)_2_ uniquely retained key vibrational bands at 1366 and 1154 cm^–1^, indicating the presence of adsorbed SO_2_ within the framework (Figure 6a).^82^ Furthermore, Ni_3_(HHTP)_2_ displayed additional peaks at 1257 and 1221 cm^–1^, corresponding to a second SO_2_ adsorption site, as well as sharp peaks at 1033 and 1000 cm^–1^, confirming the transformation of SO_2_ into bound sulfate (SO_4_^2–^) (Figure 6a).^83,84^ The presence of SO_4_^2–^ on Ni_3_(HHTP)_2_ following exposure to SO_2_ was confirmed by XPS (Figure S59).^85^ XPS analysis also revealed that upon SO_2_ exposure, Cu- and Zn_3_(HHTP)_2_ materials contained SO_4_^2–^ and a 58:17:14:11 ratio of SO_4_^2–^:sulfite (SO_3_^2–^):polysulfides (S_*x*_):sulfide (S^2–^), respectively^36,46,85–87^ (Figures S63 and S67). Additionally, 1% SO_2_ DRIFTS of Cu_3_(HHTP)_2_ revealed small peaks at 1260 and 1037 cm^–1^, indicative of chemisorbed SO_2_ and bound SO_4_^2–^, respectively.^82,83^ Finally, DRIFTS exhibited a negative-going broad band at around 3600 cm^–1^ for all three MOFs (Figures S46, S48, and S50), which we attributed to the removal of water within the MOF framework. It is possible that the water serves as a source for the oxidation of SO_2_ to SO_4_^2–^.^88^ This mechanism of oxidation rationalizes the improved performance of Ni_3_(HHTP)_2_ compared to that of the other two MOFs. As evidenced from the intercalated packing structure of Ni_3_(HHTP)_2_ and the IR spectra of the pristine MOF (Figure S14), Ni_3_(HHTP)_2_ contains a larger quantity of aqua ligands compared to Cu- and Zn_3_(HHTP)_2_ due to the inherent aqua-capped metal catecholes present in the intercalated layer of Ni-HHTP monomeric complexes.^89^ With a larger number of hydroxyl sites for the SO_2_ interaction, the Ni_3_(HHTP)_2_ packing pattern rationalizes improved SO_2_ detection. Additionally, all three MOFs exhibited increases in bands around 1670–1708 cm^–1^, which we assigned to HHTP ligand of the MOF indicating that the bonds were strengthened upon exposure to the analyte.

**Figure 6:**
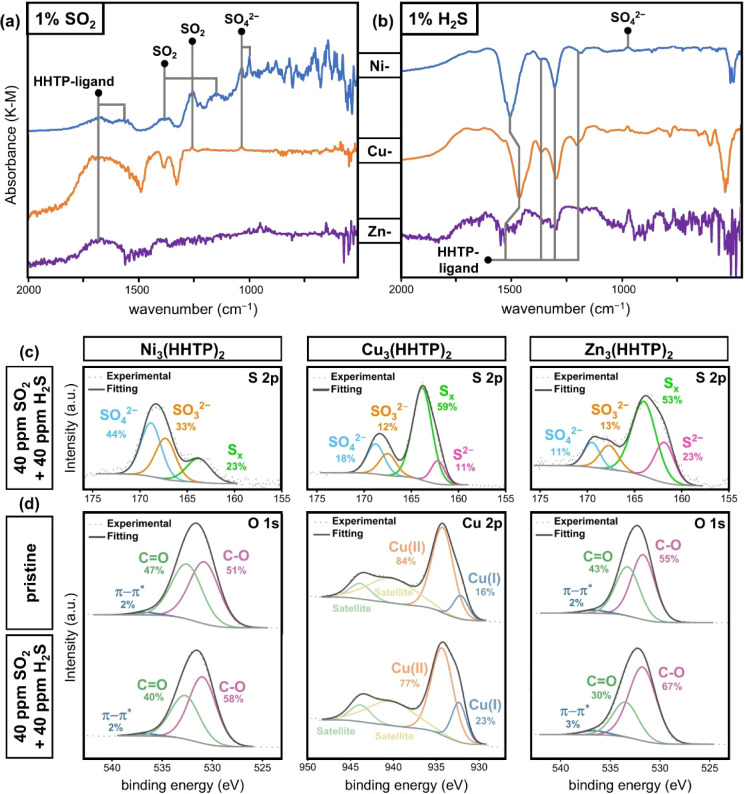
Spectroscopic investigation into material–analyte interactions. DRIFTS difference spectra of Ni_3_(HHTP)_2_ (blue), Cu_3_(HHTP)_2_ (orange), and Zn_3_(HHTP)_2_ (purple) following exposure to (a) 1% SO_2_ or (b) 1% H_2_S followed by 60 min of purging with dry N_2_ (note: absorbance is normalized to enable resolution of spectral features). (c) XPS analysis on the S 2p region for M_3_(HHTP)_2_ (M = Ni, Cu, Zn) (left to right) following dual exposure to 40 ppm of SO_2_ and 40 ppm of H_2_S in dry N_2_ for 1 h. (d) XPS analysis on key sites of reduction in M_3_(HHTP)_2_ (M = Ni, Cu, Zn) (left to right). The top plot features key regions in pristine MOFs, and the bottom plot features the same region following dual exposure to 40 ppm of SO_2_ and 40 ppm of H_2_S in dry N_2_ for 1 h.

#### H_2_S Exposure

As exhibited in the chemiresistive data, the sensitivity of the MOFs to H_2_S followed a subsequent trend: Cu_3_(HHTP)_2_ > Ni_3_(HHTP)_2_ > Zn_3_(HHTP)_2_. This trend was also reflected in the DRIFTS results, where the relative intensity of absorbance changes paralleled the chemiresistive response of the devices (Figures 6b and S58). The correlation between changes in device resistance and DRIFTS absorbance further supports the hypothesis that the material-specific crystal structure directs chemiresistive sensing. The strong response of Cu_3_(HHTP)_2_ to ppm-level H_2_S has previously been investigated and attributed to the redox lability of the Cu­(II) bisdioxolene linkage.^46^ Our XPS results support this claim, as the Cu­(II):Cu­(I) content in Cu_3_(HHTP)_2_ decreased from 84:16 to 74:26 upon exposure to 40 ppm of H_2_S (Figure S64). This reduction at the Cu center facilitated H_2_S oxidation primarily to S_*x*_ or S^2–^ as seen in the S 2p XPS region (Figure S63).^36,46,87^ In contrast, no changes in metal oxidation states were observed for Ni- and Zn_3_(HHTP)_2_ following H_2_S exposure (Figures S60 and S68). Instead, for these two MOFs, the framework reduction occurred at the HHTP ligand, as indicated by an increase in C–O:C=O compared to the pristine MOFs (Figures S61 and S69). Finally, negative-going DRIFTS bands corresponding to characteristic MOF peaks in both position and shape indicate overall material degradation, as the highly reducing H_2_S in high concentration disrupts long-range crystal order (Figure 6b).^90^ This structural degradation postanalyte exposure was verified using PXRD (see Section SXV) and was previously reported for Cu_3_(HHTP)_2_.^46^

#### SO_2_ and H_2_S Exposure

Upon dual exposure to SO_2_ and H_2_S, Ni_3_(HHTP)_2_ mainly directed analyte oxidation to SO_4_^2–^, while both Cu- and Zn_3_(HHTP)_2_ directed analytes to polysulfides (Figure 6c). This difference in analyte transformation likely occurred due to the higher water content of Ni_3_(HHTP)_2_ compared with the other MOFs with slip-parallel packing. However, despite the fact that Ni_3_(HHTP)_2_ exhibited the most optimal SO_2_ sensitivity of the three MOFs tested, the material retained a selective response for H_2_S in mixtures due to the strong reducing effect of H_2_S on the framework, as evidenced by DRIFTS (Figure 6b). In the presence of the binary mixture, Cu_3_(HHTP)_2_ exhibited highly selective H_2_S sensitivity due to labile, redox-active Cu centers (Figure 6d) and the irreversible formation of S_*x*_ and S^2–^. This strong interaction caused the most dramatic irreversible changes to the framework structure and oxidation compared with Ni- and Zn_3_(HHTP)_2_. Finally, in the presence of both analytes, the sliding-scale analyte selectivity of Zn_3_(HHTP)_2_ dependent on the concentration range emerged due to diminished reactivity of the MOF to the analytes. As Zn_3_(HHTP)_2_ exhibited moderate reactivity with both analytes, the emergent response upon dual exposure cumulatively accounted for both interactions without oversaturation from one strongly interacting species. Despite the differences in crystal sizes among the three MOFs tested, the overarching trends in spectroscopic interaction, reactivity, and analyte selectivity can be explained by the metal node identity and MOF packing pattern.

#### CO and NO

Interactions between MOF materials and the mixture of CO and NO were not directly investigated due to the overwhelming selectivity of all three materials for NO over CO. Based on literature precedent, NO strongly interacts with HHTP-based MOFs to form metal-nitrosyl complexes and transform NO to NO_*x*_ species, thereby outcompeting the stable, unreactive CO.^30,46^ For a full description of spectroscopic procedures and findings, see Section SXIV.

## Conclusion

This work examines an M_3_(HHTP)_2_ (M = Ni, Cu, Zn) chemiresistive sensor array capable of detecting and discriminating toxic analytes, namely CO, NH_3_, SO_2_, H_2_S, and NO, at ppm-level concentrations in dry N_2_. Moreover, we showed that the array is capable of discriminating SO_2_/H_2_S mixtures, despite cross-sensitivity. Both tasks relied on machine learning algorithms to parse the high-dimensional response patterns of the array. Furthermore, we systematically studied the material–analyte interactions and thereby elucidated key MOF properties facilitating the efficient detection and differentiation of the toxic gases. The results address a continued challenge in the literature and the commercial sensing field at large: the limitations that sensor cross-sensitivities place on reliable toxic gas identification. By leveraging differences in metal identity, framework packing, and hydration, the MOF sensor array not only successfully differentiates the suite of five analytes of biological and toxicological relevance but also discriminates an analyte mixture.

Distinct chemiresistive responses arose from specific interactions between the three MOFs and the target gases. The Ni- and Cu_3_(HHTP)_2_ materials exhibited selective detection of H_2_S over SO_2_, while Zn_3_(HHTP)_2_ exhibited variable selectivity depending on the analyte concentration. The selectivity was probed using *in situ* DRIFTS and *ex situ* XPS to monitor the changes in the chemical composition and oxidation within the MOFs during and following analyte exposure. Our studies demonstrate that of the three MOFs tested, Ni_3_(HHTP)_2_ exhibited the strongest response to SO_2_ over the course of 30 min exposure due to the availability of adsorption sites in the intercalated layer facilitating SO_2_ oxidation. Additionally, our results aligned with previous reports demonstrating the importance of the redox-labile copper linkage for the interaction of analytes with Cu_3_(HHTP)_2_. Finally, based on spectroscopic findings, the dominant site for reduction upon S-containing analyte exposure to Ni- and Zn_3_(HHTP)_2_ occurs at the HHTP ligand. This work presents convincing evidence on how differing degrees of reactivity between S-containing analytes and structurally distinct MOFs can be utilized in arrays for analyte differentiation.

This work paves the way toward the utilization of MOF-based sensor arrays for mixture discrimination. Investigation of framework-directed analyte selectivity will reveal key insights into MOF–analyte interactions. Future work on this class of materials for mixture discrimination should test array function in the presence of other concomitant analytes and under realistic conditions. As seen in this work, the role of aqua ligands and the MOF hydration level directed analyte interaction and sensing performance. While not explored in this work, understanding the MOF sensor array performance in the presence of humidity is imperative for widespread adoption. We hypothesize that the MOFs will perform well in humidity based on previous studies.^88^ The use of machine learning has shown the ability to aid in analyte discrimination in challenging conditions of humidity and will likely aid in binary mixture discrimination in real-world conditions.^91,92^ Overall, this work provides valuable insights to advance the field of MOF-based chemiresistive sensors closer to the functional reality of operating in complex mixtures.

## Supplementary Material



## Notes

Additional experimental details, material characterization, chemiresistive sensing traces, machine learning data, and spectroscopic analyses of material–analyte interactions (PDF)
