# Supplementary material for: Metal–Organic Framework-Based Chemiresistive Array Paired with Machine Learning Algorithms for the Detection and Differentiation of Toxic Gases
Source: ACS Sens. 2025 Oct 6;10(10):7787–98. doi: 10.1021/acssensors.5c02182 (PMC12560124; doi:10.1021/acssensors.5c02182)
Supplement: Supplementary file 1 [file se5c02182_si_001.pdf]

Metal−Organic Framework-Based Chemiresistive Array Paired with Machine Learning Algorithms for the Detection and Differentiation of Toxic Gases

Georganna Benedetto,[1] Patrick Damacet,[1] Elissa O. Shehayeb,[1] Gbenga Fabusola,[2] Cory M. Simon,[2,*] and Katherine A. Mirica[1,*]

[1]Dartmouth College, Department of Chemistry, Hanover, NH 03755
[2]Oregon State University, School of Chemical, Biological, and Environmental Engineering, Corvallis, OR 97331
*email: cory.simon@oregonstate.edu, katherine.a.mirica@dartmouth.edu

SUPPORTING INFORMATION

**I.**	**Materials and Methods**	**S2**
**II.**	**Synthesis of $M_3(HHTP)_2$ (M=Ni, Cu, Zn)**	**S2**
**III.**	**Powder X-ray Diffraction (pXRD) of MOFs**	**S4**
**IV.**	**Electron Microscopy of $M_3(HHTP)_2$ (M=Ni, Cu, Zn)**	**S6**
**V.**	**Energy Dispersive X-ray Analysis of $M_3(HHTP)_2$ (M=Ni, Cu, Zn)**	**S7**
**VI.**	**Transmission Electron Microscopy of $M_3(HHTP)_2$ (M=Ni, Cu, Zn)**	**S9**
**VII.**	**Thermal Gravimetric Analysis**	**S12**
**VIII.**	**ATR-IR Spectra of MOFs**	**S12**
**IX.**	**Conductivity Analysis of MOFs**	**S13**
**X.**	**Brunauer−Emmett−Teller (BET) Analysis of MOFs**	**S14**
**XI**	**Chemiresistive Gas Sensing**	**S15**
**XII.**	**Metrics for SO_2/H_2S Mixture Classification**	**S41**
**XIII.**	**Machine Learning Regression & Feature Analysis**	**S43**
**XIV.**	**Spectroscopic Assessment of Material−Analyte Interactions**	**S45**
**XV.**	**PXRD post analyte exposure**	**S74**
**XVI.**	**References**	**S81**

I. Materials and methods

All reagents and solvents were purchased from commercial sources and used as received without purification. Nickel(II) acetate tetrahydrate (>95 %) and sodium acetate (>98.5 %) were acquired from TCI. Copper nitrate trihydrate (99 %), zinc acetate hexahydrate (99%), ammonia solution (35%), N,N-Dimethylformamide (>99.5 %, GC grade), and acetone (ACS reagent, ≥99.5 %) were purchased from Fisher Scientific. 2,3,6,7,10,11-hexahydroxytriphenylene (HHTP, 97%) and ethanol were acquired from Ambeed and Koptec respectively.

Powder X-ray diffraction (PXRD) spectra were collected on a Rigaku MiniFlex Powder X-ray Diffractometer equipped with a Cu 600 W (40 kV, 15 mA, l = 1.54 Å) radiation source following background subtraction. The range between 3° and 40° 2θ was scanned with a step size of 0.02° and a scan rate of 4° per minute. Scanning Electron Microscopy (SEM) was performed on a Thermo Scientific Helios 5 CX DualBeam scanning electron microscope.

II. Synthesis of $M_3(HHTP)_2$ (M=Ni, Cu, Zn)

Synthesis of $Ni_3(HHTP)_2$

$Ni_3(HHTP)_2$ was synthesized under conditions similar to those reported in the literature.[1] In a 20 mL scintillation vial, 20 mg of $Ni(OAc)_2{\cdot}4H_2O$ (0.08 mmol, 2.0 eq) was dissolved in 8 mL DI water and the solution was sonicated for a few minutes. 14 mg of HHTP (0.04 mmol, 1.0 eq) was added to the nickel solution and the resulting mixture was sonicated for 5 minutes. The vial was then capped and placed in a preheated isothermal oven set at 85 °C for 12 hours. The resulting black precipitate was filtered under vacuum, washed with DI water (4 x 10 mL), ethanol (2 x 10 mL), and acetone (2 x 20 mL), before being dried in a vacuum oven set at 73 °C for 24 hours.

Synthesis of $Cu_3(HHTP)_2$

$Cu_3(HHTP)_2$ was prepared by a similar synthesis route previously reported in literature.[2] In brief, 63.5 mg of $Cu(NO_3)_2.3H_2O$ (0.26 mmol, 1.7 eq.) and 0.41 mL of a 35% concentrated ammonia solution (7.5 mmol, 47 eq) were added to a 20 mL scintillation vial containing 1 mL of DI water. Following sonication for 5 minutes, the solution was added dropwise over a period of 2 minutes to a mixture previously prepared by dissolving 50.1 mg of HHTP (0.15 mmol, 1.0 eq) in

4.2 mL DI water. The resulting solution was sonicated for 5 minutes, capped, and subsequently placed in a preheated isothermal oven set at 75 °C overnight. The resulting dark blue precipitate was filtered under vacuum, washed with DI water (3 x 20 mL), ethanol (1 x 20 mL), and acetone (3 x 20 mL), before being dried in a vacuum oven set at 73 °C for 24 hours.

Synthesis of $Zn_3(HHTP)_2$

$Zn_3(HHTP)_2$ was synthesized under conditions similar to those reported in the literature with some modifications.[3] In brief, 24 mg of $Zn(NO_3)_2.6H_2O$ (0.08 mmol, 1 eq) was dissolved in 6 mL of DI water in a 20 mL scintillation vial. 1 mL of 0.25 M of NaOAc modulator followed by 26 mg of HHTP (0.08 mmol, 1 eq) dispersed in 6 mL of DI water were added to the zinc solution. The resulting mixture was sonicated for 5 minutes before being allowed to stir on a hot plot preheated at 85 °C for 2 hours. The resulting precipitate was filtered under vacuum, washed with DI water (2 x 20 mL) and acetone (2 x 20 mL), before being dried in a vacuum oven set at 73 °C for 24 hours.

Activation of MOFs prior to characterization and sensing experiments

All MOFs were activated using the same process. Briefly, the MOF crystals were soaked in ethanol for 2 days, with the solvent being exchanged with fresh ethanol every 12 hours. The solvent was then exchanged with acetone following the same process. The resulting crystals were finally dried for 48 hours in a vacuum oven set at 75°C prior to structural and morphological characterization.

III. Powder x-ray diffraction (pXRD) of MOFs

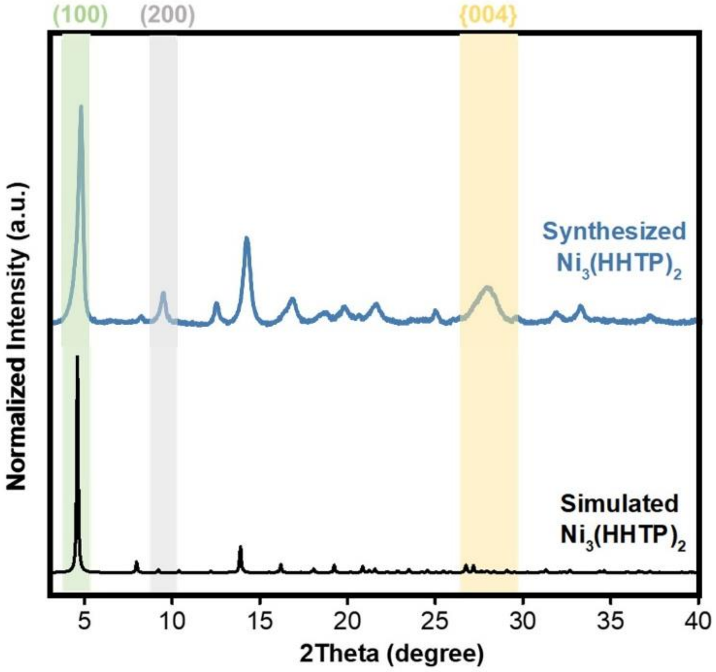

Figure S1. Powder X-ray diffraction patterns of $Ni_3(HHTP)_2$ particles following activation.

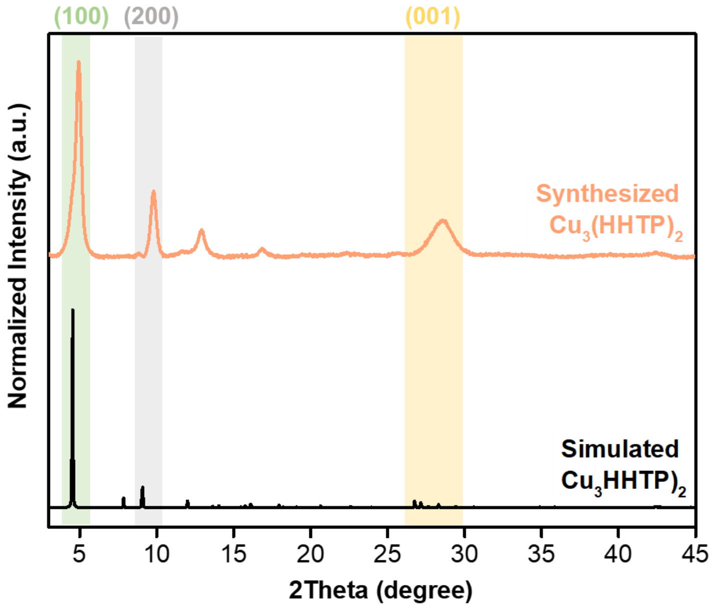

Figure S2. Powder X-ray diffraction patterns of $Cu_3(HHTP)_2$ particles following activation.

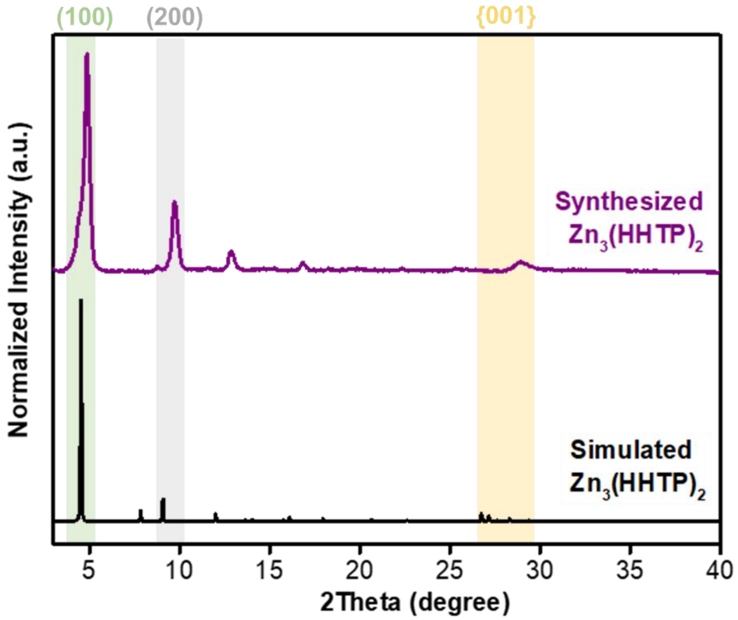

Figure S3. Powder X-ray diffraction patterns of $Zn_3(HHTP)_2$ particles following activation.

IV. Electron Microscopy of $M_3(HHTP)_2$ (M=Ni, Cu, Zn)

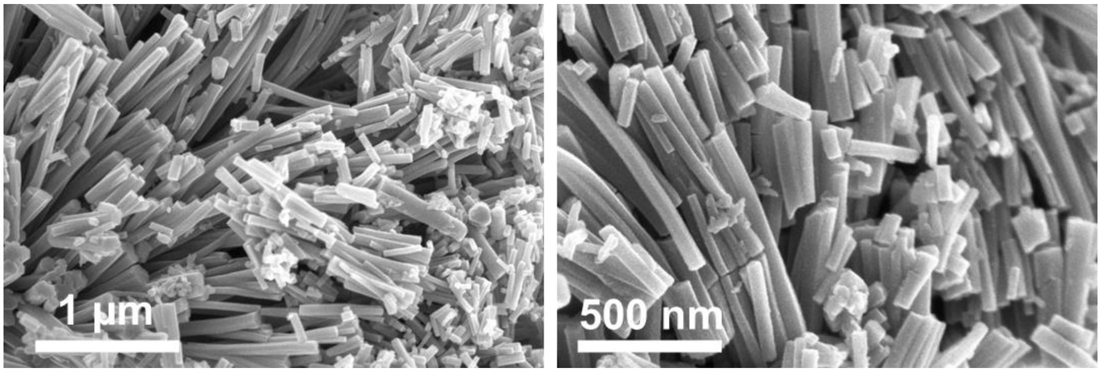

Figure S4. SEM micrographs of $Ni_3(HHTP)_2$ obtained using an acceleration voltage of 2.0 kV and a working distance of 4.1 mm.

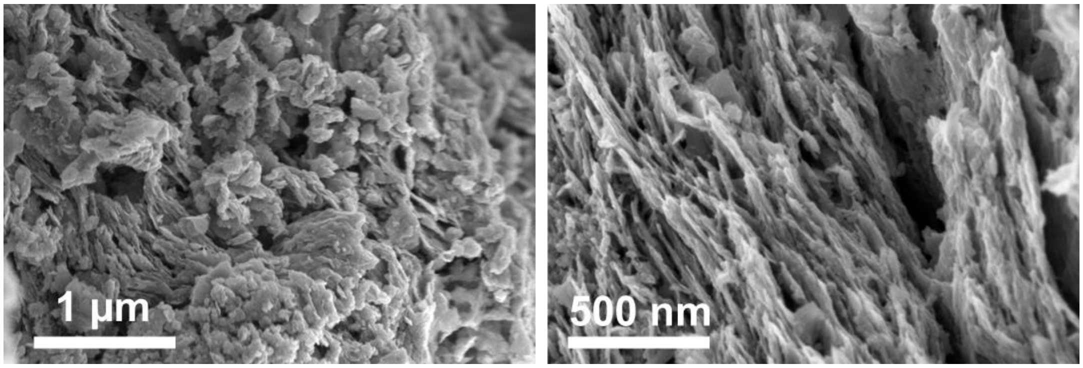

Figure S5. SEM micrographs of $Cu_3(HHTP)_2$ obtained using an acceleration voltage of 2.0 kV and a working distance of 4.1 mm.

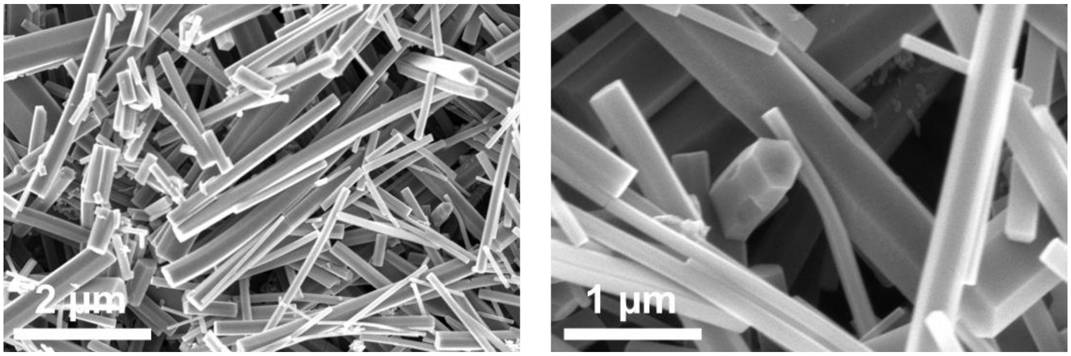

Figure S6. SEM micrographs of $Zn_3(HHTP)_2$ obtained using an acceleration voltage of 2.0 kV and a working distance of 4.1 mm.

V. Energy Dispersive X-ray Analysis of $M_3(HHTP)_2$ (M=Ni, Cu, Zn)

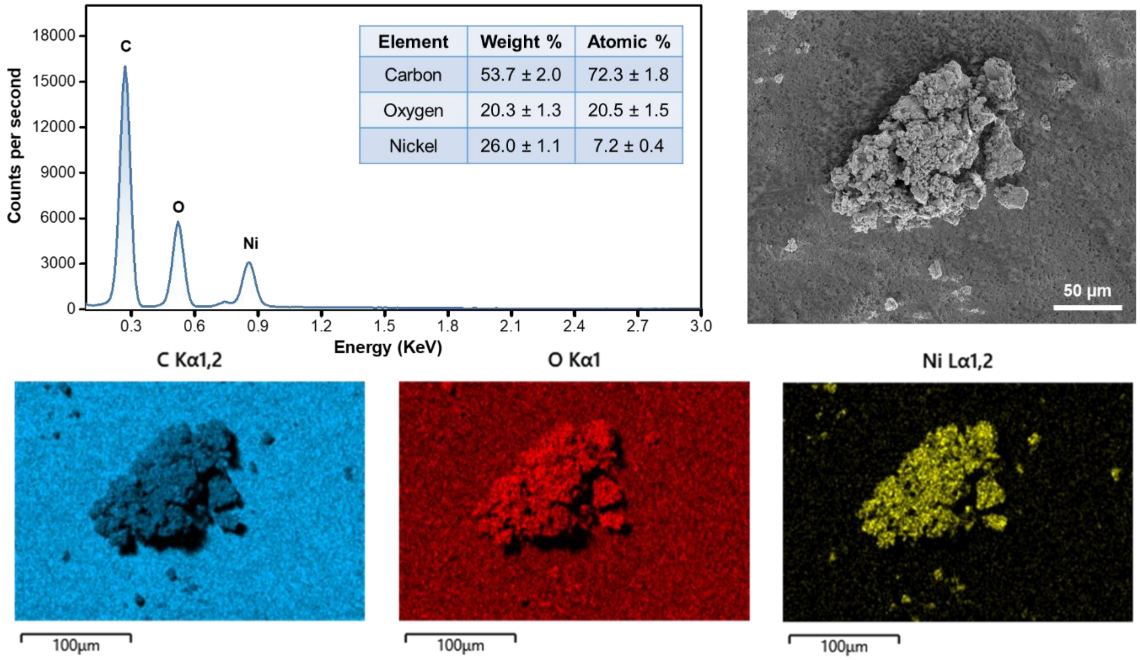

Figure S7. EDX spectrum and elemental mapping images of $Ni_3(HHTP)_2$.

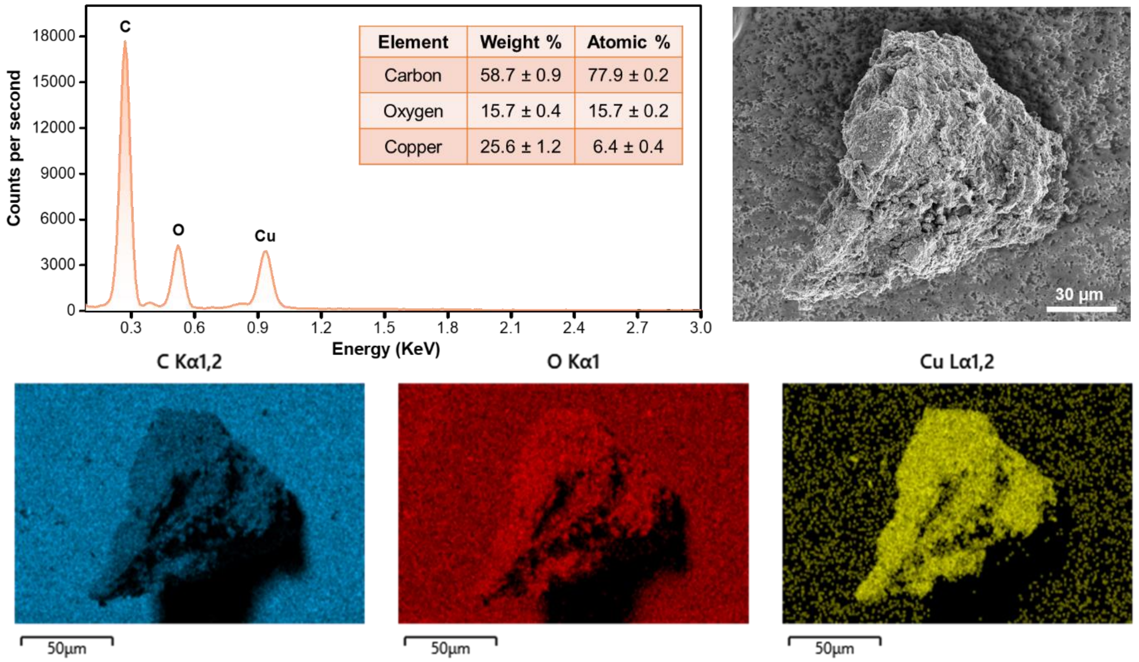

Figure S8. EDX spectrum and elemental mapping images of $Cu_3(HHTP)_2$.

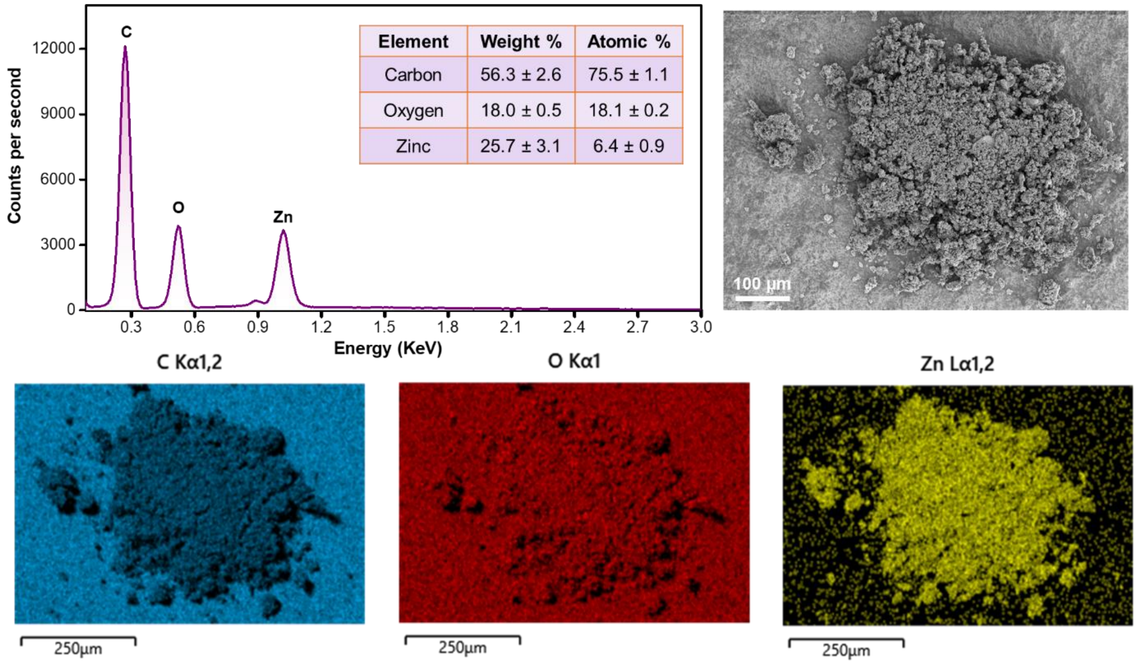

Figure S9. EDX spectrum and elemental mapping images of $Zn_3(HHTP)_2$.

VI. Transmission Electron Microscopy of $M_3(HHTP)_2$ (M=Ni, Cu, Zn)

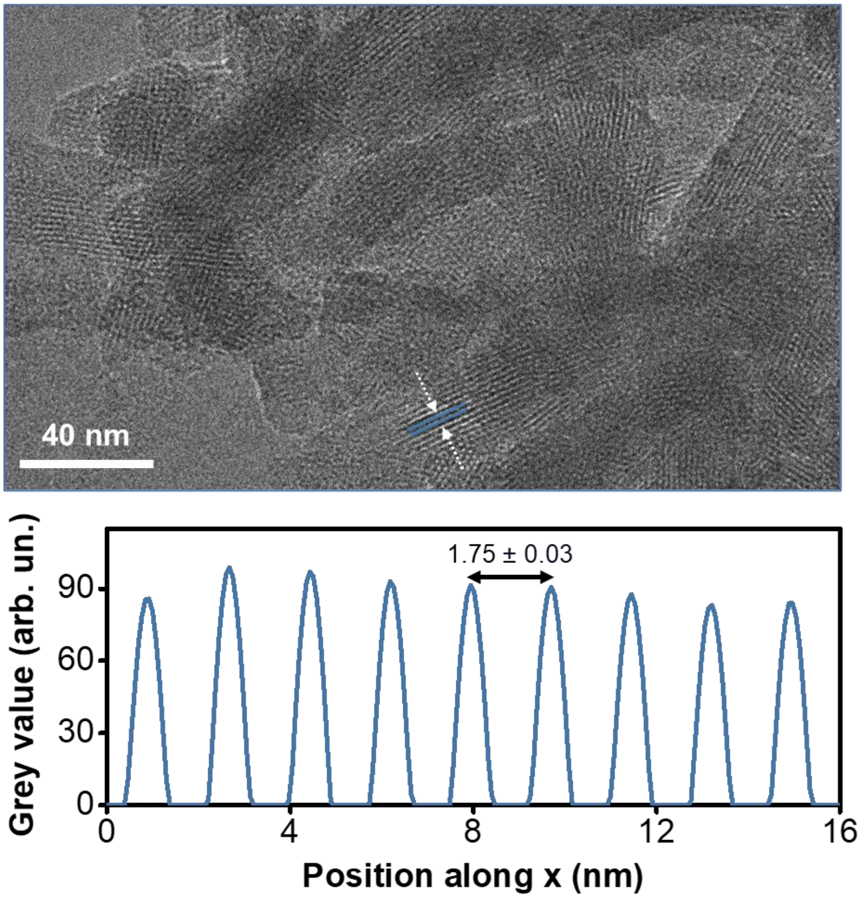

Figure S10. HR-TEM micrograph of $Ni_3(HHTP)_2$ along the (100) direction (top) and line intensity profile of the lattice planes (bottom). The interplanar distance is calculated to be 1.75 ± 0.03 nm.

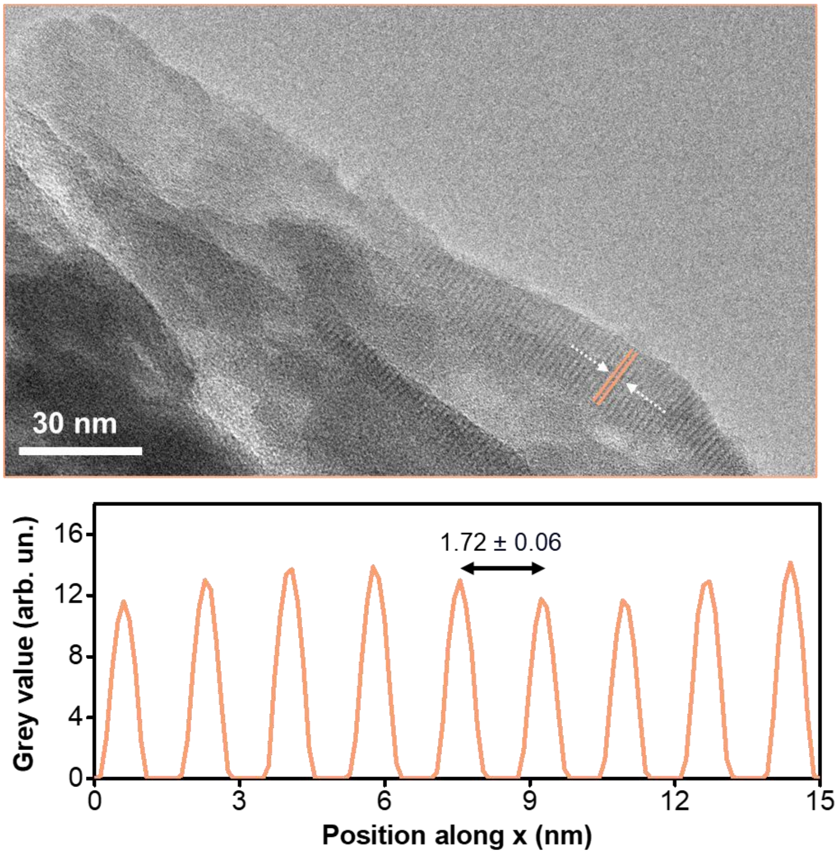

Figure S11. HR-TEM micrograph of $Cu_3(HHTP)_2$ along the (100) direction (top) and line intensity profile of the lattice planes (bottom). The interplanar distance is calculated to be 1.72 ± 0.06 nm.

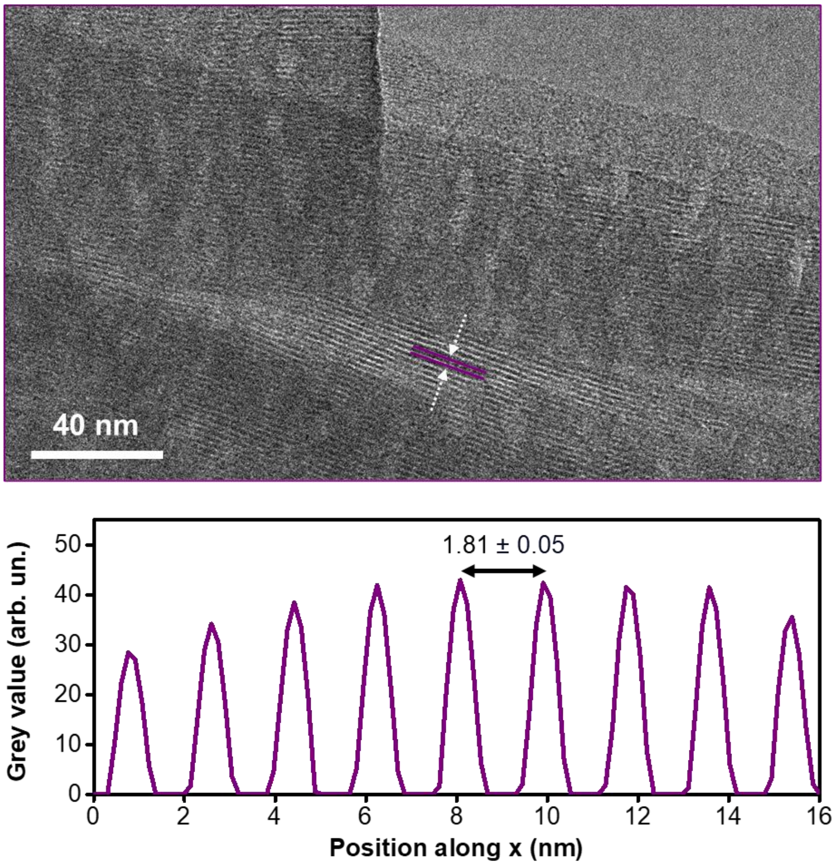

Figure S12. HR-TEM micrograph of $Zn_3(HHTP)_2$ along the (100) direction (top) and line intensity profile of the lattice planes (bottom). The interplanar distance is calculated to be 1.81 ± 0.05 nm.

VII. Thermal Gravimetric Analysis

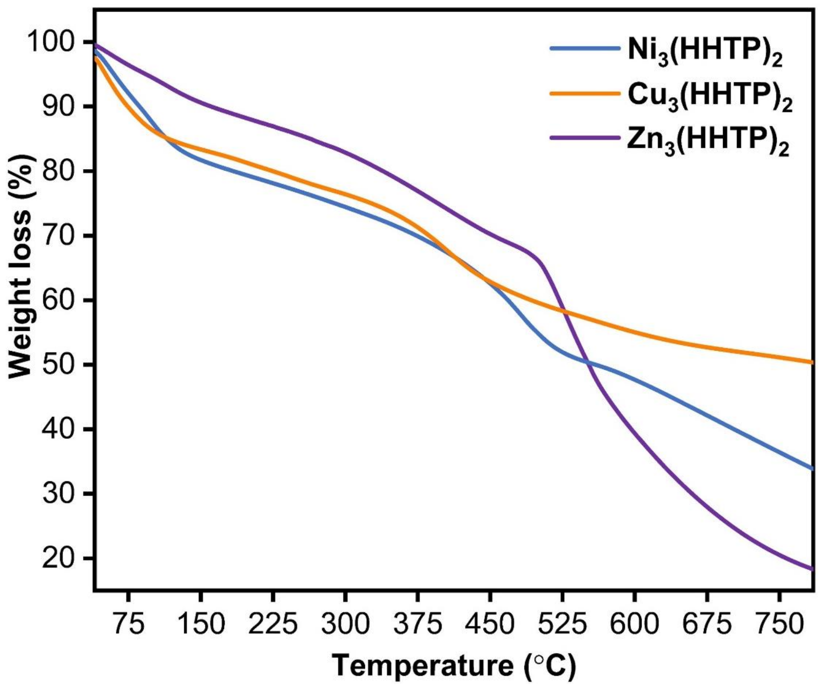

Figure S13. TGA of $M_3(HHTP)_2$ (M=Ni, Cu, Zn).

VIII. ATR-IR Spectra of MOFs

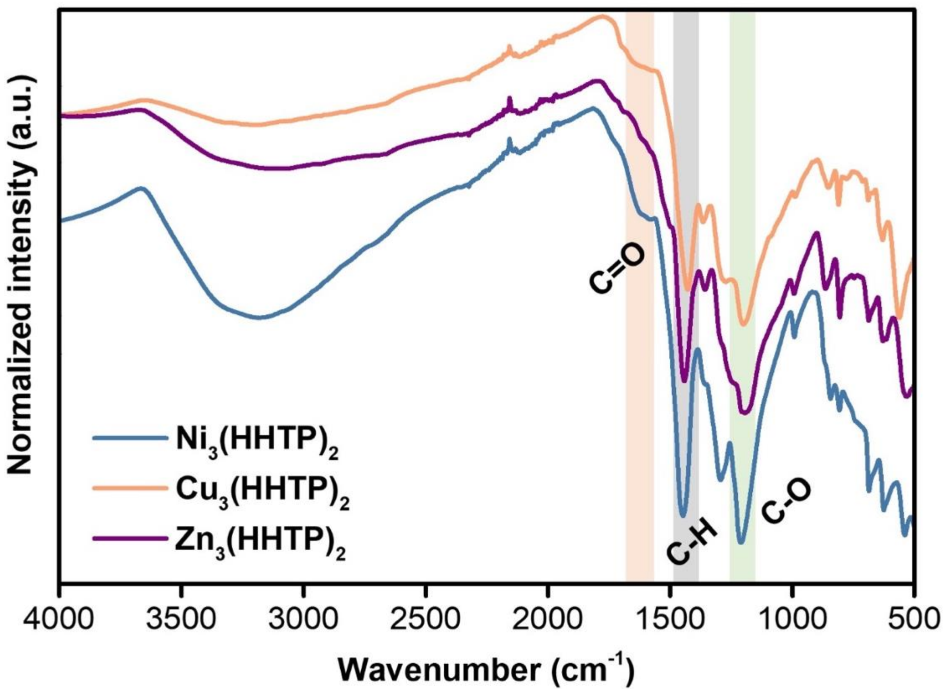

Figure S14. Comparison of ATR-IR spectra of $M_3(HHTP)_2$ (M=Ni, Cu, Zn).

IX. Conductivity Analysis of MOFs

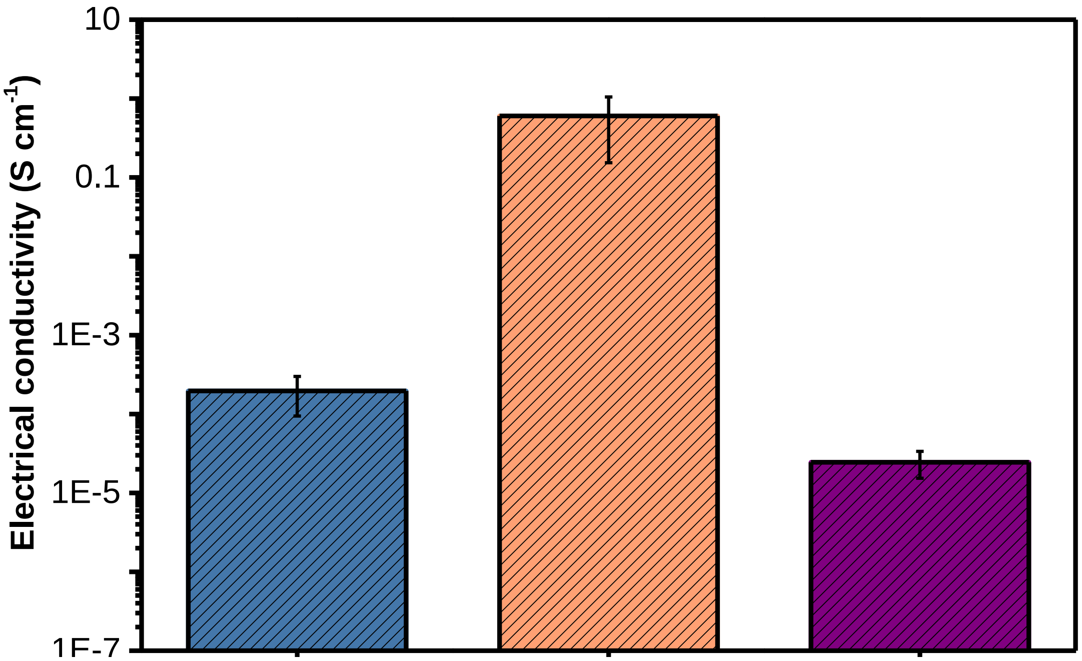

Figure S15. 4-point probe conductivity analysis of $M_3(HHTP)_2$ (M=Ni, Cu, Zn) represented by blue, orange, and purple bars, respectively.

X. Brunauer−Emmett−Teller (BET) Analysis of MOFs

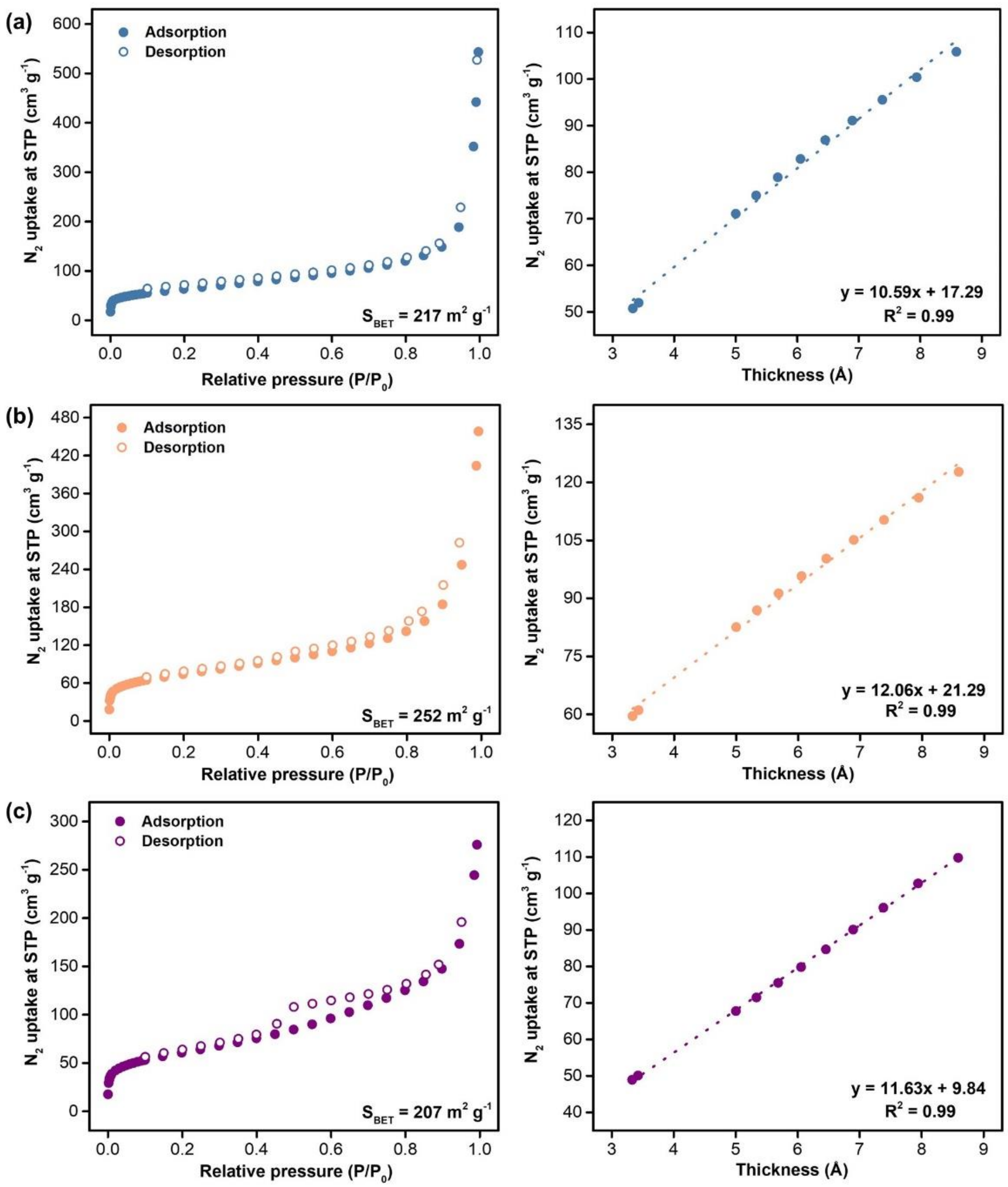

Figure S16. Nitrogen adsorption curves (left) (closed circles: adsorption) (open circles: desorption) (STP: standard temperature pressure) and *t*-plot for nitrogen adsorbed at 77 K (right) for (**a**) $Ni_3(HHTP)_2$, (**b**) $Cu_3(HHTP)_2$, and (**c**) $Zn_3(HHTP)_2$.

XI. Chemiresistive Gas Sensing

Devices were fabricated by sonicating each MOF into an aqueous suspension (concentration: 1.0 mg MOF/ 1.0 mL H_2O). 10−15 µL of MOF suspension was dropcasted onto gold interdigitated electrodes (IDE) with 10 µm interdigitation. Devices were dried in an oven set to 85°C for 10−15 minutes. Following device fabrication, there was some variability in material covering the electrode interdigitation as seen in **Figure S17**. However, despite this visual observation, sensing performance was retained across different devices and MOF synthetic batches.

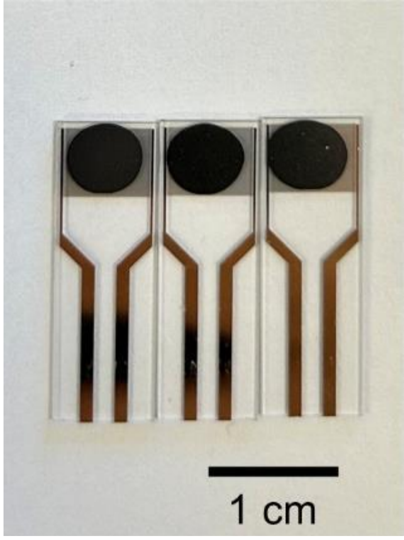

Figure S17. Image of typical MOF devices following fabrication.

Device response was calculated as normalized conductance ($-\Delta G/G_0$), where $-\Delta G$ is the change in current and G_0 is the baseline current during carrier gas equilibration.

$$\frac{-\Delta G}{G_0} = \frac{-(I_f - I_i)}{I_i} \times 100\% \quad \textbf{Eq. S1}$$

Where I_f is the final current and I_i is the initial baseline current with ampere as units.

The devices were then connected to an edge connector sealed using a custom-made Teflon chamber with inlet and outlet gas ports. Sensing performance metrics, such as magnitude of response, initial rate of response (RoR), degree or reversibility, and fidelity of metrics upon multiple cycles of single analyte exposure were determined by gas exposure experiments. For each

individual sensing experiment exposed to either a single analyte or binary mixture, at least three ($n \geq 3$) devices were used to obtain statistically significant data. The reported sensing performance metrics were calculated by (i) taking the average of the devices in each individual sensing experiment and (2) reporting the standard deviation of the value.

Devices were equilibrated in dry nitrogen (N_2) flow until a stable, relatively unchanging baseline was achieved. For the first analyte exposure to devices, sensing experiments were conducted in three stages: 1) 13 minutes in N_2 flow to establish a baseline, 2) 30 minutes of 80 ppm analyte exposure to determine resistance change as a function of analyte presence, and 3) 17 minutes recovery in N_2 flow to determine degree of device reversibility. N_2 was passed over the devices for an additional 0.5 to 2 hours to facilitate maximal recovery of the devices. Depending on the specific analyte-material pairing, some devices did not return to baseline. This initial exposure was followed by a series of consecutive analyte exposure-recovery cycles to evaluate the devices' ability to consistently detect the analyte after pre-exposure. The cycled experiments were conducted as follows: 1) 5 minutes in N_2 flow to establish a baseline, 2) 10 minutes of 80 ppm analyte exposure, 3) 15 minutes recovery in N_2 flow, and 4) repeat steps 2−3 two times.

Flow rates of dry N_2 were established with SmartTrack high flow mass flow controllers and the delivery of a specific concentration of analyte was performed using a MicroTrack low flow mass flow controller to control the flow from a 1% analyte tank with a balance of N_2. Chronoamperometry experiments were performed at a two-electrode potential of 0.1 V using a PalmSens potentiostat with series multiplexer. Each device current was sampled every 0.5 seconds.

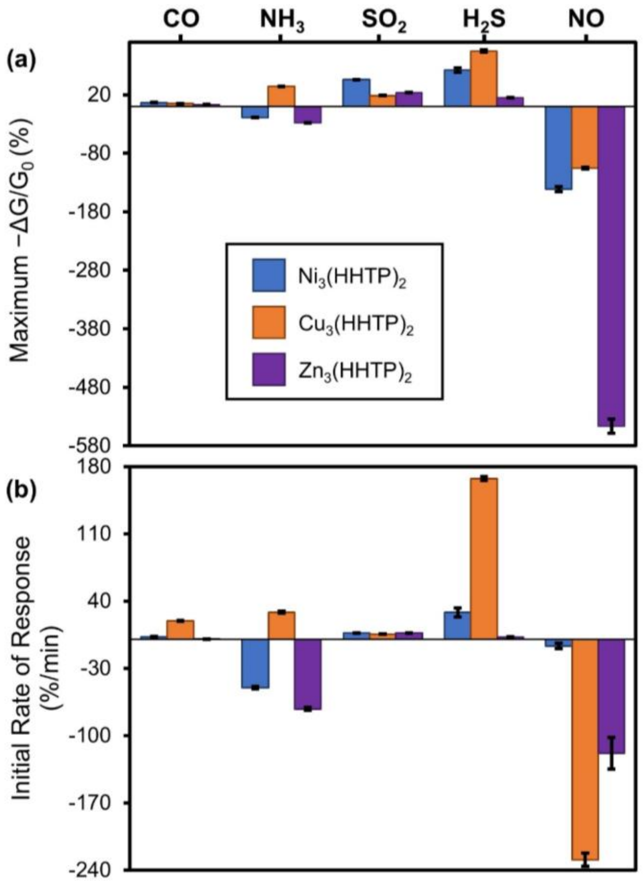

Figure S18. Features of batch 1 devices in response to the analytes. Bar graph depicts the (**a**) $-\Delta G/G_0$ during the 30-minute analyte exposure and (**b**) initial rate of response of $M_3(HHTP)_2$ (M = Ni, Cu, Zn) devices exposed to various analytes (CO, NH_3, SO_2, H_2S, and NO) at 80 ppm in dry N_2.

Table S1. Batch 1 device response and recovery time upon exposure to 80 ppm analyte.

		CO	NH_3	SO_2	H_2S	NO
$Ni_3(HHTP)_2$	Response Time (min)	11.4	0.3	21.4	18.7	18.1
	Recovery Time (min)	22.0	5.8	semi-reversible	irreversible	semi-reversible
$Cu_3(HHTP)_2$	Response Time (min)	3.0	4.0	15.7	5.0	0.7
	Recovery Time (min)	3.0	semi-reversible	semi-reversible	irreversible	semi-reversible
$Zn_3(HHTP)_2$	Response Time (min)	7.6	0.6	17.4	23.4	10.1
	Recovery Time (min)	1.4	semi-reversible	semi-reversible	semi-reversible	semi-reversible

Note: when a recovery time is denoted "semi-reversible" the devices were not able to be fully recovered but was partially recovered to varying extents.

In response to the first exposure of 80 ppm CO, $Ni_3(HHTP)_2$ yielded the highest maximum $-\Delta G/G_0$ after 30 minutes of 6.9 ± 0.6% (**Figure S20a**). Cu- and $Zn_3(HHTP)_2$ reached maximum responses of 5.7 ± 0.3% and 3.0 ± 0.7%, respectively. Following the first 30 minute exposure to CO, devices were purged with N_2 until the original baseline was reached. To test the reusability of the devices, the sensors were exposed to three subsequent CO exposures (10 min each) (**Figure S20b**). All devices retained sensitivity to CO and responses were highly reversible throughout all four consecutive exposures as seen through the metrics in **Table S2**.

As a response to 80 ppm NH_3 (**Figure S21a**), $Cu_3(HHTP)_2$ devices decreased in conductance (34.7 ± 0.6%), which aligns with the interaction expected from a p-type semiconductor exposed to a reducing gas. However, both Ni- and $Zn_3(HHTP)_2$ devices exhibited an increase of conductance of −18.7 ± 0.5%, and −28.2 ± 0.4%, respectively, likely resulting from a dehydration mechanism upon analyte exposure. For the subsequent NH_3 exposures, the direction of device response remained consistent with the first exposure (see **Figure S21b–c**, **Table S3**).

When first exposed to 80 ppm SO_2, the Ni-, Cu-, and $Zn_3(HHTP)_2$ devices exhibited maximum responses of 45.9 ± 0.4%, 18.6 ± 0.2%, and 24.0 ± 0.2%, respectively, and initial RoRs of 6.7 ± 0.2, 5.7 ± 0.1, and 6.9 ± 0.2 %/min, respectively (**Figure S22a, Table S4**). Based on all four exposures, $Ni_3(HHTP)_2$ exhibited dosimetric behavior with no reversibility following each of the four consecutive SO_2 exposures (**Figure S22a-b**). Both Cu- and $Zn_3(HHTP)_2$ devices demonstrated semi-reversibility following each of the four exposures (**Figure S22a−b**). Following each round of exposure, the subsequent exposure saw diminished metrics (magnitude of response and initial RoR) likely due to the irreversibility of analyte binding on the surface of the material.

In response to 80 ppm H_2S, all devices decrease in conductance with $Cu_3(HHTP)_2$ devices reaching 94.6 ± 1.9%, followed by $Ni_3(HHTP)_2$ (62.3 ± 3.6%), and $Zn_3(HHTP)_2$ (13.0 ± 0.4%) over the course of the 30 min first exposure (**Figure S23a**). This response is consistent with the expected interaction between a p-type semiconductor with a reducing gas as seen in previous studies.[4, 5] Both Ni- and $Cu_3(HHTP)_2$ devices functioned as dosimeters upon H_2S exposure, due to irreversible interactions between the analyte and MOFs. This irreversibility is consistent with previous results showing that H_2S oxidizes on similar materials to elemental sulfur (S^0) and sulfur-containing species.[6] Following the first exposure, both Ni- and $Cu_3(HHTP)_2$ devices exhibited a degree of response to subsequent exposures, but to a diminished extent (**Figure S23b, Table S5**). $Zn_3(HHTP)_2$ devices exhibited semi-reversibility during each recovery stage and maintained both magnitude of response and initial RoR in exposure cycles 2−4. (**Figure S23b, Table S5**). During the 10-minute exposures to H_2S, all material devices did not reach saturation.

When first exposed to 80 ppm NO over the course of a 30 min exposure, the Ni-, Cu-, and $Zn_3(HHTP)_2$ devices exhibited maximum responses of −141.4 ± 4.3%, −105.1 ± 1.5%, and −546.4 ± 12.2%, respectively (**Figure S24a**). All materials increased in conductance upon NO exposure, consistent with the expected interaction between a p-type semiconductor with an oxidizing gas. Following the initial NO exposure, the subsequent three exposures showed significant reduction in magnitude of response, likely due to saturation of the device with irreversibly bound NO or other nitrogen-containing byproducts (**Figure S24b, Table S6**).[6]

To test the reproducibility of sensing results, we repeated the four consecutive analyte exposures with devices fabricated from distinct MOF synthetic batches. The results can be found in **Figures S25−29** and **Tables S7−11**. Devices from different MOF batches retained direction of response and magnitude of sensing metrics with generally high fidelity.

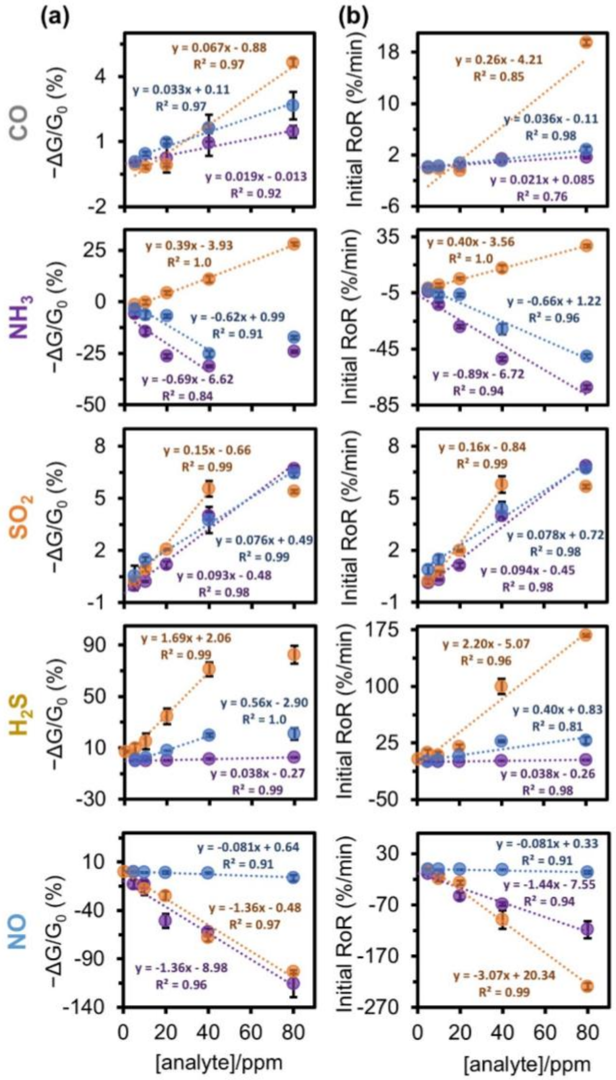

Figure S19. Linear relationships between the (**a**) maximum change in normalized conductance at 1 minute of analyte exposure and (**b**) initial rate of response during the first 0.3-1 minute of exposure of batch 1 MOF devices versus the concentration of analytes (CO, NH_3, SO_2, H_2S, and NO). $M_3(HHTP)_2$ devices (M=Ni, Cu, Zn) are represented by blue, orange, and purple, respectively. Note: inset in each figure are the linear best fit lines and R^2 values.

The maximum response at 1 minute of analyte exposure seen in **Figure S19** was selected to enable a linear fit. When modeled using the maximum response across the 30-minute analyte exposure, the response versus analyte concentration was not linear likely due to the saturation of the materials during exposure. The response at 1 minute is advantageous as it demonstrates the rapidity of the concentration dependence. It is ideal for personal protection and environmental monitoring that sensor array technologies quickly detect toxic gases and alert the user to the contaminants in their environment for the best health outcomes.

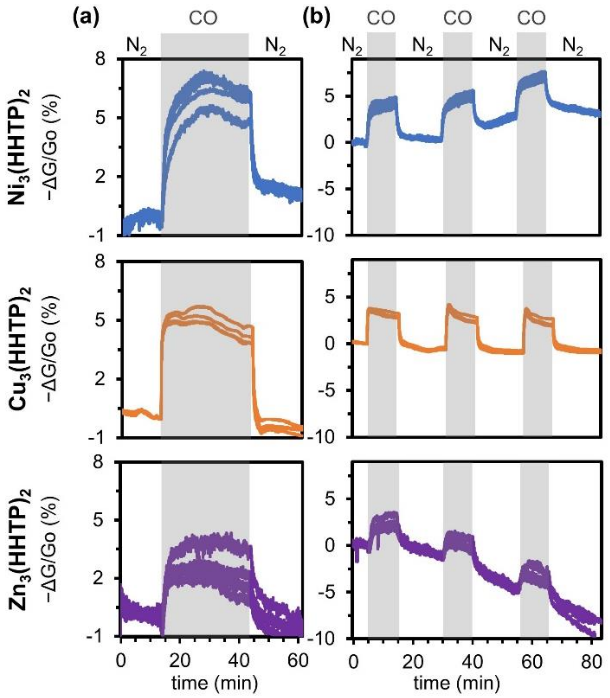

Figure S20. Chemiresistive sensing responses of batch 1 $M_3(HHTP)_2$ (M= Ni, Cu, Zn) devices toward 80 ppm CO. Responses are measured in negative normalized conductance ($-\Delta G/G_0$). (**a**) Sensing traces of MOF devices exposed to 80 ppm CO (30 mins) followed by recovery in dry N_2 flow. (**b**) Sensing traces of devices from experiment (**a**) exposed to 80 ppm CO via three cycles of analyte dosing and recovery in dry N_2 flow to test device reusability.

Table S2. Maximum response at saturation and the rate of response during the first minute of analyte exposure for batch 1 $M_3(HHTP)_2$ (M= Ni, Cu, Zn) devices exposed to 80 ppm CO over the course of four consecutive sensing exposures.

		**$Ni_3(HHTP)_2$**	**$Cu_3(HHTP)_2$**	**$Zn_3(HHTP)_2$**
**Exposure #1**	$-\Delta G/G_0$ (%)	6.9 ± 0.6	5.2 ± 0.3	3.0 ± 0.7
	Initial RoR (%/min)	2.7 ± 0.6	19.5 ± 0.5	1.5 ± 0.3
**Exposure #2**	$-\Delta G/G_0$ (%)	4.4 ± 0.4	3.6 ± 0.1	2.6 ± 0.6
	Initial RoR (%/min)	3.3 ± 0.4	10.8 ± 1.1	1.7 ± 0.3
**Exposure #3**	$-\Delta G/G_0$ (%)	4.7 ± 0.3	4.5 ± 0.1	1.9 ± 0.6
	Initial RoR (%/min)	3.4 ± 0.2	5.7 ± 0.3	1.5 ± 0.3
**Exposure #4**	$-\Delta G/G_0$ (%)	4.4 ± 0.3	4.3 ± 0.1	2.0 ± 0.6
	Initial RoR (%/min)	3.0 ± 0.2	6.2 ± 0.3	1.3 ± 0.1

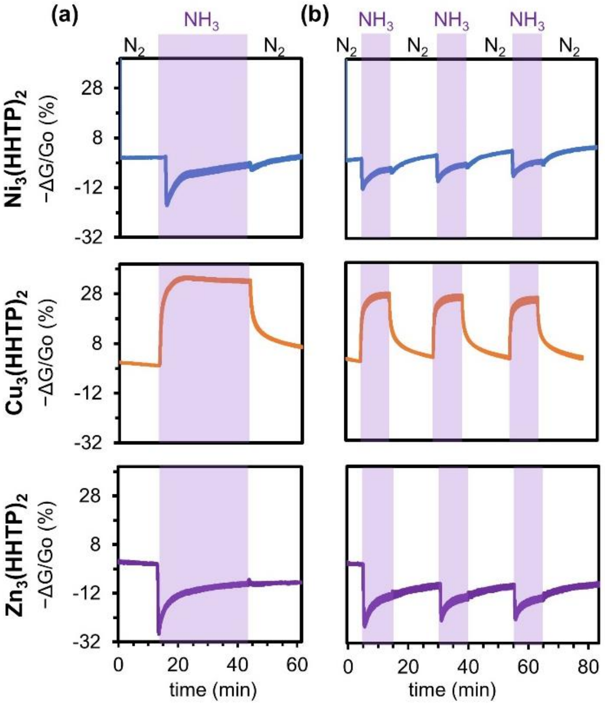

Figure S21. Chemiresistive sensing responses of batch 1 $M_3(HHTP)_2$ (M= Ni, Cu, Zn) devices toward 80 ppm NH_3. Responses are measured in negative normalized conductance ($-\Delta G/G_0$). (**a**) Sensing traces of MOF devices exposed to 80 ppm NH_3 (30 mins) followed by recovery in dry N_2 flow. (**b**) Sensing traces of devices from experiments (**a**) exposed to 80 ppm NH_3 via three cycles of analyte dosing and recovery in dry N_2 flow to test device reusability.

Table S3. Maximum response at saturation and the rate of response during the first minute of analyte exposure for batch 1 $M_3(HHTP)_2$ (M= Ni, Cu, Zn) devices exposed to 80 ppm NH_3 over the course of five consecutive sensing exposures.

		$Ni_3(HHTP)_2$	$Cu_3(HHTP)_2$	$Zn_3(HHTP)_2$
**Exposure #1**	$-\Delta G/G_0$ (%)	−18.7 ± 0.5	34.7 ± 0.6	−28.2 ± 0.4
	Initial RoR (%/min)	−50.2 ± 1.4	28.5 ± 1.1	−72.3 ± 1.5
**Exposure #2**	$-\Delta G/G_0$ (%)	−11.5 ± 0.6	27.1 ± 0.7	−24.4 ± 1.0
	Initial RoR (%/min)	−39.7 ± 1.0	39.6 ± 2.8	−63.9 ± 2.2
**Exposure #3**	$-\Delta G/G_0$ (%)	−10.1 ± 0.4	24.2 ± 0.5	−11.9 ± 1.7
	Initial RoR (%/min)	−47.2 ± 2.5	46.4 ± 2.6	−35.6 ± 1.6
**Exposure #4**	$-\Delta G/G_0$ (%)	−9.7 ± 0.5	23.5 ± 0.6	−12.4 ± 0.5
	Initial RoR (%/min)	−48.8 ± 2.2	39.8 ± 1.4	−39.7 ± 1.5

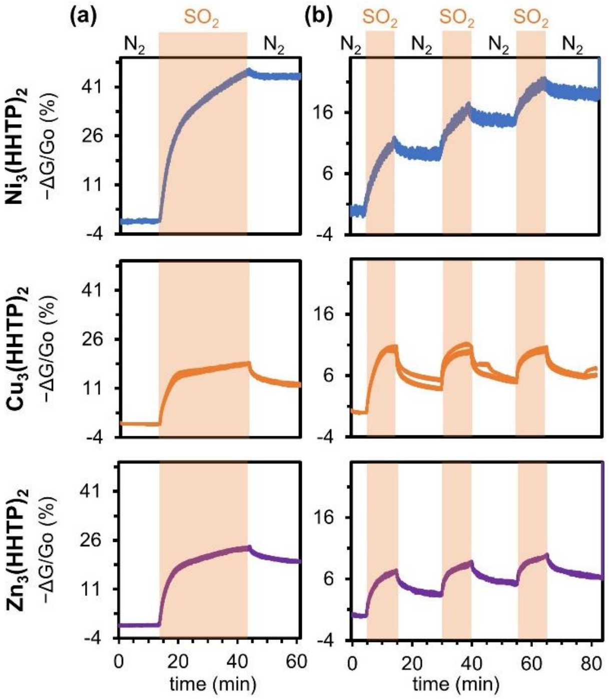

Figure S22. Chemiresistive sensing responses of batch 1 $M_3(HHTP)_2$ (M= Ni, Cu, Zn) devices toward 80 ppm SO_2. Responses are measured in negative normalized conductance ($-\Delta G/G_0$). (**a**) Sensing traces of MOF devices exposed to 80 ppm SO_2 (30 mins) followed by recovery in dry N_2 flow. (**b**) Sensing traces of devices from experiment (**a**) exposed to 80 ppm SO_2 via three cycles of analyte dosing and recovery in dry N_2 flow to test device reusability.

Table S4. Maximum response at saturation and the rate of response during the first minute of analyte exposure for batch 1 $M_3(HHTP)_2$ (M= Ni, Cu, Zn) devices exposed to 80 ppm SO_2 over the course of four consecutive sensing exposures.

		$Ni_3(HHTP)_2$	$Cu_3(HHTP)_2$	$Zn_3(HHTP)_2$
**Exposure #1**	$-\Delta G/G_0$ (%)	45.9 ± 0.4	18.6 ± 0.2	24.0 ± 0.2
	Initial RoR (%/min)	6.7 ± 0.2	5.7 ± 0.1	6.9 ± 0.2
**Exposure #2**	$-\Delta G/G_0$ (%)	11.6 ± 0.3	10.5 ± 0.3	7.3 ± 0.1
	Initial RoR (%/min)	2.7 ± 0.7	3.8 ± 0.1	3.0 ± 0.1
**Exposure #3**	$-\Delta G/G_0$ (%)	7.6 ± 0.4	6.0 ± 0.1	5.1 ± 0.1
	Initial RoR (%/min)	2.3 ± 0.1	3.0 ± 0.1	2.4 ± 0.1
**Exposure #4**	$-\Delta G/G_0$ (%)	6.7 ± 0.3	5.1 ± 0.1	4.6 ± 0.1
	Initial RoR (%/min)	2.0 ± 0.1	1.9 ± 0.1	2.0 ± 0.1

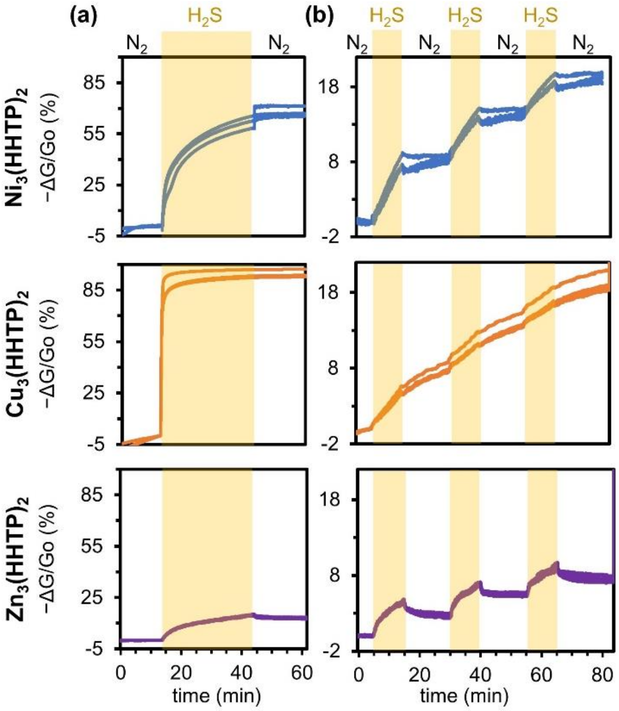

Figure S23. Chemiresistive sensing responses of batch 1 $M_3(HHTP)_2$ (M= Ni, Cu, Zn) devices toward 80 ppm H_2S. Responses are measured in negative normalized conductance ($-\Delta G/G_0$). (**a**) Sensing traces of MOF devices exposed to 80 ppm H_2S (30 mins) followed by recovery in dry N_2 flow. (**b**) Sensing traces of devices from experiment (**a**) exposed to 80 ppm H_2S via three cycles of analyte dosing and recovery in dry N_2 flow to test device reusability.

Table S5. Maximum response at saturation and the rate of response during the first minute of analyte exposure for batch 1 $M_3(HHTP)_2$ (M= Ni, Cu, Zn) devices exposed to 80 ppm H_2S over the course of four consecutive sensing exposures.

		$Ni_3(HHTP)_2$	$Cu_3(HHTP)_2$	$Zn_3(HHTP)_2$
**Exposure #1**	$-\Delta G/G_0$ (%)	62.3 ± 3.6	94.6 ± 1.9	15.0 ± 0.4
	Initial RoR (%/min)	28.2 ± 4.7	167.4 ± 2.0	2.6 ± 0.1
**Exposure #2**	$-\Delta G/G_0$ (%)	8.1 ± 0.9	5.1 ± 0.4	4.7 ± 0.1
	Initial RoR (%/min)	0.7 ± 0.3	0.8 ± 0.1	1.4 ± 0.1
**Exposure #3**	$-\Delta G/G_0$ (%)	6.0 ± 0.2	3.7 ± 0.2	4.3 ± 0.1
	Initial RoR (%/min)	0.7 ± 0.1	0.8 ± 0.1	1.3 ± 0.1
**Exposure #4**	$-\Delta G/G_0$ (%)	4.4 ± 0.5	3.1 ± 0.1	3.8 ± 0.2
	Initial RoR (%/min)	0.4 ± 0.2	0.6 ± 0.1	1.5 ± 0.3

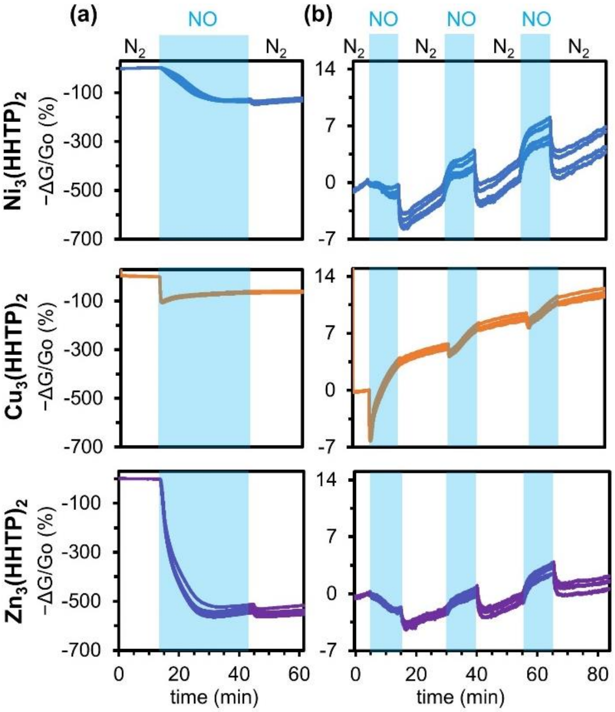

Figure S24. Chemiresistive sensing responses of batch 1 $M_3(HHTP)_2$ (M= Ni, Cu, Zn) devices toward 80 ppm NO. Responses are measured in negative normalized conductance ($-\Delta G/G_0$). (**a**) Sensing traces of MOF devices exposed to 80 ppm NO (30 mins) followed by recovery in dry N_2 flow. (**b**) Sensing traces of devices from experiment (**a**) exposed to 80 ppm NO via three cycles of analyte dosing and recovery in dry N_2 flow to test device reusability.

Table S6. Maximum response at saturation and the rate of response during the first minute of analyte exposure for batch 1 $M_3(HHTP)_2$ (M= Ni, Cu, Zn) devices exposed to 80 ppm NO over the course of four consecutive sensing exposures.

		$Ni_3(HHTP)_2$	$Cu_3(HHTP)_2$	$Zn_3(HHTP)_2$
**Exposure #1**	$-\Delta G/G_0$ (%)	−141.4 ± 4.3	−105.1 ± 1.5	−546.4 ± 12.2
	Initial RoR (%/min)	−6.7 ± 3.0	−229.1 ± 6.8	−118.3 ± 16.5
**Exposure #2**	$-\Delta G/G_0$ (%)	−1.5 ± 0.5	−5.7 ± 0.4	−2.2 ± 0.2
	Initial RoR (%/min)	−0.2 ± 0.2	−13.4 ± 1.5	−0.3 ± 0.1
**Exposure #3**	$-\Delta G/G_0$ (%)	3.8 ± 0.2	−0.9 ± 0.1	2.0 ± 1.1
	Initial RoR (%/min)	1.3 ± 0.1	−2.1 ± 0.5	0.5 ± 0.1
**Exposure #4**	$-\Delta G/G_0$ (%)	4.9 ± 0.1	−0.9 ± 0.2	3.6 ± 0.2
	Initial RoR (%/min)	1.8 ± 0.1	−1.0 ± 0.2	1.0 ± 0.1

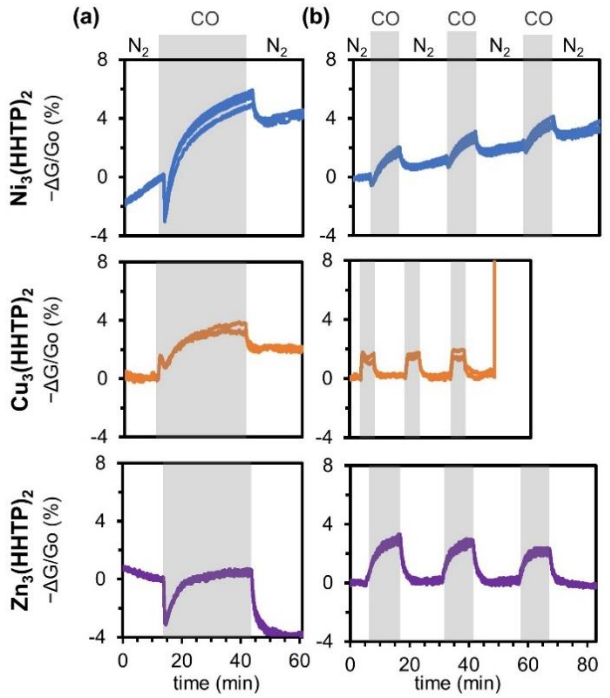

Figure S25. Chemiresistive sensing responses of batch 2 $M_3(HHTP)_2$ (M= Ni, Cu, Zn) toward 80 ppm CO. Responses are measured in negative normalized conductance ($-\Delta G/G_0$). (**a**) Sensing traces of MOF devices exposed to 80 ppm CO (30 mins) followed by recovery in dry N_2 flow. (**b**) Sensing traces of devices from experiment (**a**) exposed to 80 ppm CO via three cycles of analyte dosing and recovery in dry N_2 flow to test device reusability. (Note: for $Cu_3(HHTP)_2$ the 3x (exposure-recovery) cycle was performed using 5 minutes N_2 flow, 5 minutes of CO exposure, and 10 minutes recovery in N_2 flow.)

Table S7. Maximum response at saturation and the rate of response during the first minute of analyte exposure for batch 2 $M_3(HHTP)_2$ (M=Ni, Cu, Zn) devices exposed to 80 ppm CO over the course of four consecutive sensing exposures.

		$Ni_3(HHTP)_2$	$Cu_3(HHTP)_2$	$Zn_3(HHTP)_2$
**Exposure #1**	$-\Delta G/G_0$ (%)	5.6 ± 0.3	3.7 ± 0.3	0.9 ± 0.1
	Initial RoR (%/min)	−7.0 ± 0.3	2.6 ± 0.1	−6.3 ± 0.3
**Exposure #2**	$-\Delta G/G_0$ (%)	1.9 ± 0.2	1.7 ± 0.1	3.0 ± 0.2
	Initial RoR (%/min)	−1.0 ± 0.3	2.9 ± 0.1	0.6 ± 0.1
**Exposure #3**	$-\Delta G/G_0$ (%)	1.8 ± 0.1	1.7 ± 0.1	2.8 ± 0.2
	Initial RoR (%/min)	−0.5 ± 0.2	1.6 ± 0.1	1.0 ± 0.1
**Exposure #4**	$-\Delta G/G_0$ (%)	1.7 ± 0.2	1.8 ± 0.2	2.2 ± 0.1
	Initial RoR (%/min)	−0.6 ± 0.4	1.8 ± 0.1	0.8 ± 0.1

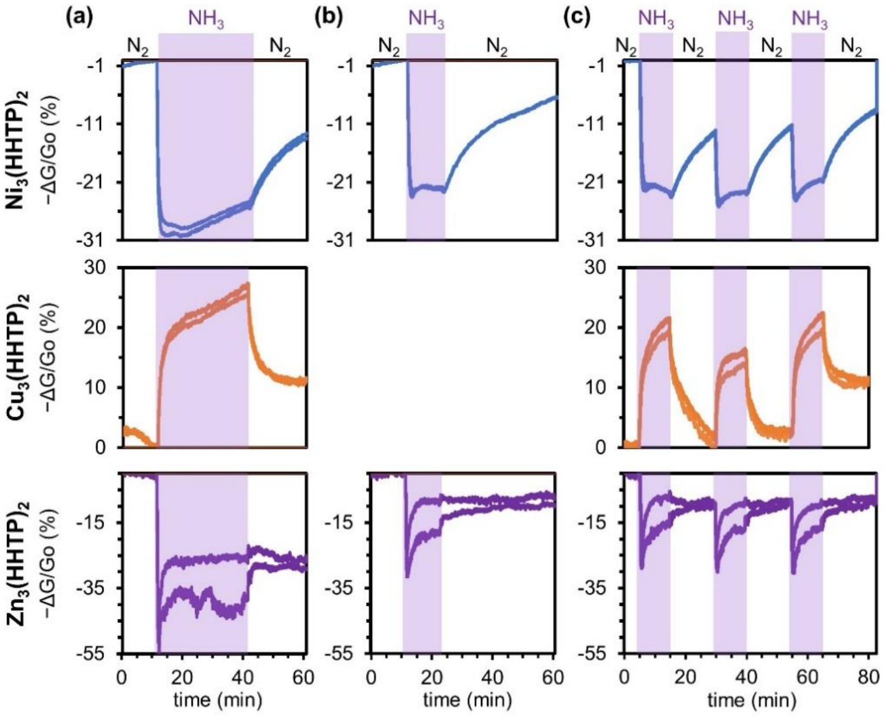

Figure S26. Chemiresistive sensing responses of batch 2 $M_3(HHTP)_2$ (M= Ni, Cu, Zn) toward 80 ppm NH_3. Responses are measured in negative normalized conductance ($-\Delta G/G_0$). (**a**) Sensing traces of MOF devices exposed to 80 ppm NH_3 (30 mins) followed by recovery in dry N_2 flow. (**b**) Sensing traces of devices from experiments (**a**) exposed to 80 ppm NH_3 to test device reusability. (**c**) Sensing traces of devices from experiments (**a-b**) exposed to 80 ppm NH_3 via three cycles of analyte dosing and recovery in dry N_2 flow. (Note: for $Cu_3(HHTP)_2$ devices, only four exposures were performed as opposed to five exposures for the Ni- and $Zn_3(HHTP)_2$ devices.)

Table S8. Maximum response at saturation and the rate of response during the first minute of analyte exposure for batch 2 $M_3(HHTP)_2$ (M=Ni, Cu, Zn) devices exposed to 80 ppm NH_3 over the course of five consecutive sensing exposures. (Note: for $Cu_3(HHTP)_2$ devices, only four exposures were performed.)

		$Ni_3(HHTP)_2$	$Cu_3(HHTP)_2$	$Zn_3(HHTP)_2$
**Exposure #1**	$-\Delta G/G_0$ (%)	−29.7 ± 0.7	26.5 ± 0.9	−53.0 ± 1.4
	Initial RoR (%/min)	−44.4 ± 3.2	20.4 ± 0.9	−99.8 ± 9.5
**Exposure #2**	$-\Delta G/G_0$ (%)	−23.2 ± 0.4	20.6 ± 1.0	−29.2 ± 2.5
	Initial RoR (%/min)	−37.5 ± 3.1	12.1 ± 0.6	−75.2 ± 0.9
**Exposure #3**	$-\Delta G/G_0$ (%)	−23.5 ± 0.1	15.3 ± 1.2	−27.8 ± 1.1
	Initial RoR (%/min)	−35.2 ± 2.4	9.4 ± 0.2	−92.5 ± 1.1
**Exposure #4**	$-\Delta G/G_0$ (%)	−24.7 ± 0.5	21.2 ± 1.3	−26.6 ± 3.5
	Initial RoR (%/min)	−23.1 ± 3.1	10.3 ± 0.4	−65.1 ± 10.1
**Exposure #5**	$-\Delta G/G_0$ (%)	−23.8 ± 0.4	N/A	−26.9 ± 3.5
	Initial RoR (%/min)	−21.8 ± 1.3	N/A	−39.5 ± 7.9

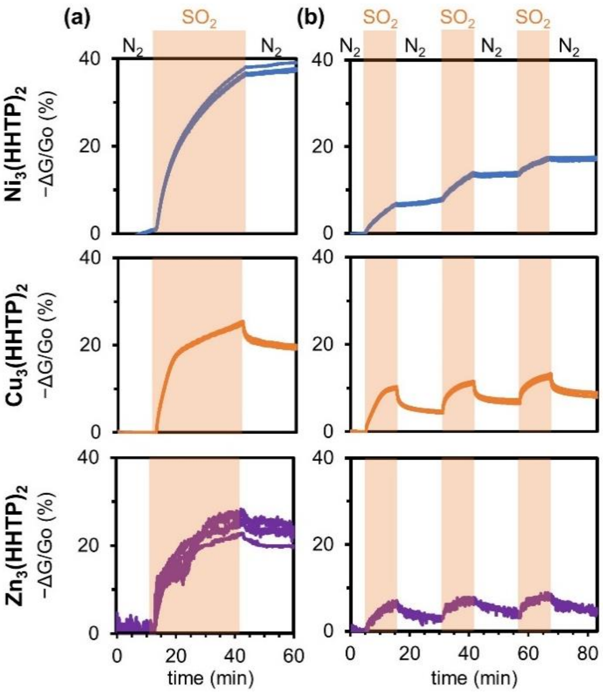

Figure S27. Chemiresistive sensing responses of batch 2 $M_3(HHTP)_2$ (M= Ni, Cu, Zn) toward 80 ppm SO_2. Responses are measured in negative normalized conductance ($-\Delta G/G_0$). (**a**) Sensing traces of MOF devices exposed to 80 ppm SO_2 (30 mins) followed by recovery in dry N_2 flow. (**b**) Sensing traces of devices from experiment (**a**) exposed to 80 ppm SO_2 via three cycles of analyte dosing and recovery in dry N_2 flow to test device reusability.

Table S9. Maximum response at saturation and the rate of response during the first minute of analyte exposure for batch 2 $M_3(HHTP)_2$ (M=Ni, Cu, Zn) devices exposed to 80 ppm SO_2 over the course of four consecutive sensing exposures.

		$Ni_3(HHTP)_2$	$Cu_3(HHTP)_2$	$Zn_3(HHTP)_2$
**Exposure #1**	$-\Delta G/G_0$ (%)	36.9 ± 0.7	25.2 ± 0.3	25.7 ± 2.3
	Initial RoR (%/min)	4.2 ± 0.1	4.4 ± 0.1	6.6 ± 2.2
**Exposure #2**	$-\Delta G/G_0$ (%)	6.8 ± 0.1	10.2 ± 0.2	7.0 ± 0.1
	Initial RoR (%/min)	1.3 ± 0.1	2.3 ± 0.1	2.0 ± 0.0
**Exposure #3**	$-\Delta G/G_0$ (%)	6.00 ± 0.1	6.1 ± 0.1	4.3 ± 0.1
	Initial RoR (%/min)	1.1 ± 0.1	2.4 ± 0.1	1.9 ± 0.2
**Exposure #4**	$-\Delta G/G_0$ (%)	3.6 ± 0.1	5.4 ± 0.1	4.8 ± 0.2
	Initial RoR (%/min)	0.8 ± 0.1	2.0 ± 0.1	1.8 ± 0.2

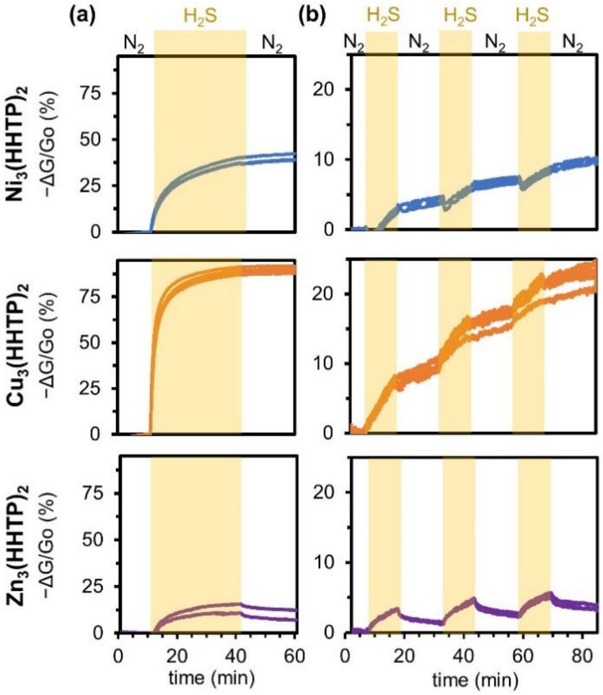

Figure S28. Chemiresistive sensing responses of batch 2 $M_3(HHTP)_2$ (M= Ni, Cu, Zn) toward 80 ppm H_2S. Responses are measured in negative normalized conductance ($-\Delta G/G_0$). (**a**) Sensing traces of MOF devices exposed to 80 ppm H_2S (30 mins) followed by recovery in dry N_2 flow. (**b**) Sensing traces of devices from experiment (**a**) exposed to 80 ppm H_2S via three cycles of analyte dosing and recovery in dry N_2 flow to test device reusability.

Table S10. Maximum response at saturation and the rate of response during the first minute of analyte exposure for batch 2 $M_3(HHTP)_2$ (M=Ni, Cu, Zn) devices exposed to 80 ppm H_2S over the course of four consecutive sensing exposures.

		$Ni_3(HHTP)_2$	$Cu_3(HHTP)_2$	$Zn_3(HHTP)_2$
**Exposure #1**	$-\Delta G/G_0$ (%)	39.1 ± 2.5	89.7 ± 1.7	13.4 ± 2.3
	Initial RoR (%/min)	7.7 ± 1.0	41.8 ± 4.6	2.6 ± 0.3
**Exposure #2**	$-\Delta G/G_0$ (%)	3.3 ± 0.3	7.5 ± 0.4	3.4 ± 0.1
	Initial RoR (%/min)	−1.7 ± 0.2	1.3 ± 0.1	0.4 ± 0.1
**Exposure #3**	$-\Delta G/G_0$ (%)	2.5 ± 0.2	6.0 ± 0.6	3.3 ± 0.3
	Initial RoR (%/min)	-0.8 ± 0.2	1.3 ± 0.4	0.7 ± 0.1
**Exposure #4**	$-\Delta G/G_0$ (%)	1.9 ± 0.3	4.2 ± 0.2	2.8 ± 0.1
	Initial RoR (%/min)	−0.7 ± 0.0	1.0 ± 0.2	0.6 ± 0.1

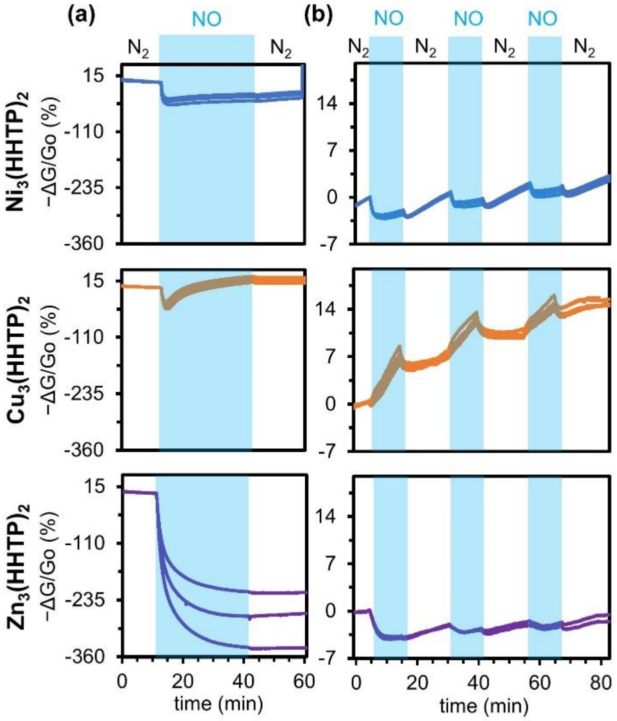

Figure S29. Chemiresistive sensing responses of batch 2 $M_3(HHTP)_2$ (M= Ni, Cu, Zn) toward 80 ppm NO. Responses are measured in negative normalized conductance ($-\Delta G/G_0$). (**a**) Sensing traces of MOF devices exposed to 80 ppm NO (30 mins) followed by recovery in dry N_2 flow. (**b**) Sensing traces of devices from experiment (a) exposed to 80 ppm NO via three cycles of analyte dosing and recovery in dry N_2 flow to test device reusability.

Table S11. Maximum response at saturation and the rate of response during the first minute of analyte exposure for batch 2 $M_3(HHTP)_2$ (M=Ni, Cu, Zn) devices exposed to 80 ppm NO over the course of four consecutive sensing exposures.

		$Ni_3(HHTP)_2$	$Cu_3(HHTP)_2$	$Zn_3(HHTP)_2$
**Exposure #1**	$-\Delta G/G_0$ (%)	−42.1 ± 5.0	−40.5 ± 6.3	−279.5 ± 50.1
	Initial RoR (%/min)	−30.8 ± 3.7	−33.7 ± 3.9	−96.5 ± 15.9
**Exposure #2**	$-\Delta G/G_0$ (%)	−2.9 ± 0.2	7.0 ± 0.9	−4.2 ± 0.2
	Initial RoR (%/min)	−2.2 ± 0.1	0.6 ± 0.4	−1.5 ± 0.1
**Exposure #3**	$-\Delta G/G_0$ (%)	−1.9 ± 0.1	5.5 ± 0.6	−1.2 ± 0.1
	Initial RoR (%/min)	−1.5 ± 0.1	0.8 ± 0.4	−0.5 ± 0.1
**Exposure #4**	$-\Delta G/G_0$ (%)	−1.4 ± 0.1	4.5 ± 0.8	−0.9 ± 0.2
	Initial RoR (%/min)	−1.2 ± 0.1	0.8 ± 0.2	−0.2 ± 0.1

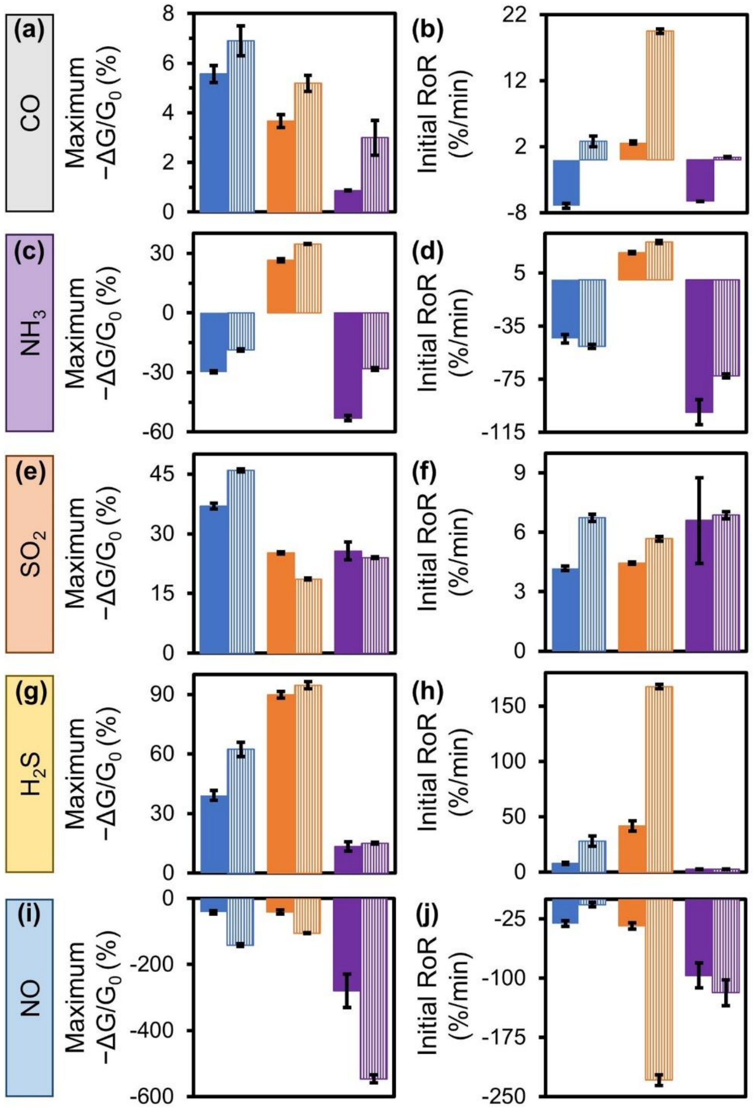

Figure S30. Bar graphs comparing (**a**, **c**, **e**, **g**, **i**) maximum response during 30-minute analyte exposure and (**b**, **d**, **f**, **h**, **j**) initial RoR of two batches of $M_3(HHTP)_2$ upon first exposure to various analytes. (M=Ni (blue), Cu (orange), Zn (purple)). Devices are exposed to 80 ppm analyte (CO, NH_3, SO_2, H_2S, or NO) over a 30-minute exposure. Batch two and one are represented by solid and striped bars, respectively.

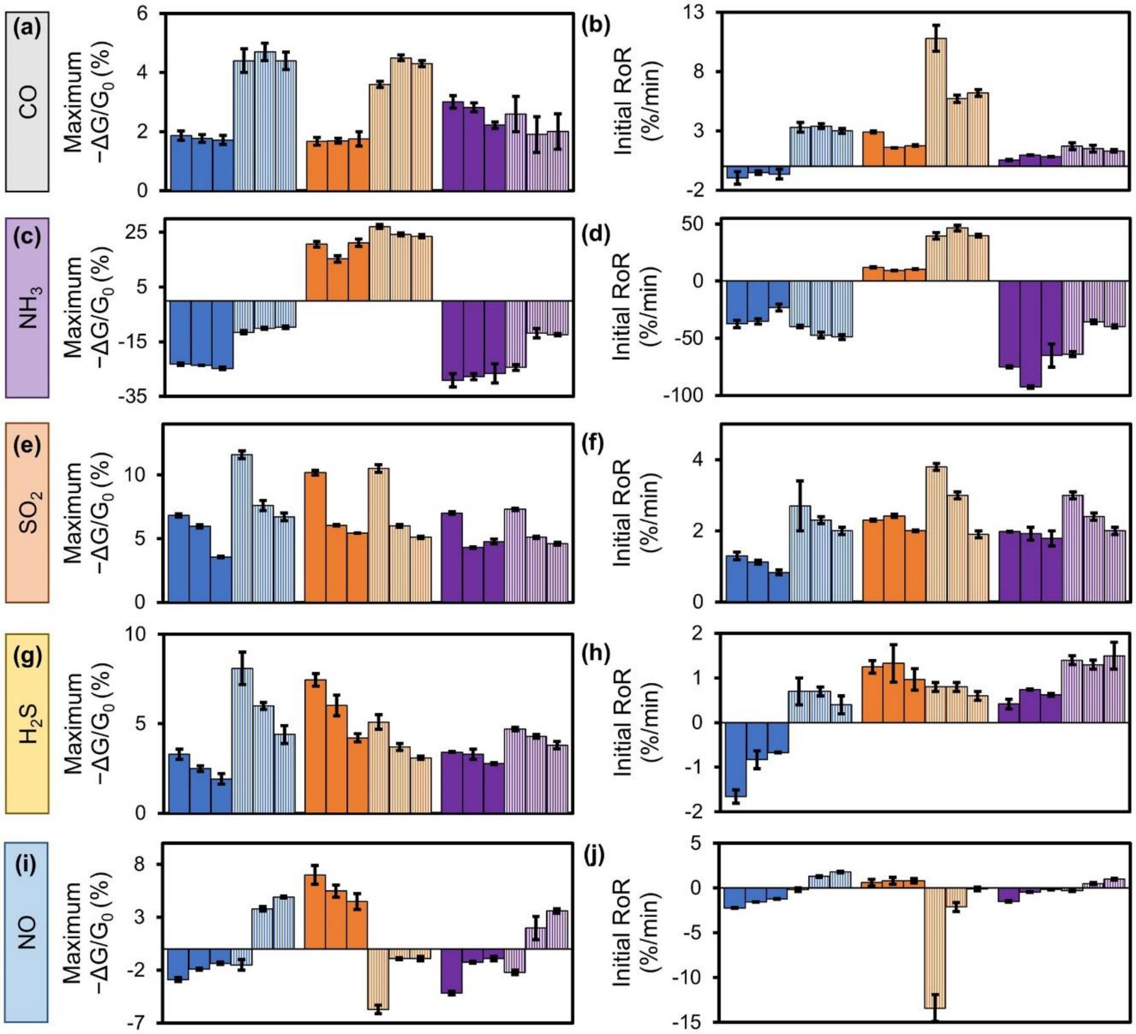

Figure S31. Bar graphs comparing (**a**, **c**, **e**, **g**, **i**) maximum response during 10-minute analyte exposure and (**b**, **d**, **f**, **h**, **j**) initial RoR of two batches of $M_3(HHTP)_2$ upon second, third, and fourth exposure to various analytes. (M=Ni (blue), Cu (orange), Zn (purple)). Devices are exposed to 80 ppm analyte (CO, NH_3, SO_2, H_2S, or NO) over a 10-minute exposure followed by a 15-minute recovery in N_2. This exposure/recovery process was repeated twice. Batch two and one are represented by solid and striped bars, respectively.

SO_2 & H_2S Mixtures Sensing Traces

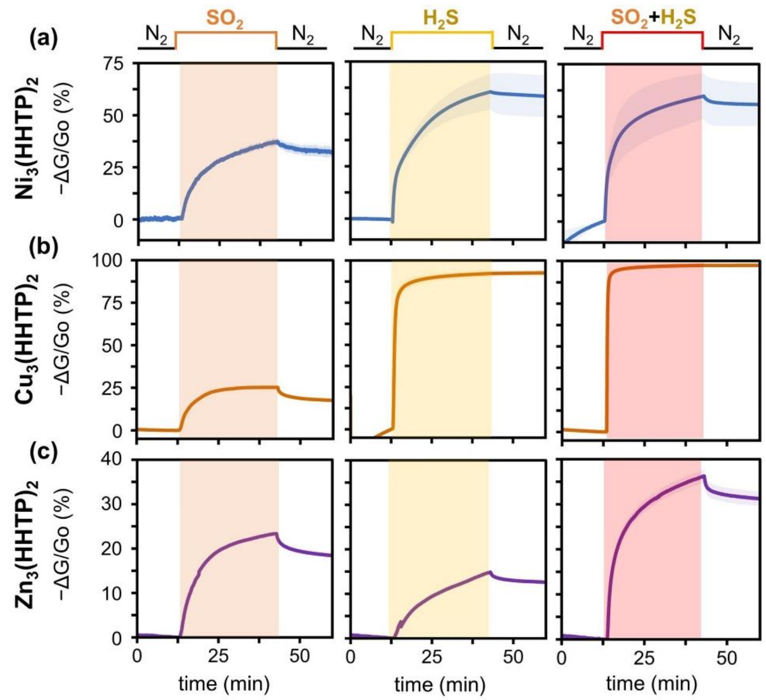

Figure S32. Chemiresistive sensing responses of (**a**) $Ni_3(HHTP)_2$, (**b**) $Cu_3(HHTP)_2$, (**c**) $Zn_3(HHTP)_2$ towards individual exposures of 40 ppm SO_2 and 40 ppm H_2S alongside simultaneous dual exposure of 40 ppm SO_2 and 40 ppm H_2S.

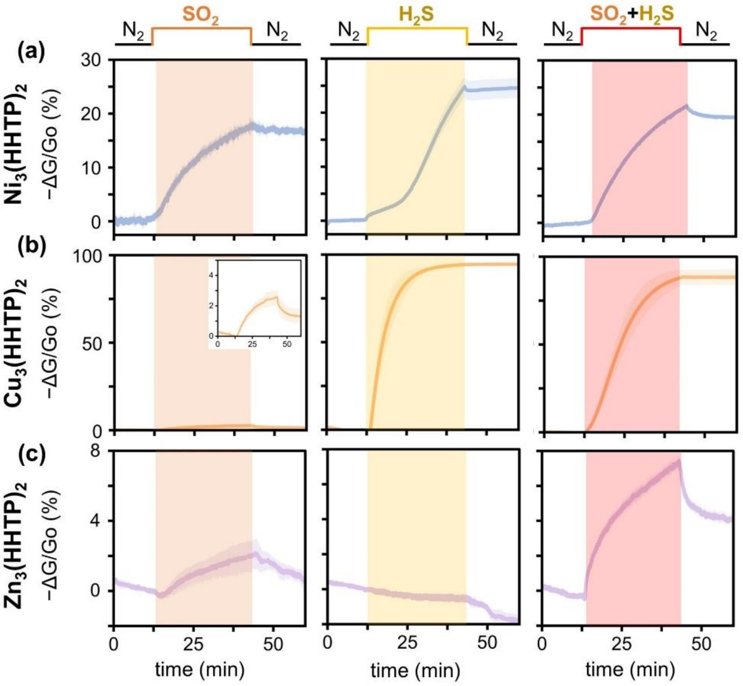

Figure S33. Chemiresistive sensing responses of (**a**) $Ni_3(HHTP)_2$, (**b**) $Cu_3(HHTP)_2$, (**c**) $Zn_3(HHTP)_2$ towards simultaneous exposure of 5 ppm SO_2 and 5 ppm H_2S alongside traces in which devices are exposed to individual SO_2 and H_2S doses at 5 ppm. Note: Inset shows a zoomed in response of analyte exposure.

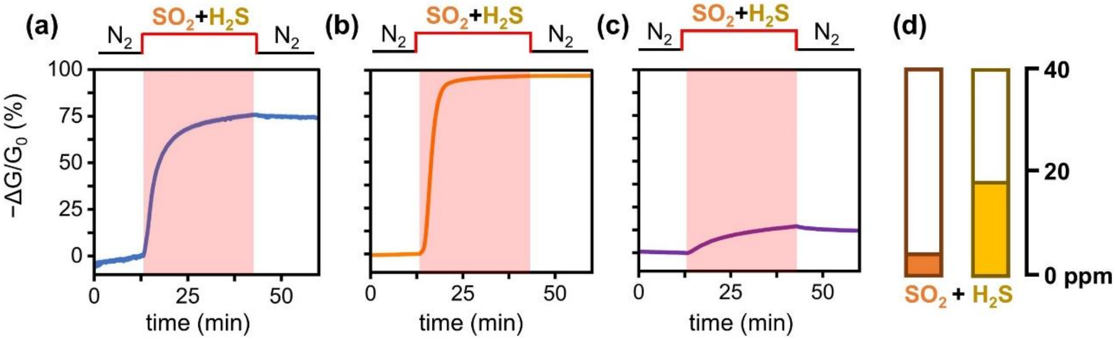

Figure S34. Sensing traces of (**a**) $Ni_3(HHTP)_2$ (**b**) $Cu_3(HHTP)_2$ and (**c**) $Zn_3(HHTP)_2$ devices exposed to a mixture of 4 ppm SO_2 and 18 ppm H_2S. (**d**) Plot showing the composition and concentration of the mixture.

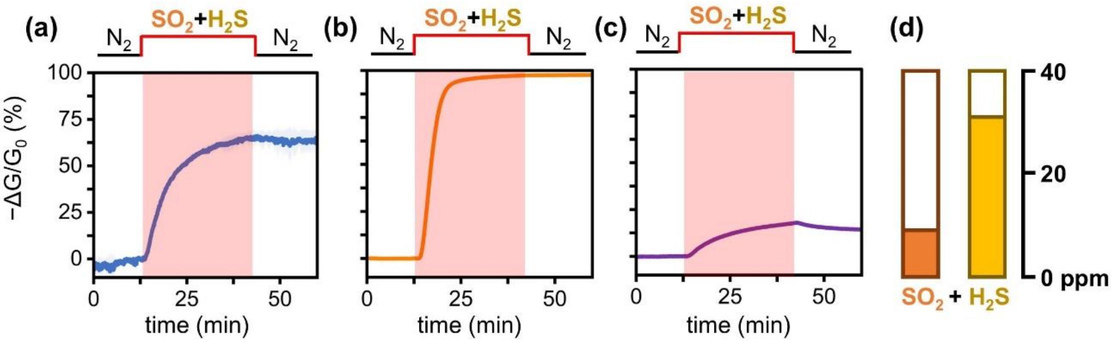

Figure S35. Sensing traces of (**a**) $Ni_3(HHTP)_2$, (**b**) $Cu_3(HHTP)_2$, and (**c**) $Zn_3(HHTP)_2$ devices exposed to a mixture of 9 ppm SO_2 and 31 ppm H_2S. (**d**) Plot showing the composition and concentration of the mixture.

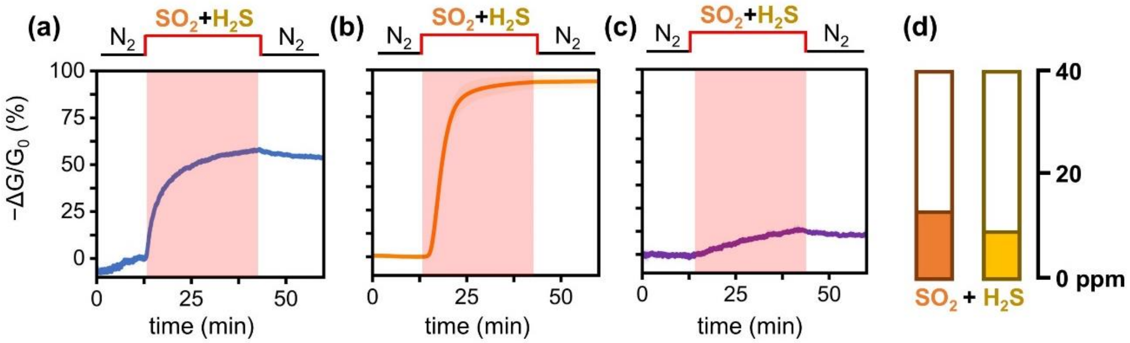

Figure S36. Sensing traces of (**a**) $Ni_3(HHTP)_2$, (**b**) $Cu_3(HHTP)_2$, and (**c**) $Zn_3(HHTP)_2$ devices exposed to a mixture of 13 ppm SO_2 and 9 ppm H_2S. (**d**) Plot showing the composition and concentration of the mixture.

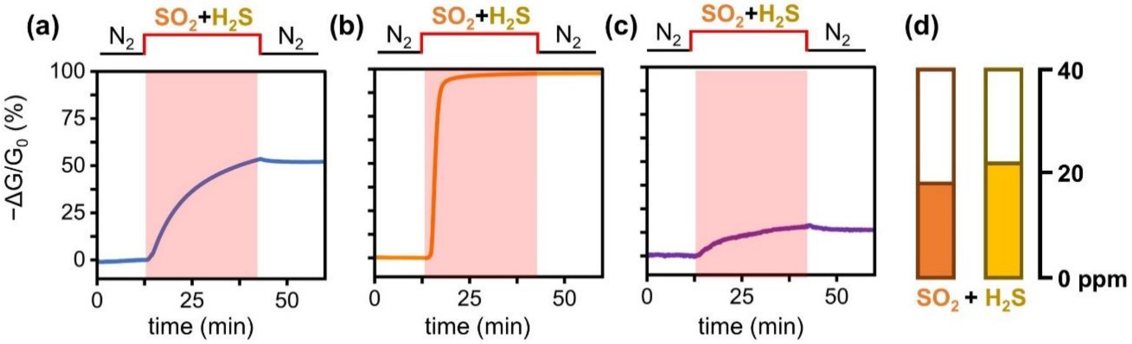

Figure S37. Sensing traces of (**a**) $Ni_3(HHTP)_2$, (**b**) $Cu_3(HHTP)_2$, and (**c**) $Zn_3(HHTP)_2$ devices exposed to a mixture of 18 ppm SO_2 and 22 ppm H_2S. (**d**) Plot showing the composition and concentration of the mixture.

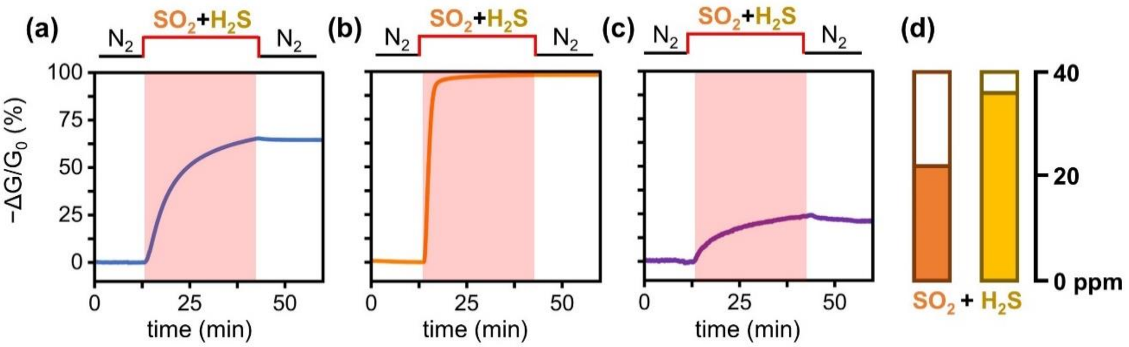

Figure S38. Sensing traces of (**a**) $Ni_3(HHTP)_2$, (**b**) $Cu_3(HHTP)_2$, and (**c**) $Zn_3(HHTP)_2$ devices exposed to a mixture of 22 ppm SO_2 and 36 ppm H_2S. (**d**) Plot showing the composition and concentration of the mixture.

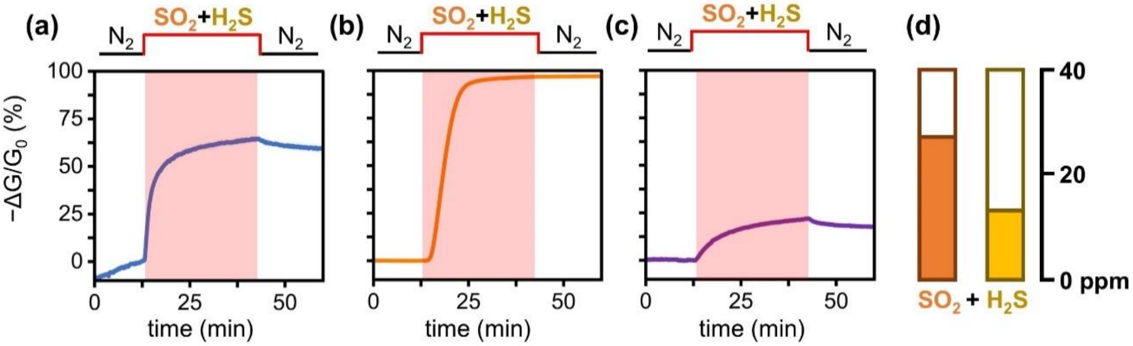

Figure S39. Sensing traces of (**a**) $Ni_3(HHTP)_2$, (**b**) $Cu_3(HHTP)_2$, and (**c**) $Zn_3(HHTP)_2$ devices exposed to a mixture of 27 ppm SO_2 and 13 ppm H_2S. (**d**) Plot showing the composition and concentration of the mixture.

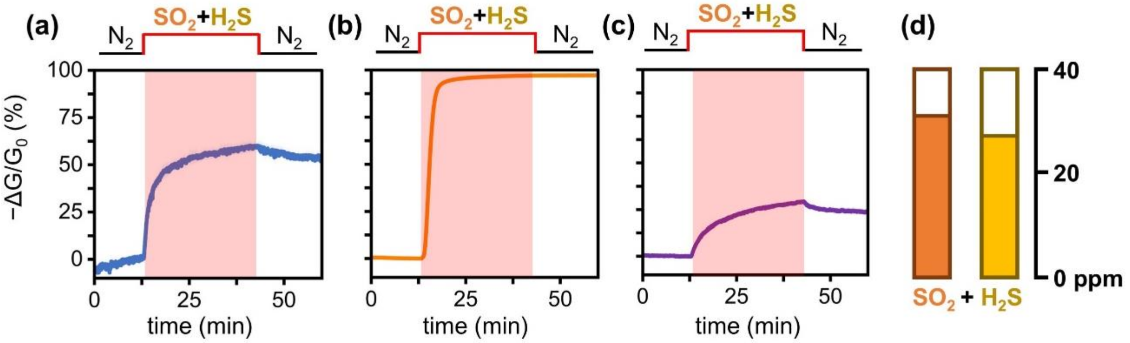

Figure S40. Sensing traces of (**a**) $Ni_3(HHTP)_2$, (**b**) $Cu_3(HHTP)_2$, and (**c**) $Zn_3(HHTP)_2$ devices exposed to a mixture of 31 ppm SO_2 and 27 ppm H_2S. (**d**) Plot showing the composition and concentration of the mixture.

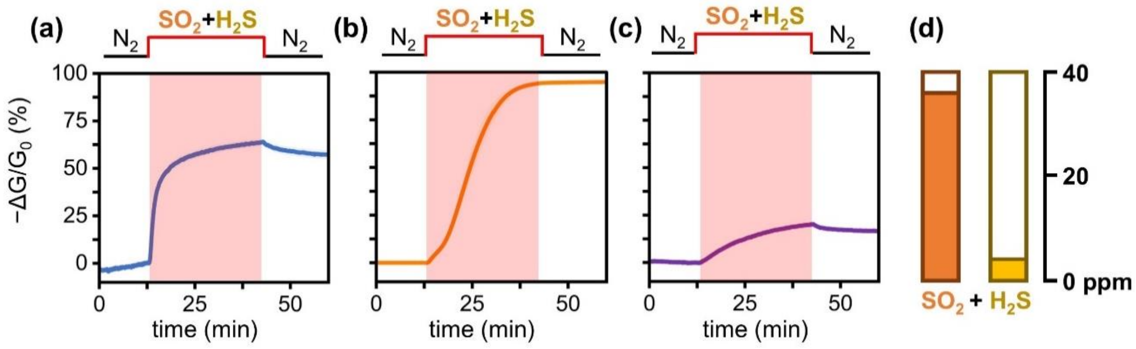

Figure S41. Sensing traces of (**a**) $Ni_3(HHTP)_2$, (**b**) $Cu_3(HHTP)_2$, and (**c**) $Zn_3(HHTP)_2$ devices exposed to a mixture of 36 ppm SO_2 and 4 ppm H_2S. (**d**) Plot showing the composition and concentration of the mixture.

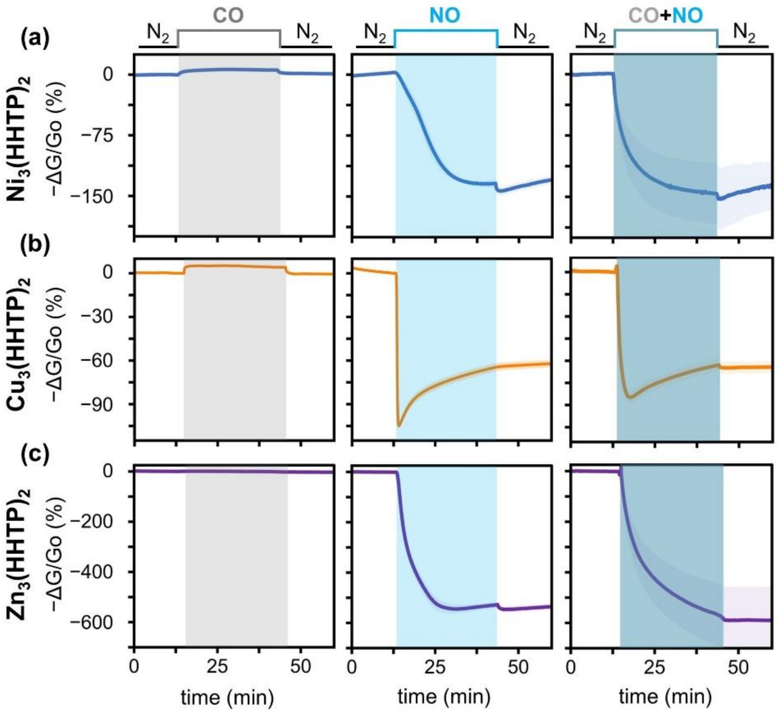

Figure S42. Chemiresistive sensing responses of (**a**) $Ni_3(HHTP)_2$, (**b**) $Cu_3(HHTP)_2$, (**c**) $Zn_3(HHTP)_2$ towards individual exposures of 80 ppm CO and 80 ppm NO alongside simultaneous dual exposure of 80 ppm CO and 80 ppm NO.

Table S12. Experimental limit of detection in ppm for each MOF-analyte pairing.

	**CO**	**NH_3**	**SO_2**	**H_2S**	**NO**
$Ni_3(HHTP)_2$	5	5	5	5	5
$Cu_3(HHTP)_2$	5	5	5	0.5	0.5
$Zn_3(HHTP)_2$	20	5	5	5	5

XII. Metrics for SO_2/H_2S Mixture Classification

To evaluate the performance of our sensor array paired with the random forest for classifying the four SO_2/H_2S mixture classes, we use three metrics: the true positive rate (TPR), false positive rate (FPR), and precision. The value of all these metrics ranges from 0 to 1. For example, let us explain these metrics assuming we want to evaluate how well our model performed on the $SO_2 \uparrow$ & $H_2S \uparrow$ mixture class. First, let us define some basic terminology. In this context, a *positive* is an $SO_2 \uparrow$ & $H_2S \uparrow$ mixture, and a *negative* is not a $SO_2 \uparrow$ & $H_2S \uparrow$ mixture. Then:

- A **true positive (TP)** outcome is when the gas is a $SO_2 \uparrow$ & $H_2S \uparrow$ mixture, and we correctly predict it to be a $SO_2 \uparrow$ & $H_2S \uparrow$ mixture. ☺
- A **false negative (FN)** outcome is when the gas is a $SO_2 \uparrow$ & $H_2S \uparrow$ mixture, but we incorrectly predict it to not be a $SO_2 \uparrow$ & $H_2S \uparrow$ mixture (i.e., to be one of the three other possible mixtures). ☹
- A **false positive (FP)** outcome is when the gas is not a $SO_2 \uparrow$ & $H_2S \uparrow$ mixture, but we incorrectly predict it to be a $SO_2 \uparrow$ & $H_2S \uparrow$ mixture. ☹
- A **true negative (TN)** outcome is when the gas is not a $SO_2 \uparrow$ & $H_2S \uparrow$ mixture, and we correctly predict it to not be a $SO_2 \uparrow$ & $H_2S \uparrow$ mixture. ☺ (Note, a TN includes the case when non- $SO_2 \uparrow$ & $H_2S \uparrow$ mixture is incorrectly predicted as a different non- $SO_2 \uparrow$ & $H_2S \uparrow$ mixture.)

In the confusion matrix below, we circle the outcomes that correspond to TP, FN, FP, and TN---indicated by color. Next, we define the TPR, FPR, and precision in terms of these TP, FN, FP, and TN outcomes.

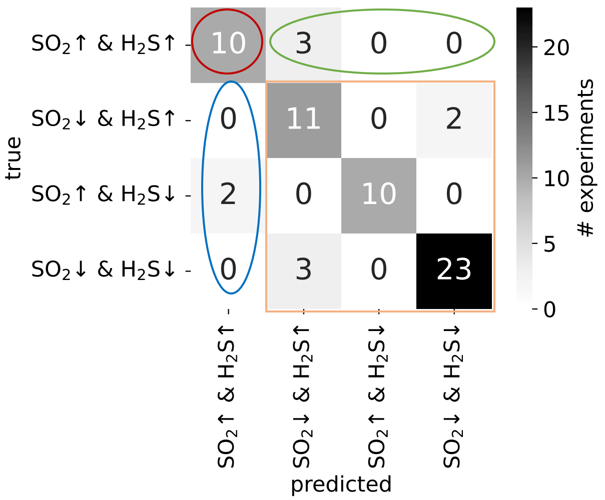

The **True Positive Rate (Recall)** is the fraction of true $SO_2\uparrow$ & $H_2S\uparrow$ mixtures in our test data that we accurately detected. A TPR of 1.0 represents a perfect score. Here, there are 13 true $SO_2\uparrow$ & $H_2S\uparrow$ mixtures, and we correctly identify 10 of them. Hence:

$$TPR = \frac{TP}{TP + FN} = \frac{10}{10 + 3} = \frac{10}{13}$$

1. The **False Positive Rate** is the fraction of non- $SO_2\uparrow$ & $H_2S\uparrow$ mixtures that are incorrectly predicted as $SO_2\uparrow$ & $H_2S\uparrow$ mixtures. A FPR of 0.0 represents a perfect score. Here, there are 51 non- $SO_2\uparrow$ & $H_2S\uparrow$ mixtures, and we incorrectly predicted two of them to be $SO_2\uparrow$ & $H_2S\uparrow$ mixtures. Hence:

$$FPR = \frac{FP}{FP + TN} = \frac{2}{2 + (11 + 2 + 10 + 3 + 23)} = \frac{2}{51}$$

2. The **Precision** is the fraction of the $SO_2\uparrow$ & $H_2S\uparrow$ mixtures that are correctly predicted to be $SO_2\uparrow$ & $H_2S\uparrow$ mixtures. A precision of 1.0 represents a perfect score. Here, there are 12 mixtures we predict to be $SO_2\uparrow$ & $H_2S\uparrow$ mixtures, and ten of these truly are $SO_2\uparrow$ & $H_2S\uparrow$ mixtures. Hence:

$$Precision = \frac{TP}{TP + FP} = \frac{10}{10 + 2} = \frac{10}{12}$$

XIII. Machine Learning Regression & Feature Analysis

Instead of treating the gas prediction task as a classification, we also treat the regression task of predicting the quantitative concentration [ppm] of both H_2S and SO_2 from the response of the sensor array. We evaluated a multi-output random forest regressor with the same leave-one-out cross-validation procedure. The mean absolute error was 6.8 ppm for SO_2 and 6.7 ppm for H_2S (compared to a range of 0-35 ppm exposures). **Figure S43** shows a parity plot. While the quantitative accuracy is not highly impressive, **Figure S43** still shows the utility of the sensor array for distinguishing between the four categories of SO_2/H_2S concentrations at the 20 ppm cutoff that we presented, which represents a marked improvement in sensor array technology.

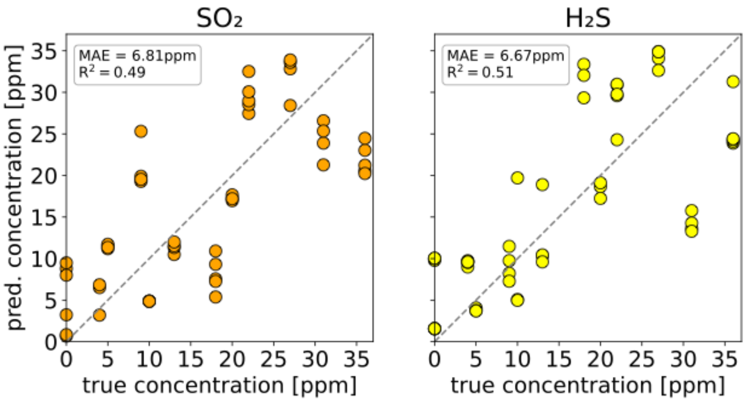

Figure S43. Treating the gas prediction task as a regression. Within a leave-one-concentration-out cross validation loop, we train a two-output random forest *regressor* to predict the quantitative concentration vector ([SO_2] ppm, [H_2S] ppm) from the response vector of the sensor array. This parity plot compares the true and predicted concentrations in the gas phase and displays performance metrics (mean absolute error and coefficient of determination) in the legend.

Figure S44 shows average (over the leave-one-out cross validation procedure) impurity-based importance scores for the three features of the sensing trace used as input for the random forest classifier. We find all three features were roughly equally important. However, this should be interpreted with caution, because the features are highly correlated (See **Figure S45**.).

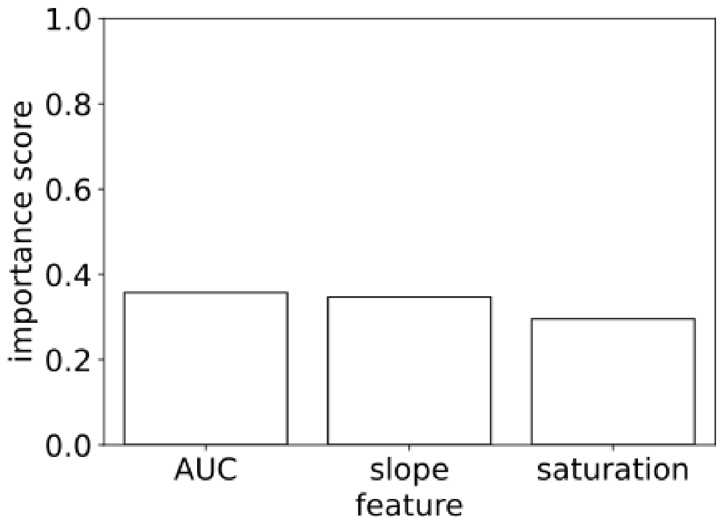

Figure S44. Average (over cross-validation) impurity-based importance scores for the features of the sensing trace for the random forest classifier.

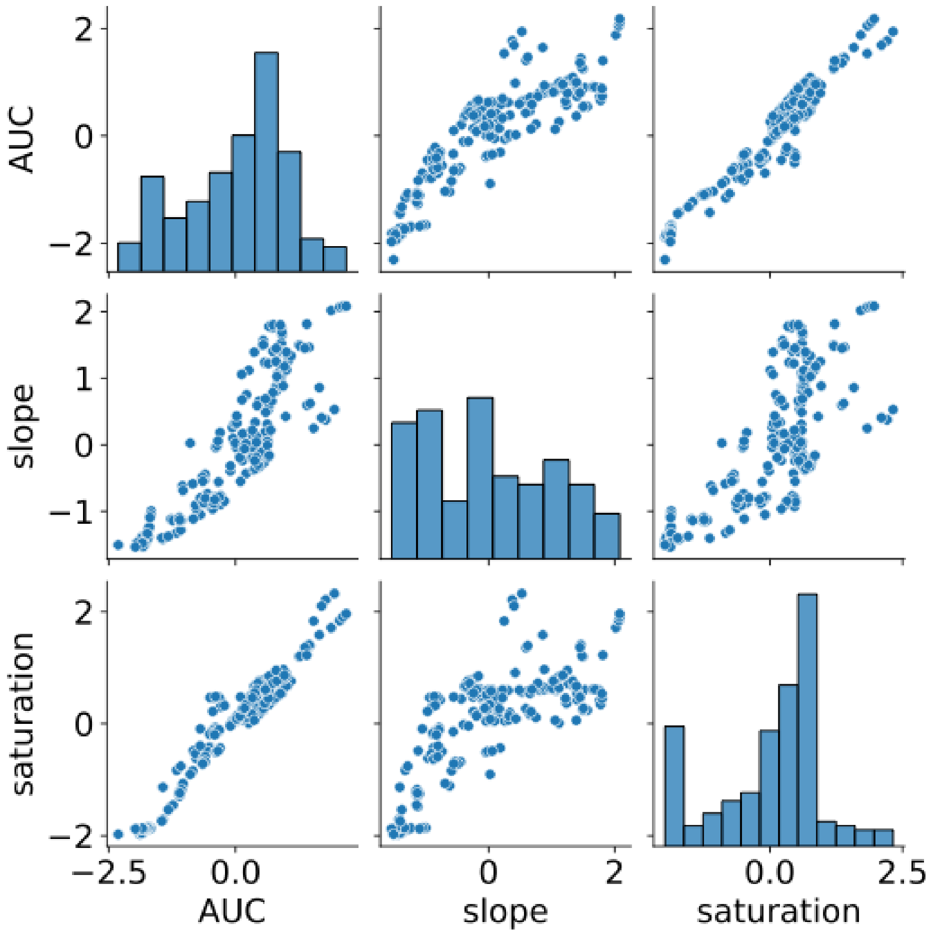

Figure S45. The correlations between and distributions of the three [transformed] features of the sensing trace we use as input to the random forest classifier.

XIV. Spectroscopic Assessment of Material−Analyte Interactions

To assess the MOF materials using diffuse reflectance infrared Fourier transform spectroscopy (DRIFTS), a double beam difference spectra approach was used to identify key material changes upon analyte exposure. For each DRIFTS experiment regardless of analyte identity, the sample cup was loaded with ground, oven dried KBr and heated to 80°C in N_2 flow (2 bubbles/second) for 20 minutes. A single-beam measurement of the KBr was recorded at room temperature to use as background for subsequent experiments. All MOF:KBr samples were prepared by gentle grinding 1 mg of MOF with 18 mg of KBr using a small mortar and pestle until well combined with a light grey color throughout. The MOF:KBr samples were dried in a vacuum oven overnight. MOF:KBr samples were then loaded into the sample cup and further dried by heating to 80°C in N_2 flow (2 bubbles/second) for 20 minutes. Pristine MOF spectra were collected at room temperature as an absorbance spectrum with KBr as a background. A single-beam measurement of the MOF:KBr material was recorded to serve as the background for subsequent experiments. During each experiment, the MOF:KBr material was exposed to 1% analyte in dry N_2 at a flow rate of 2 bubbles/second, followed by purging with dry N_2. Difference spectra were recorded at 0, 2, 4, 6, 8, 10, 15, and 20 minutes of analyte exposure, followed by 30−60 minutes of purging with N_2. The initial MOF:KBr single-beam spectrum was subtracted from each measurement and processed using the Kubelka-Munk transformation.

XPS experiments were performed to probe the interactions between the MOF and binary mixtures of SO_2 and H_2S during simultaneous exposure. Three types of XPS experiments were performed: exposure of MOF powder to i) 40 ppm SO_2, ii) 40 ppm H_2S, and iii) a mixture of 40 ppm SO_2 and 40 ppm H_2S. MOF powders were exposed to the gas or gases in a carrier gas of N_2 over the course of 1 hour, then capped and analyzed via XPS. As a point of comparison, pristine MOF exposed to air was also analyzed by XPS. Peaks present in variable ratios in S the 2p regions of 168.13−169.45 eV, 166.04 eV, and 163.36−164.80 eV correspond to variable concentrations of sulfate (SO_4^{2-}),[7] sulfite (SO_3^{2-}),[8] and polysulfides (S_x) and sulfides (S^{2-})[6,9,10] left on the surface of the material.

XIV.a. DRIFTS of $Ni_3(HHTP)_2$ exposed to SO_2

Upon exposure to 1% SO_2 in dry N_2 followed by N_2 purging, $Ni_3(HHTP)_2$ exhibited seven notable spectral features (**Figure S46**). *First*, during SO_2 exposure, the increasing intensity of select bands indicates the presence of free SO_2. For instance, bands at 1373, 1360, and 1348 cm^{-1} were assigned to the ν_3 asymmetric stretch of SO_2, bands at 2512 and 2498 cm^{-1} were assigned to the ν_1 symmetric stretch of SO_2, and bands at 1164 and 1140 cm^{-1} were assigned to the δ mode of SO_2.[11] *Second*, upon purging with N_2, the spectral features attributed to free SO_2 diminished significantly but not completely. Despite 60 minutes of purging with dry N_2 there remained noticeable bands at 1366 and 1154 cm^{-1}, which we attribute to ν_3 and δ mode of adsorbed SO_2. The retention of a peak despite prolonged purging indicates retention of chemisorbed SO_2 on the framework.[12] *Third*, positive-going bands around 1257 and 1221 cm^{-1} were assigned to adsorbed SO_2.[12] It is likely that this adsorbed SO_2 is in a different chemical environment compared to the SO_2 resulting in the peaks at 1366 and 1154 cm^{-1}. *Fourth*, the positive-going peaks at 1033 and 1000 cm^{-1} were assigned to the first asymmetric stretch (ν_3) and first symmetric stretch (ν_1) of sulfate (SO_4^{2-}) in with C_{3v} symmetry (**Figure S47**).[13, 14] Based on this DRIFTS peak in conjunction with XPS data (see **Section XIV.g.**), it is likely that SO_2 oxidizes to SO_4^{2-} upon interaction with $Ni_3(HHTP)_2$. *Fifth*, positive-going peaks at 1667−1366 cm^{-1} were attributed to the strengthening of certain modes (vibrations of C=O and C=C) in the HHTP ligand upon SO_2 exposure (**Figure S47**). *Sixth*, the negative-going peak at 3630 cm^{-1} was attributed to −OH indicating a removal of hydroxyl groups from bound aqua ligands as the possible source for the oxidation of SO_2 to SO_4^{2-}.[15, 16] *Seventh*, following 60 minutes of purging with N_2 the spectral changes did not return to baseline indicating irreversibility of material changes post exposure.

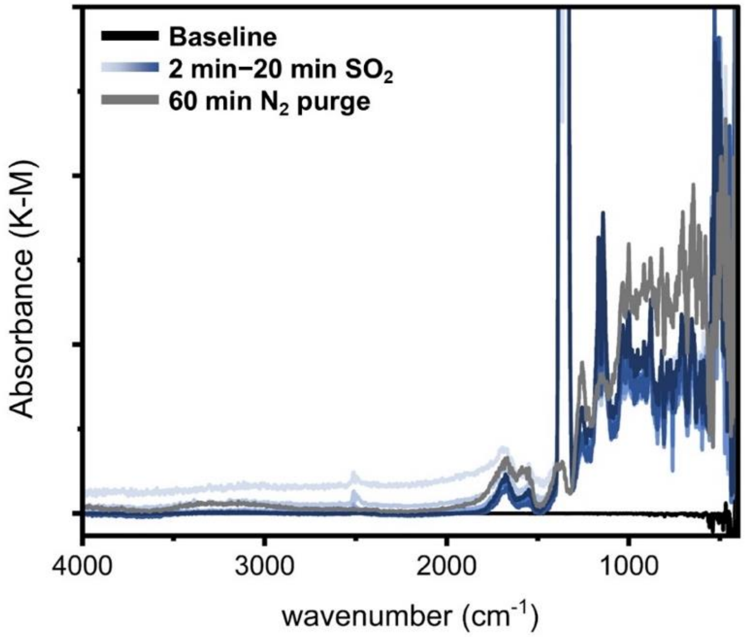

Figure S46. Time resolved DRIFTS difference spectra of $Ni_3(HHTP)_2$ exposed to 1% SO_2 in dry N_2 for 20 minutes followed by purging in dry N_2 for 60 minutes.

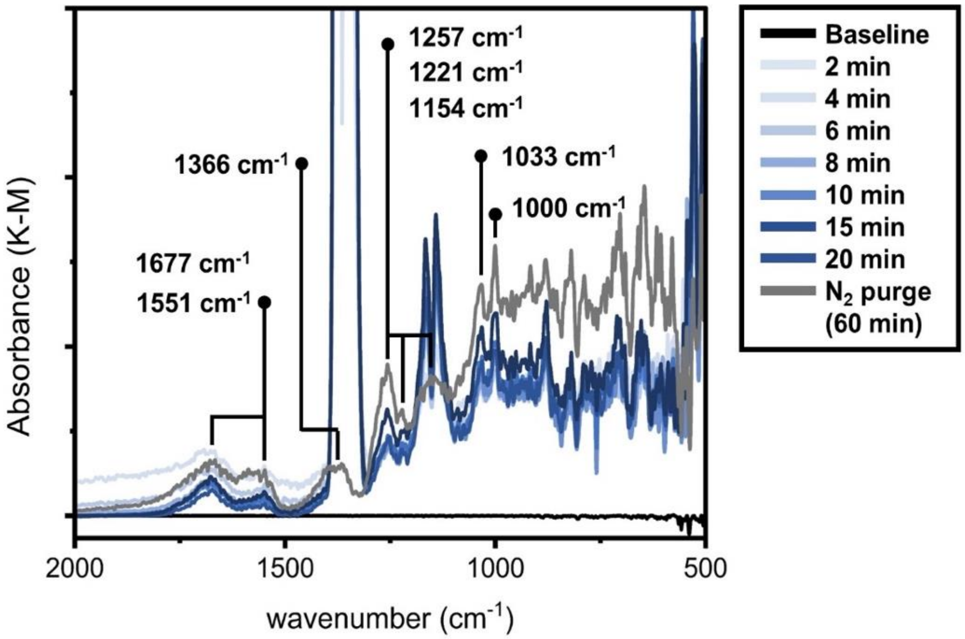

Figure S47. Zoomed-in time resolved DRIFTS difference spectra of $Ni_3(HHTP)_2$ exposed to 1% SO_2 in dry N_2 for 20 minutes followed by purging in dry N_2 for 60 minutes with key peaks labeled.

XIV.b. DRIFTS of $Cu_3(HHTP)_2$ exposed to SO_2

When exposed to 1% SO_2 in dry N_2 followed by N_2 purging, $Cu_3(HHTP)_2$ exhibited six notable spectral features (**Figure S48**). Some observations for $Cu_3(HHTP)_2$ were similar to those observed in the case of $Ni_3(HHTP)_2$. For instance, *first*, the same bands for the v_3, v_1, and δ mode of free SO_2 were observed at the following respective peak positions: 1) 1373, 1360, and 1348 cm^{-1}, 2) 2512 and 2498 cm^{-1}, and 3) 1164 and 1140 cm^{-1}. Additionally, positive going bands at 1708 and 1412 cm^{-1} were observed and attributed to the HHTP ligand indicating strengthening of the C=O and C=C vibrational modes **(Figure S49**). Similarly with SO_2 exposure to $Ni_3(HHTP)_2$, the $Cu_3(HHTP)_2$ spectra also exhibited persistent peaks at 1260 and 1037 cm^{-1} despite purging for 60 minutes, indicative of chemisorbed SO_2 and bound SO_4^{2-}, respectively.[12, 13] (**Figure S49**). *Fourth*, a negative-going band attributed to −OH red-shifted from 3644 cm^{-1} to 3585 cm^{-1} from 2 to 20 minutes of SO_2 exposure. Following purging with N_2, the band blue-shifted back to 3671 cm^{-1}. Based on these shifts, it is likely that SO_2 interacted with bound hydroxyls on the framework as a site of adsorption.[15, 16] *Fifth*, there was a decrease in background absorbance across the length of the experiment, indicating electronic changes in the material. *Sixth*, upon purging with N_2, the spectra started to return to baseline as opposed to the $Ni_3(HHTP)_2$. This observation indicates that the SO_2 interaction is more reversible with $Cu_3(HHTP)_2$ than $Ni_3(HHTP)_2$.

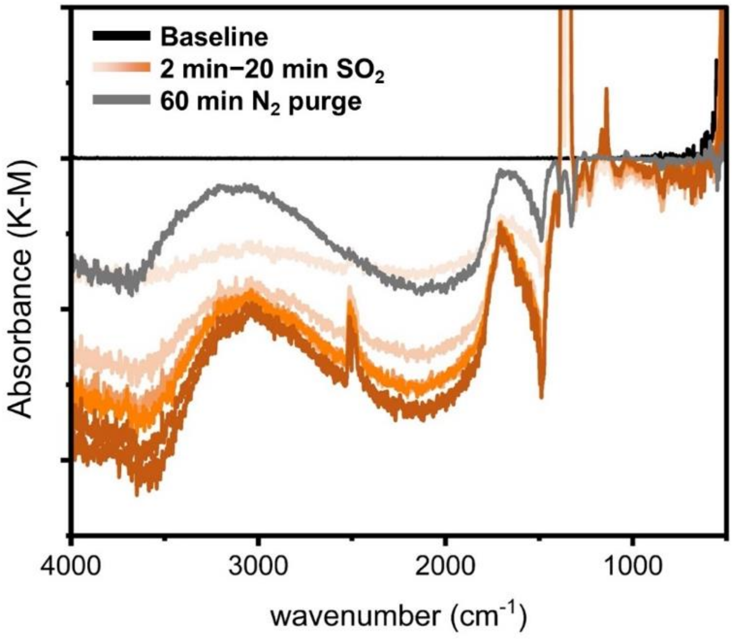

Figure S48. Time resolved DRIFTS difference spectra of $Cu_3(HHTP)_2$ exposed to 1% SO_2 in dry N_2 for 20 minutes followed by purging in dry N_2 for 60 minutes.

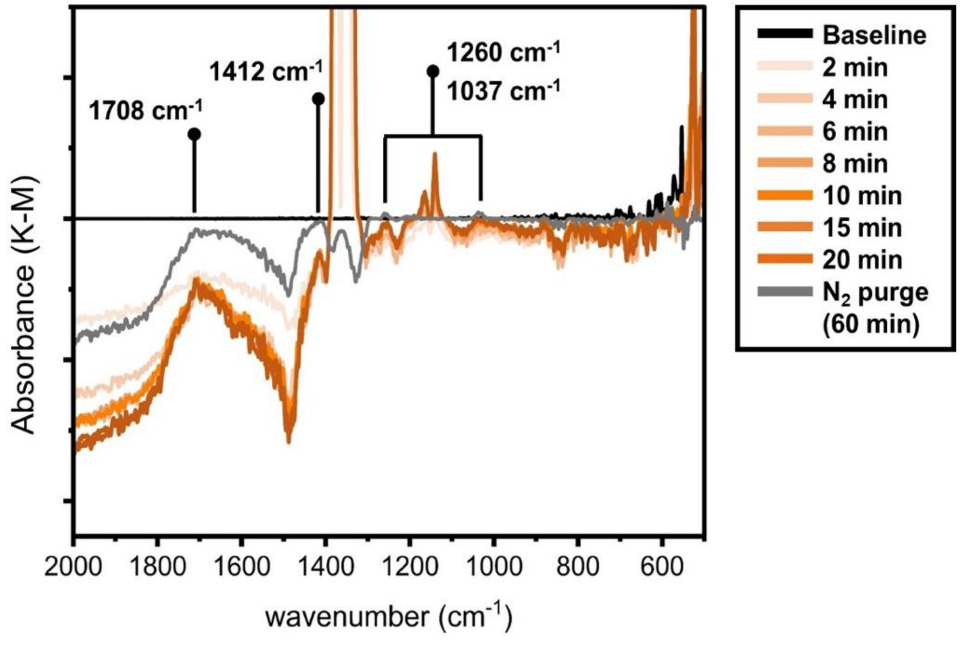

Figure S49. Zoomed-in time resolved DRIFTS difference spectra of $Cu_3(HHTP)_2$ exposed to 1% SO_2 in dry N_2 for 20 minutes followed by purging in dry N_2 for 30 minutes with key peaks labeled.

XIV.c. DRIFTS of $Zn_3(HHTP)_2$ exposed to SO_2

Upon exposure to 1% SO_2 in dry N_2 followed by N_2 purging, $Zn_3(HHTP)_2$ exhibited four notable spectral features (**Figure S50**). First, free SO_2 peaks were observed as described in the Ni-, and $Cu_3(HHTP)_2$ experiments at the following positions: 1) 1373, 1360, and 1348 cm^{-1}, 2) 2514 and 2497 cm^{-1}, and 3) 1165 and 1141 cm^{-1} corresponding to the v_3, v_1, and δ mode of free SO_2, respectively. Second, a positive-going peak at 1671 cm^{-1} was attributed to HHTP ligand stretching modes (**Figure S51**). Lastly, as was the case with $Cu_3(HHTP)_2$, there was an observed decrease in background absorbance during SO_2 exposure and semi-reversibility in spectral features upon N_2 purging. No peaks attributed to S-containing species were observed. *Fourth*, a negative-going broad peak at 3608 cm^{-1} indicates a decrease in hydroxyl content as observed with the Ni- and $Cu_3(HHTP)_2$ materials.

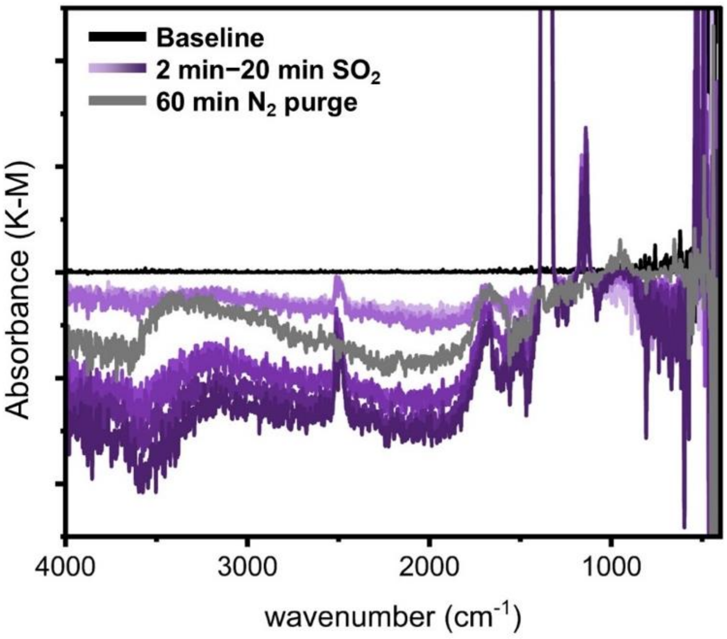

Figure S50. Time resolved DRIFTS difference spectra of $Zn_3(HHTP)_2$ exposed to 1% SO_2 in dry N_2 for 20 minutes followed by purging in dry N_2 for 60 minutes.

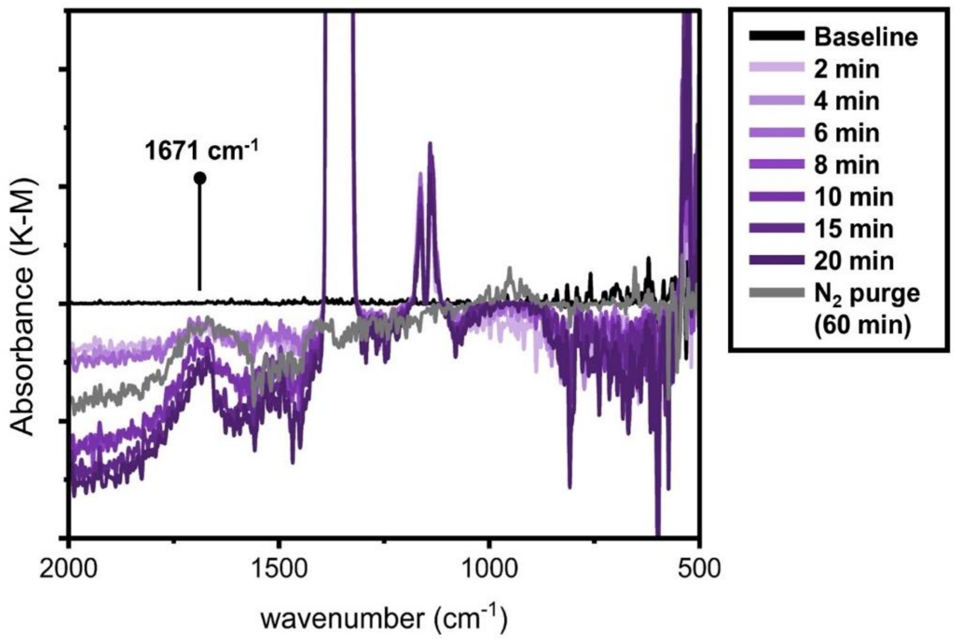

Figure S51. Zoomed-in time resolved DRIFTS difference spectra of $Zn_3(HHTP)_2$ exposed to 1% SO_2 in dry N_2 for 20 minutes followed by purging in dry N_2 for 60 minutes with key peaks labeled.

XIV.d. DRIFTS of $Ni_3(HHTP)_2$ exposed to H_2S

Upon exposure to 1% H_2S in dry N_2 followed by N_2 purging, $Ni_3(HHTP)_2$ exhibited six notable spectral features (**Figure S52**). *First,* throughout the course of the experiment, the material exhibited a decrease in background absorbance across the range of ~4000−1200 cm^{-1}, which was previously observed with H_2S exposure to the same material.[6] *Second,* a broad peak at 3409 cm^{-1} and small peak at 1699 cm^{-1} , which correspond to stretching and bending modes of H_2O, respectively, indicate that surface bound H_2O likely changes due to H_2S exposure. *Third,* negative-going bands at 1513, 1383, 1320, and 545 cm^{-1} correspond in both position and shape to the characteristic peaks observed in the pristine $Ni_3(HHTP)_2$ spectra (1482, 1367, 1303, and 547 cm^{-1}, respectively) (**Figure S53**). These peaks are assigned to HHTP ligand characteristic bands such as ν(C=O) and ν(C=C) and Ni–O modes. We attribute these peak shifts and decreases in peak intensity to an overall degradation of the MOF material, suggesting that H_2S exposure at high concentration compromises the material's crystallinity. It has been previously reported that the crystallinity of another HHTP-based MOF, $Cu_3(HHTP)_2$, decreases following exposure to 1% H_2S.[6] Couple with literature precedent for absorbance shifts and decreases because of the reduction of material crystallinity,[17] we attribute these negative-going bands to the disruption of long-range order in the material on the HHTP-ligand environment. The reduction of crystallinity and subsequent change in secondary interactions in turn affects the vibrational modes of the material. *Fourth,* the positive-going bands at 1257, and 1145 cm^{-1} were attributed to SO_2 adsorbed on the framework.[12] *Fifth,* the positive-going band at 999 cm^{-1} was attributed to the presence of SO_4^{2-}, which was confirmed by XPS data (see **Section XIV.g.**) (**Figure S53**).[13] With both S-containing analytes, the $Ni_3(HHTP)_2$ directs analyte oxidation to sulfate. *Sixth,* the spectral features following 20 minutes of 1% H_2S exposure were retained even after 60 minutes of purging in dry N_2, indicating that H_2S exposure to $Ni_3(HHTP)_2$ at this concentration causes irreversible material changes. This finding is consistent with previous studies of the same analyte and material.[6]

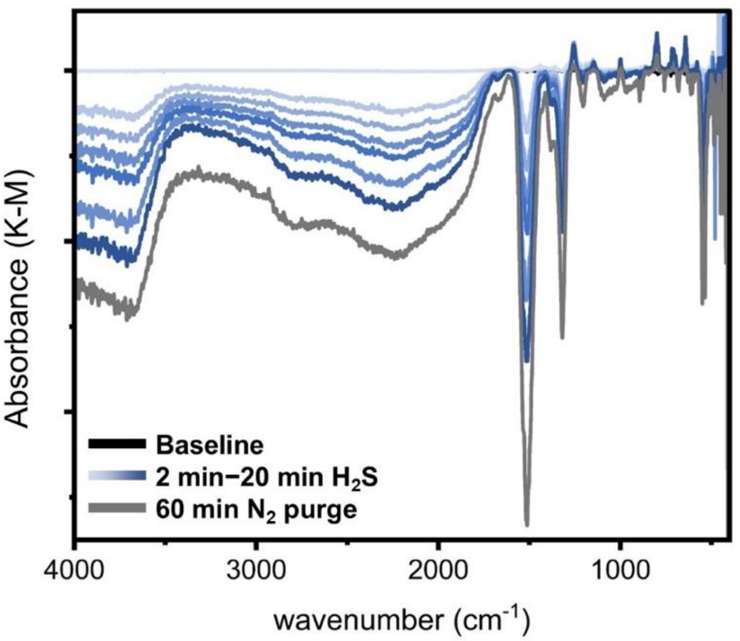

Figure S52. Time resolved DRIFTS difference spectra of $Ni_3(HHTP)_2$ exposed to 1% H_2S in dry N_2 for 20 minutes followed by purging in dry N_2 for 60 minutes.

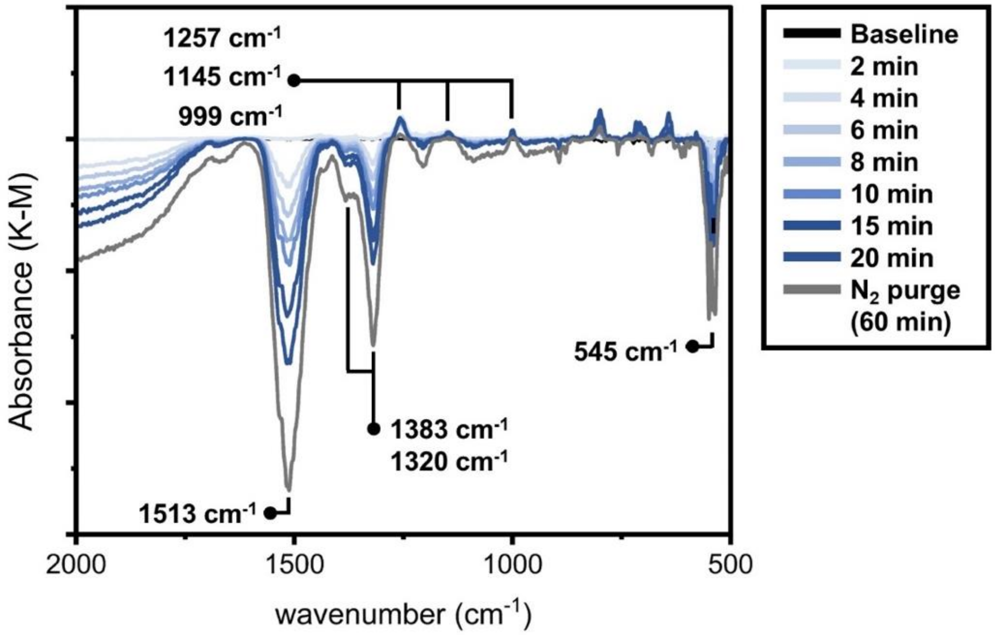

Figure S53. Zoomed-in time resolved DRIFTS difference spectra of $Ni_3(HHTP)_2$ exposed to 1% H_2S in dry N_2 for 20 minutes followed by purging in dry N_2 for 60 minutes with key peaks labeled.

XIV.e. DRIFTS of $Cu_3(HHTP)_2$ exposed to H_2S

Upon exposure to 1% H_2S in dry N_2 followed by N_2 purging, $Cu_3(HHTP)_2$ exhibited four notable spectral features similar to that of the Ni-based material (**Figure S54**). *First*, throughout the course of the experiment, the material exhibited a decrease in background absorbance across the range of ~4000−500 cm^{-1}, which was previously observed with H_2S exposure to the same material.[6] *Second,* a broad peak at ~3400 cm^{-1} and small peak at 1627 cm^{-1} , which correspond to stretching and bending modes of H_2O, respectively, indicate that surface bound H_2O likely changes due to H_2S exposure. *Third,* negative-going bands at 1471, 1375, 1311, and 1222 cm^{-1} correspond in both position and shape to the characteristic peaks observed in the pristine $Cu_3(HHTP)_2$ spectra (1457, 1370, 1302, and 1222, respectively) (**Figure S55**). These peaks are assigned to HHTP ligand characteristic bands such as ν(C=O) and ν(C=C). Additionally, negative-going bands between 850−500 cm^{-1} were also observed, which we assign to characteristic $Cu_3(HHTP)_2$ peaks indicative of C−O and Cu−O modes. We hypothesized that the decrease in characteristic $Cu_3(HHTP)_2$ peaks arises from a decrease in material crystallinity as similarly noted in the case of the Ni-based MOF. *Fourth,* the spectral features following 20 minutes of 1% H_2S exposure were retained after 30 minutes of purging in dry N_2, indicating that H_2S exposure to $Cu_3(HHTP)_2$ at this concentration causes irreversible material changes. This finding is consistent with previous studies of the same analyte and material.[6] No peaks indicative of H_2S or any sulfur-containing species interacting with the MOF were observed, likely due to the low wavelength of Cu−S vibrations. If polysulfides or other sulfur species (besides SO_4^{2-}) were formed, their characteristic modes may be too low in wavelength to distinguish, given the noise at these low wavelengths and the limitations of the experimental setup.

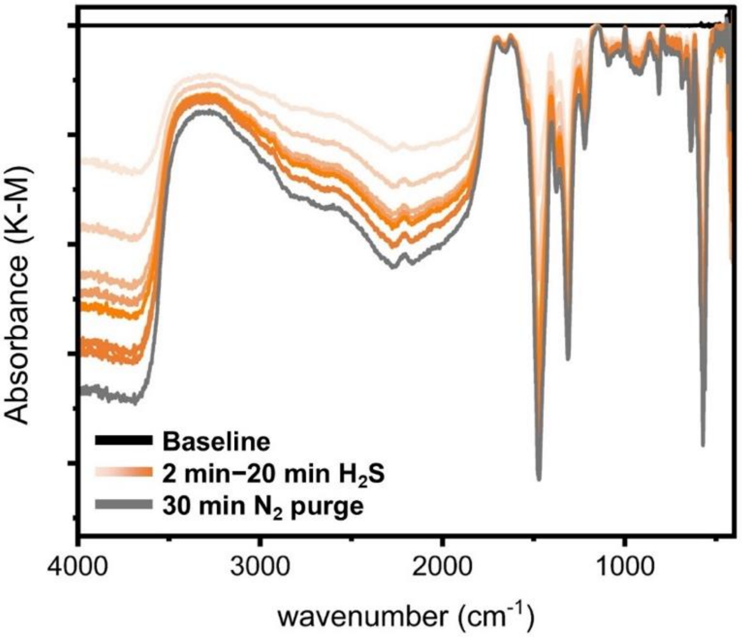

Figure S54. Time resolved DRIFTS difference spectra of $Cu_3(HHTP)_2$ exposed to 1% H_2S in dry N_2 for 20 minutes followed by purging in dry N_2 for 30 minutes.

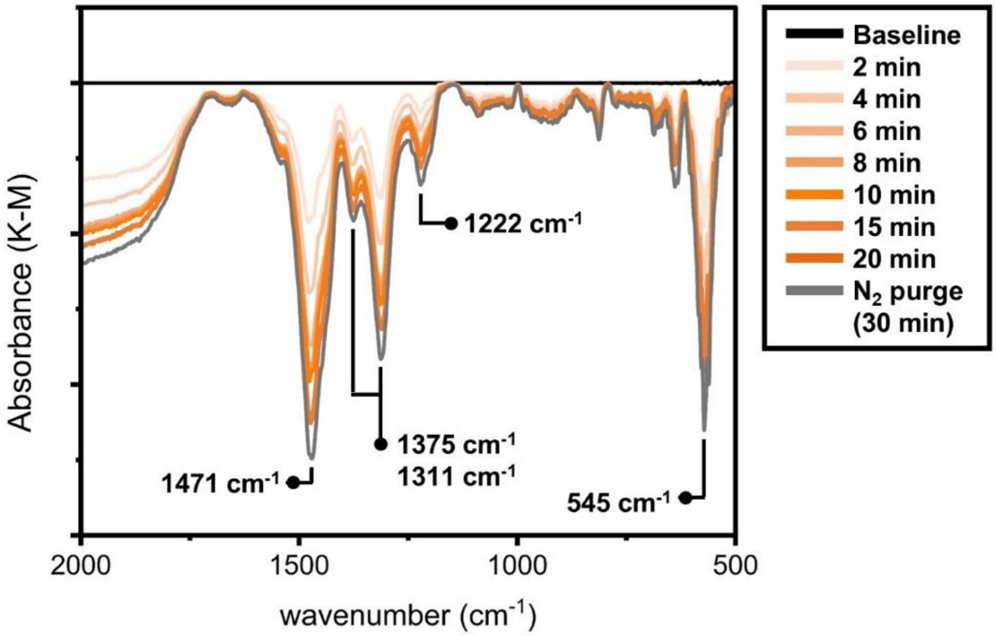

Figure S55. Zoomed-in time resolved DRIFTS difference spectra of $Cu_3(HHTP)_2$ exposed to 1% H_2S in dry N_2 for 20 minutes followed by purging in dry N_2 for 30 minutes with key peaks labeled.

XIV.f. DRIFTS of $Zn_3(HHTP)_2$ exposed to H_2S

Upon exposure to 1% H_2S in dry N_2 followed by N_2 purging, $Zn_3(HHTP)_2$ exhibited four notable spectral features (**Figure S56**). *First*, throughout the course of the experiment, the material exhibited a decrease in background absorbance across the range of ~4000−500 cm^{-1}, similar to the Ni-, and Cu-based MOFs. *Second*, a broad peak at ~3407 cm^{-1} and small peak at 1625 cm^{-1} , which correspond to stretching and bending modes of H_2O, respectively, indicate that surface bound H_2O likely changes due to H_2S exposure. *Third*, negative-going bands at 1559, 1320, and 683 cm^{-1} correspond in both position and shape to the characteristic HHTP ligand peaks observed in the pristine $Zn_3(HHTP)_2$ spectra (1464, 1363, and 547, respectively) (**Figure S57**). Similarly to Ni-, and Cu-based MOFs, we attribute the decrease in these ligand peaks to a decrease in material crystallinity. It is important to note that the low signal-to-noise observed in this experiment compared to the two previous experiments likely arises due to minimal interaction between the analyte and material. The change of absorbance for $Zn_3(HHTP)_2$ material upon H_2S exposure was minimal compared to the change in absorbance for the Ni-, and Cu-containing MOFs despite the same sample preparation and set up. The extent of absorbance change observed through DRIFTS analysis mirrors the magnitude of chemiresistive response upon device exposure (Cu > Ni > Zn in terms of responsivity). *Fourth*, the spectral features following 20 minutes of 1% H_2S exposure were retained even after 60 minutes of purging in dry N_2, indicating that H_2S exposure to $Zn_3(HHTP)_2$ at this concentration causes irreversible material changes as seen with both the Ni-, and Cu-based MOFs.

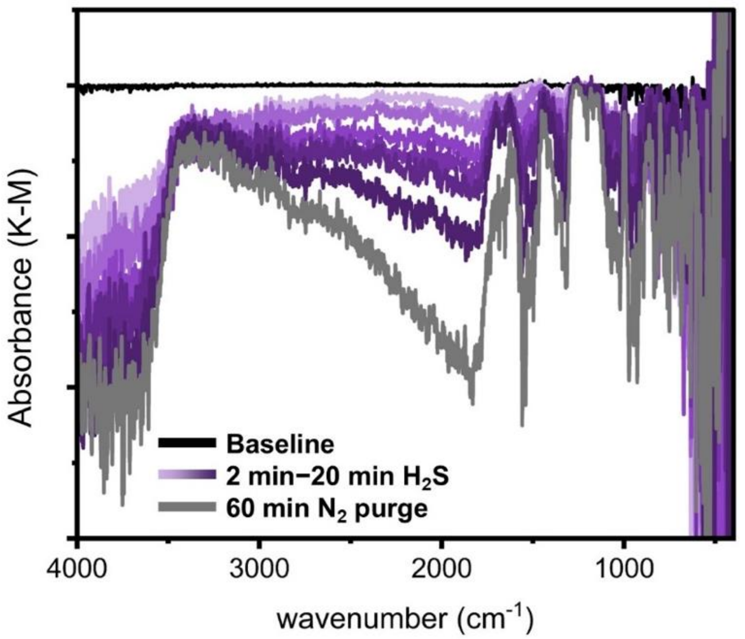

Figure S56. Time resolved DRIFTS difference spectra of $Zn_3(HHTP)_2$ exposed to 1% H_2S in dry N_2 for 20 minutes followed by purging in dry N_2 for 60 minutes.

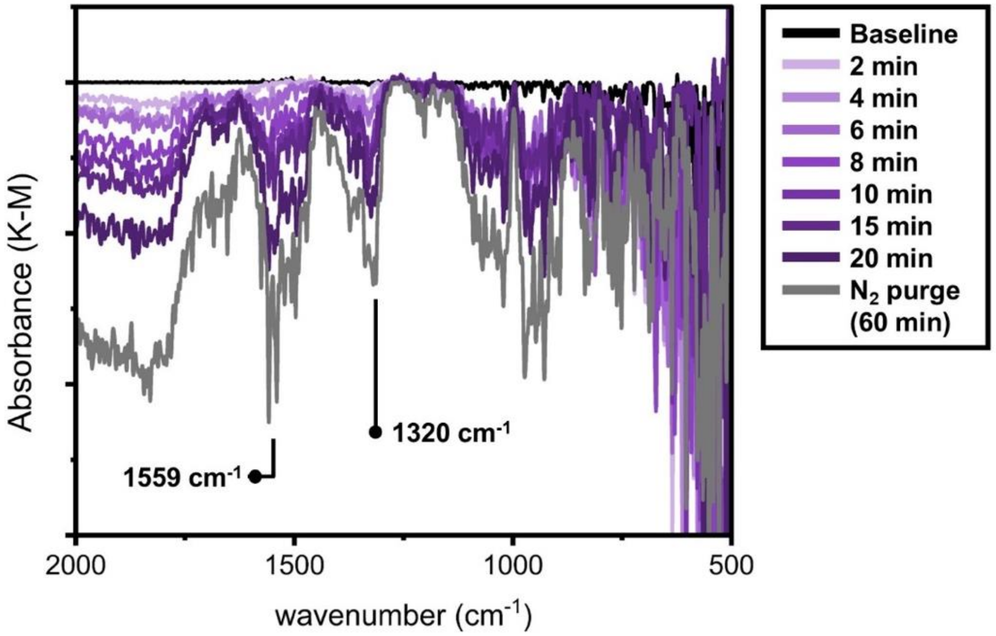

Figure S57. Zoomed-in time resolved DRIFTS difference spectra of $Zn_3(HHTP)_2$ exposed to 1% H_2S in dry N_2 for 20 minutes followed by purging in dry N_2 for 60 minutes with key peaks labeled.

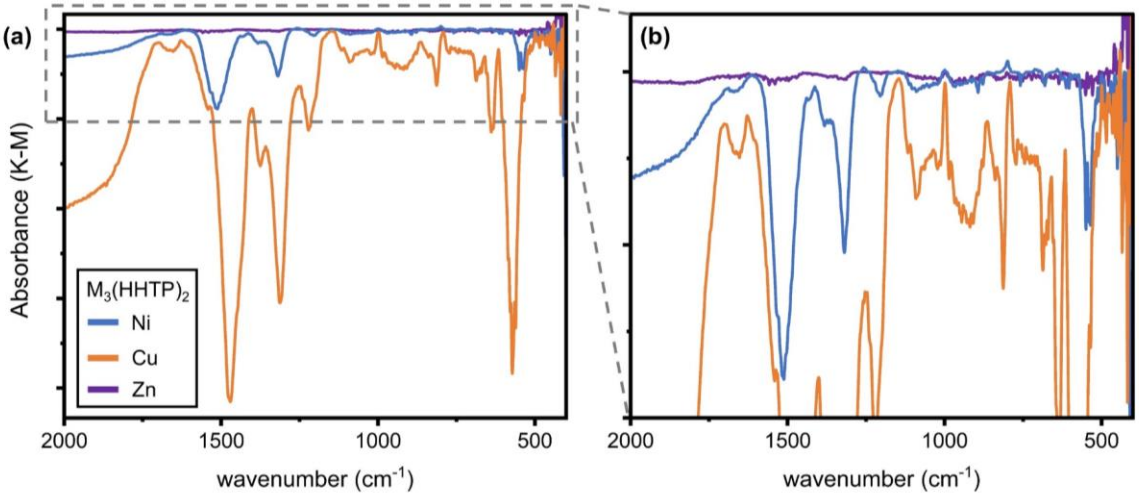

Figure S58. Comparative time resolved DRIFTS difference spectra of $Ni_3(HHTP)_2$ (blue), $Cu_3(HHTP)_2$ (orange), and $Zn_3(HHTP)_2$ (purple) exposed to 1% H_2S in dry N_2 for 20 minutes followed by purging in dry N_2. (**a**) DRIFTS spectra with absorbance intensity set to view all MOF traces. (**b**) DRIFTS spectra with zoomed-in absorbance intensity to discern spectral features of $Zn_3(HHTP)_2$. Note: The y-axis shows absorbance intensity without normalization, allowing for direct comparisons of spectral intensities across the three MOFS.

XIV.g. XPS of $Ni_3(HHTP)_2$ exposed to SO_2, H_2S, and a mixture of SO_2 & H_2S

Following exposure to SO_2 and dual analyte exposure, the Ni-based MOF exhibited peaks in the S 2p region indicative of mixtures of sulfate (SO_4^{2-}), sulfite (SO_3^{2-}), and polysulfides (S_x) (**Figure S59**). However, upon individual exposure to H_2S alone, the only sulfur peak was attributed to SO_4^{2-} and SO_3^{2-}. Following exposure to all analytes, either individual or mixture exposures, there were no changes to the Ni 2p metal center oxidation state and minimal change in peak position. (**Figure S60**. As such, we hypothesize that the reduction to the MOF upon exposure to one or two oxidizing gases (SO_2 and/or H_2S) occurs at the HHTP ligand. This hypothesis is evidenced by an increase in C singly bound to O as compared to C=O content shown in the C 1s region (**Figure S61**).

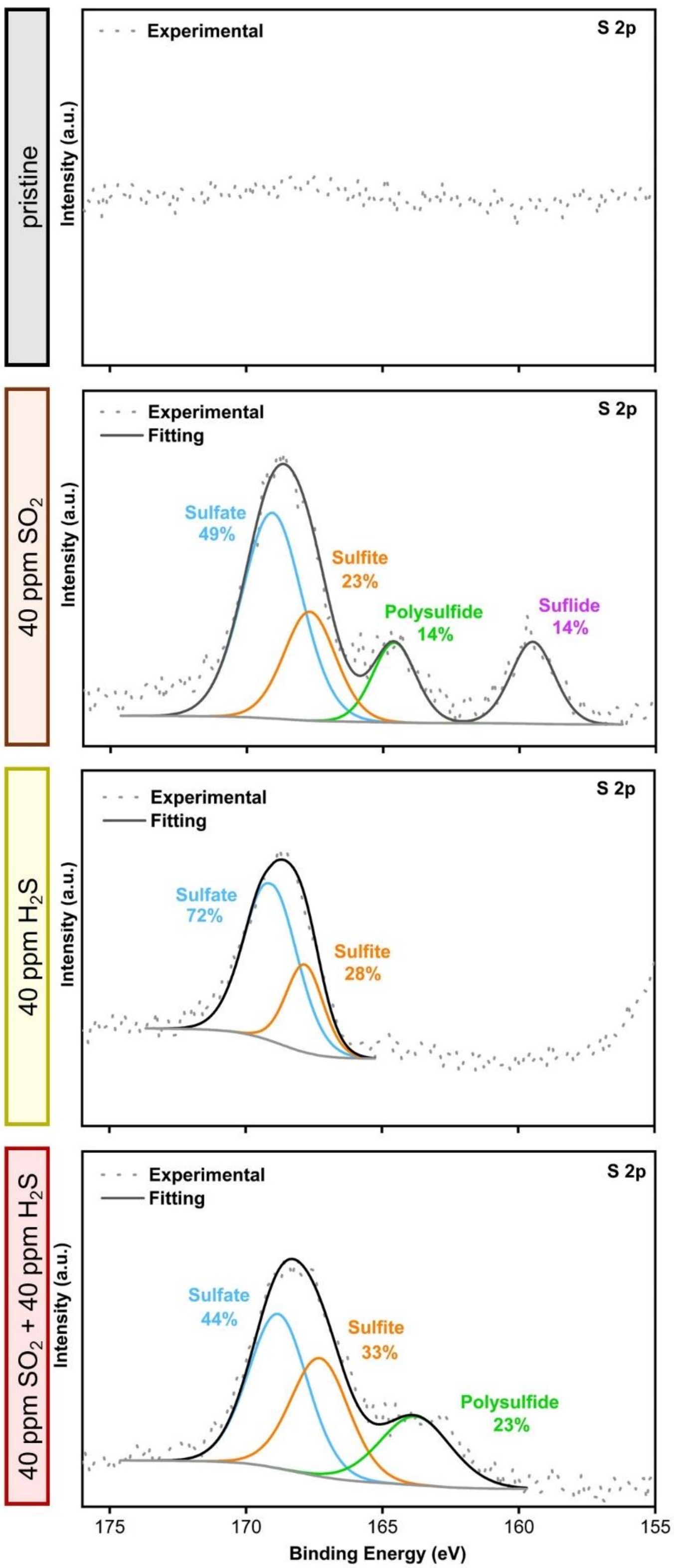

Figure S59. High-resolution ex situ XPS spectra in the S 2p region comparing pristine $Ni_3(HHTP)_2$, and material exposed to 40 ppm SO_2, 40 ppm H_2S, and dual exposure of both 40 ppm SO_2 and 40 ppm H_2S.

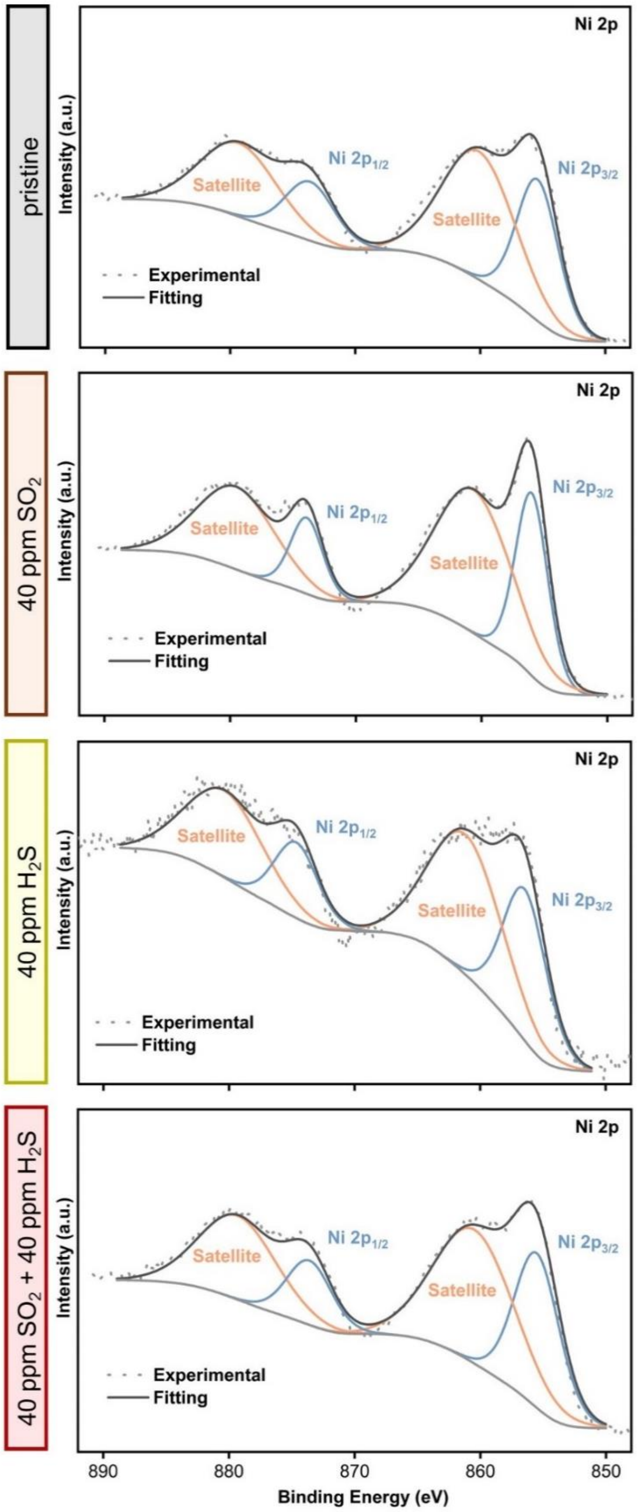

Figure S60. High-resolution ex situ XPS spectra in the Ni 2p region comparing pristine $Ni_3(HHTP)_2$, and material exposed to 40 ppm SO_2, 40 ppm H_2S, and dual exposure of both 40 ppm SO_2 and 40 ppm H_2S.

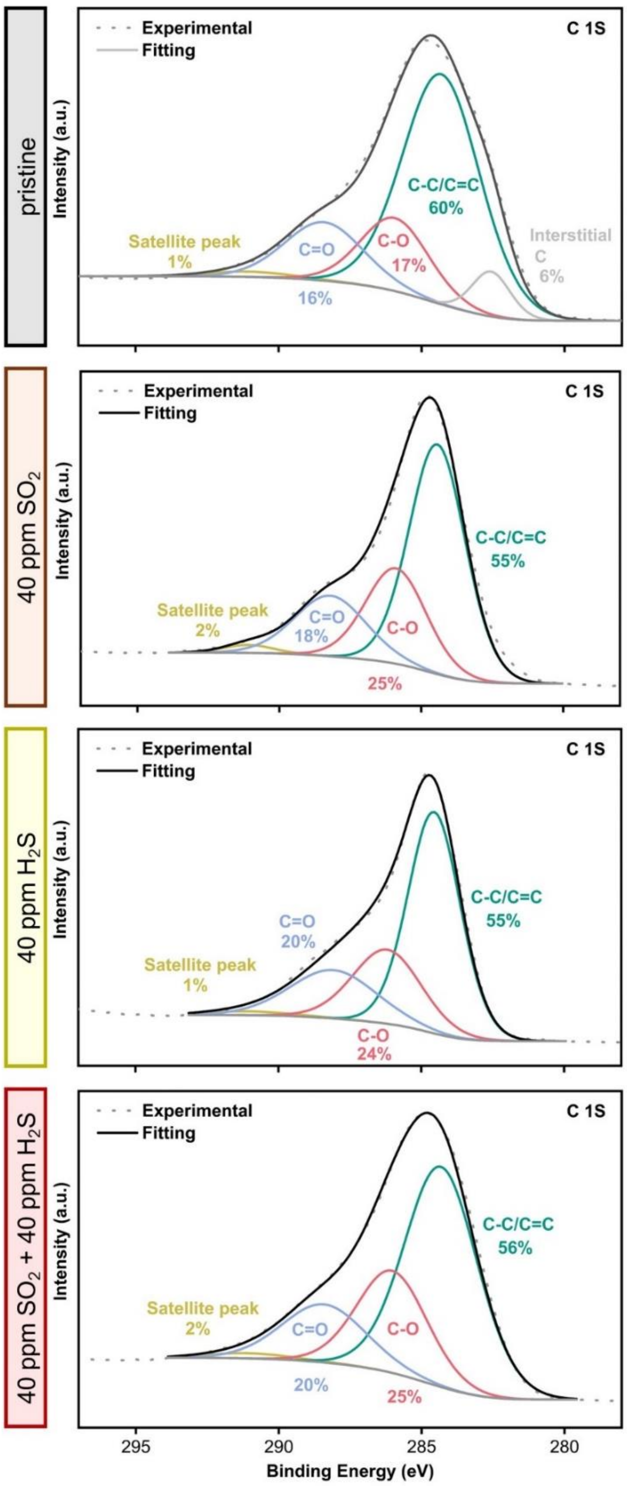

Figure S61. High-resolution ex situ XPS spectra in the C 1s region comparing pristine $Ni_3(HHTP)_2$, and material exposed to 40 ppm SO_2, 40 ppm H_2S, and dual exposure of both 40 ppm SO_2 and 40 ppm H_2S.

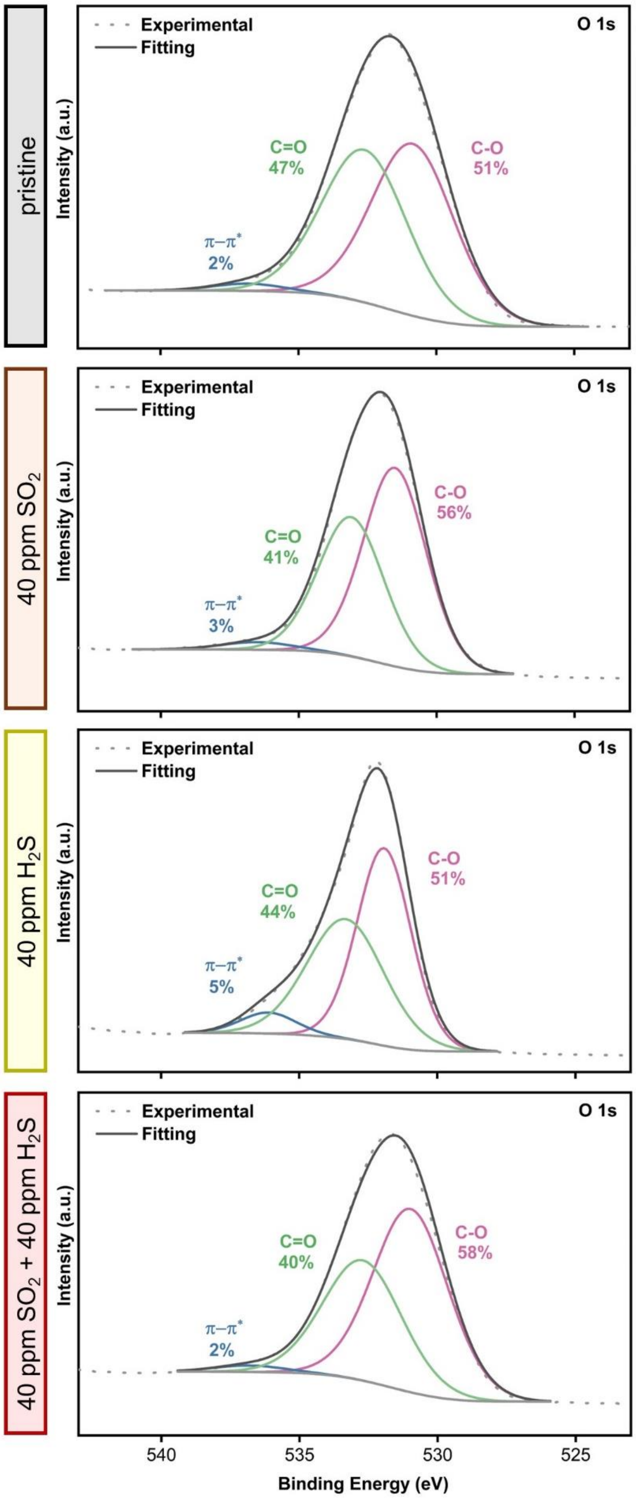

Figure S62. High-resolution ex situ XPS spectra in the O 1s region comparing pristine $Ni_3(HHTP)_2$, and material exposed to 40 ppm SO_2, 40 ppm H_2S, and dual exposure of both 40 ppm SO_2 and 40 ppm H_2S.

XIV.h. XPS of $Cu_3(HHTP)_2$ exposed to SO_2, H_2S, and a mixture of SO_2 & H_2S

Following exposure to SO_2 and H_2S individually, $Cu_3(HHTP)_2$ material contained 100% SO_4^{2-} and a mixture of S-containing products (SO_4^{2-}, SO_3^{2-}, S_x, and S^{2-}), respectively (**Figure S63**). The formation of SO_4^{2-} upon SO_2 exposure to $Cu_3(HHTP)_2$ was corroborated by DRIFTS data. During dual analyte exposure, the mixture of S-containing products (SO_4^{2-}, SO_3^{2-}, S_x, and S^{2-}), was found, but with a higher percent of SO_4^{2-}, and SO_3^{2-} than seen in the single H_2S exposure, due to the presence of SO_2 in the mixture (**Figure S63**). $Cu_3(HHTP)_2$ exhibited major, analyte-directed chemical changes to the metal center. The pristine material exhibited 84% Cu(II) and 16% Cu(I) content, which was reduced to 74% Cu(II) and 26% Cu(I) upon H_2S exposure (**Figure S64**). Upon dual analyte exposure, the copper content was reduced to 77% Cu(II) and 23% Cu(I) from the pristine sample (**Figure S64**). These results align with previous insights in which the sulfur of the extremely reducing H_2S gas attacks the redox-labile Cu center leading to its reduction to Cu (I), resulting in the high selectivity of H_2S over SO_2.[6]

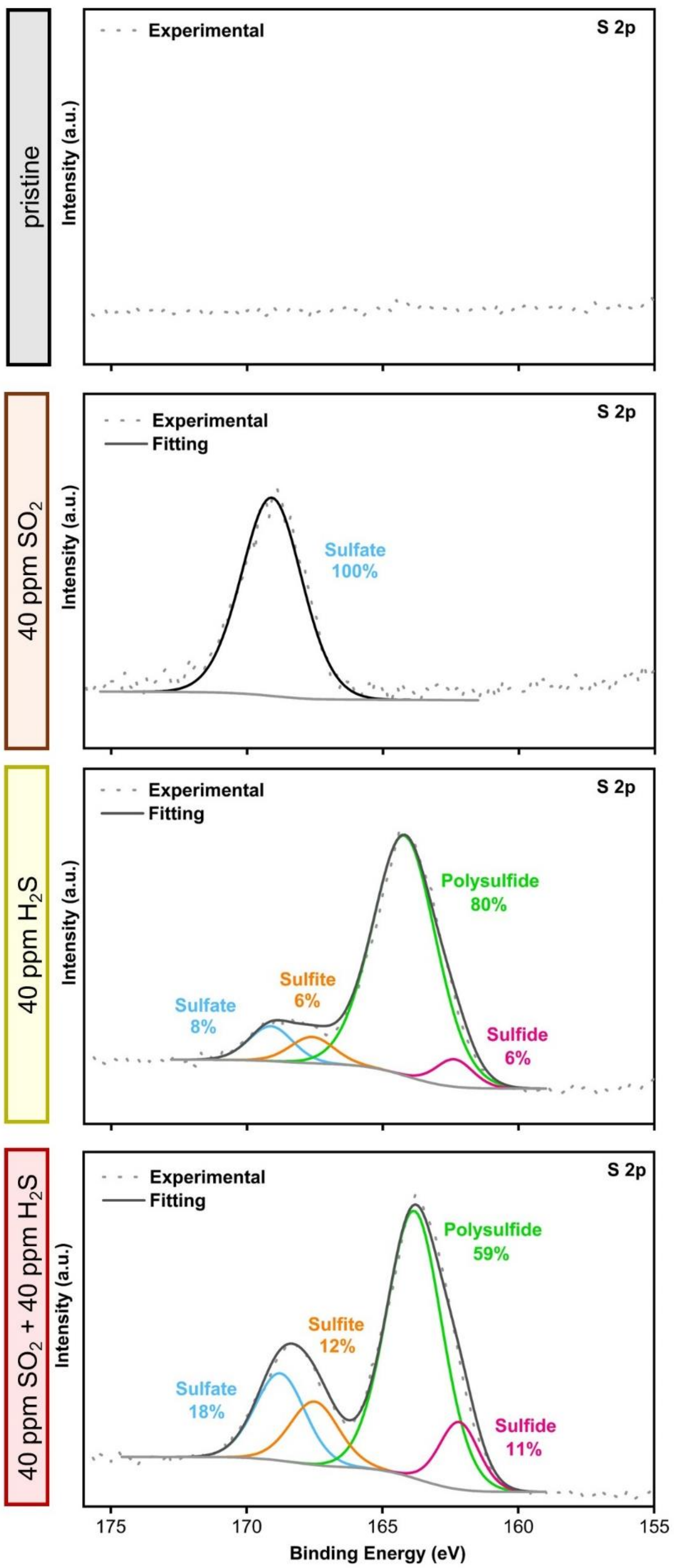

Figure S63. High-resolution ex situ XPS spectra in the S 2p region comparing pristine $Cu_3(HHTP)_2$, and material exposed to 40 ppm SO_2, 40 ppm H_2S, and dual exposure of both 40 ppm SO_2 and 40 ppm H_2S.

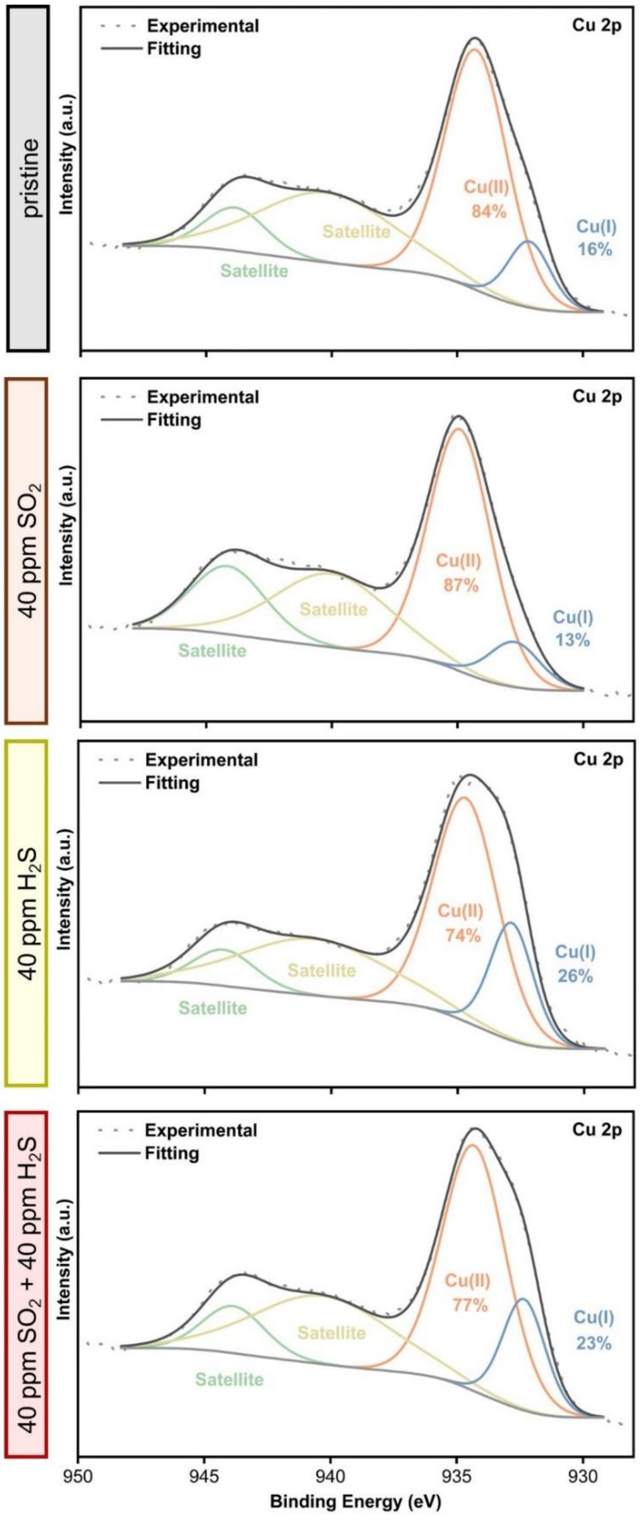

Figure S64. High-resolution ex situ XPS spectra in the Cu 2p region comparing pristine $Cu_3(HHTP)_2$, and material exposed to 40 ppm SO_2, 40 ppm H_2S, and dual exposure of both 40 ppm SO_2 and 40 ppm H_2S.

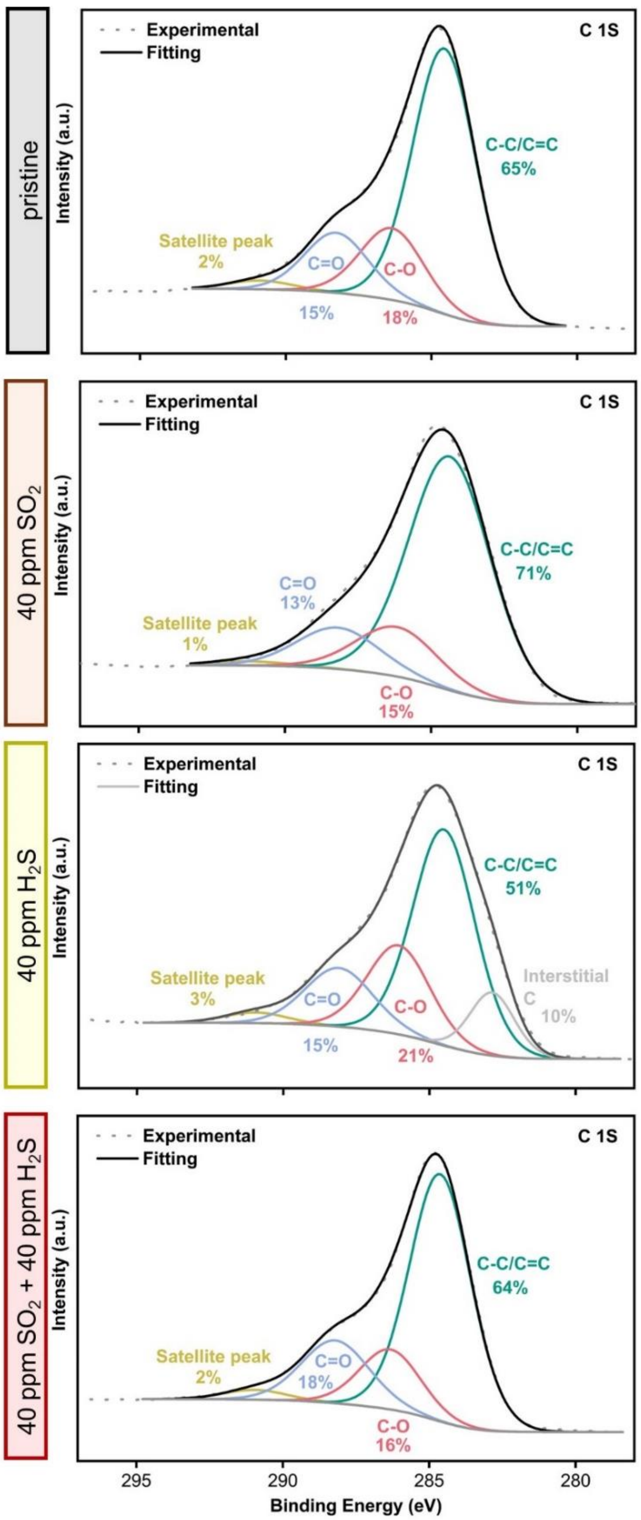

Figure S65. High-resolution ex situ XPS spectra in the C 1S region comparing pristine $Cu_3(HHTP)_2$, and material exposed to 40 ppm SO_2, 40 ppm H_2S, and dual exposure of both 40 ppm SO_2 and 40 ppm H_2S.

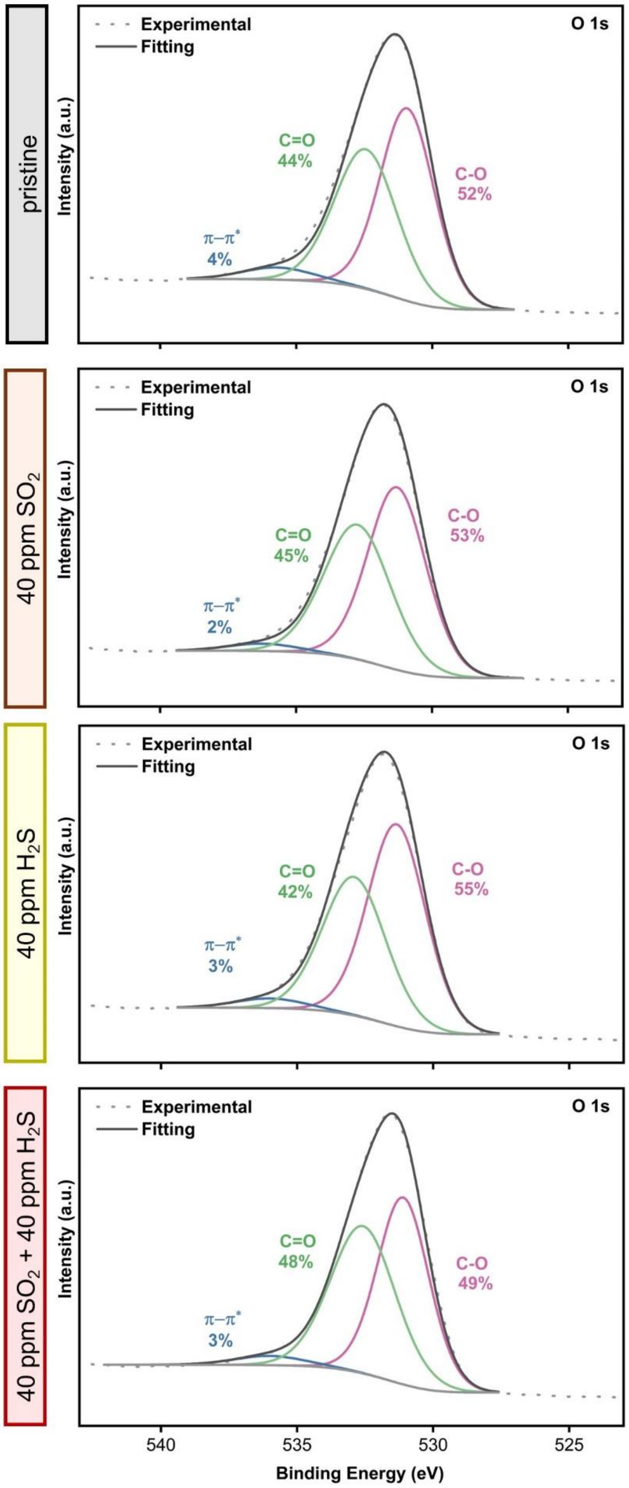

Figure S66. High-resolution ex situ XPS spectra in the O 1S region comparing pristine $Cu_3(HHTP)_2$, and material exposed to 40 ppm SO_2, 40 ppm H_2S, and dual exposure of both 40 ppm SO_2 and 40 ppm H_2S.

XIV.i. XPS of $Zn_3(HHTP)_2$ exposed to SO_2, H_2S, and a mixture of SO_2 & H_2S

Upon exposure to individual exposure to SO_2 and H_2S, XPS revealed SO_4^{2-}:SO_3^{2-}:S_x:S^{2-} ratios of 58:15:14:11, and 10:10:56:24, respectively (**Figure S67**). When exposed to a mixture of SO_2 and H_2S, the Zn-based MOF retained a ratio of 11:13:53:23 (**Figure S67**). Similarly with the Ni-based MOF, following exposure to all gases, $Zn_3(HHTP)_2$ did not exhibit a change in metal center oxidation state. There were minimal changes in the peak positions in the Zn 2p region, which has previously been attributed to changes in bound surface ligands coordinating to the metal center (**Figure S68**).[3] We hypothesize that the reduction to the MOF occurs at the HHTP ligand as evidenced by an increase in C singly bound to O as compared to C=O content (**Figure S69**).

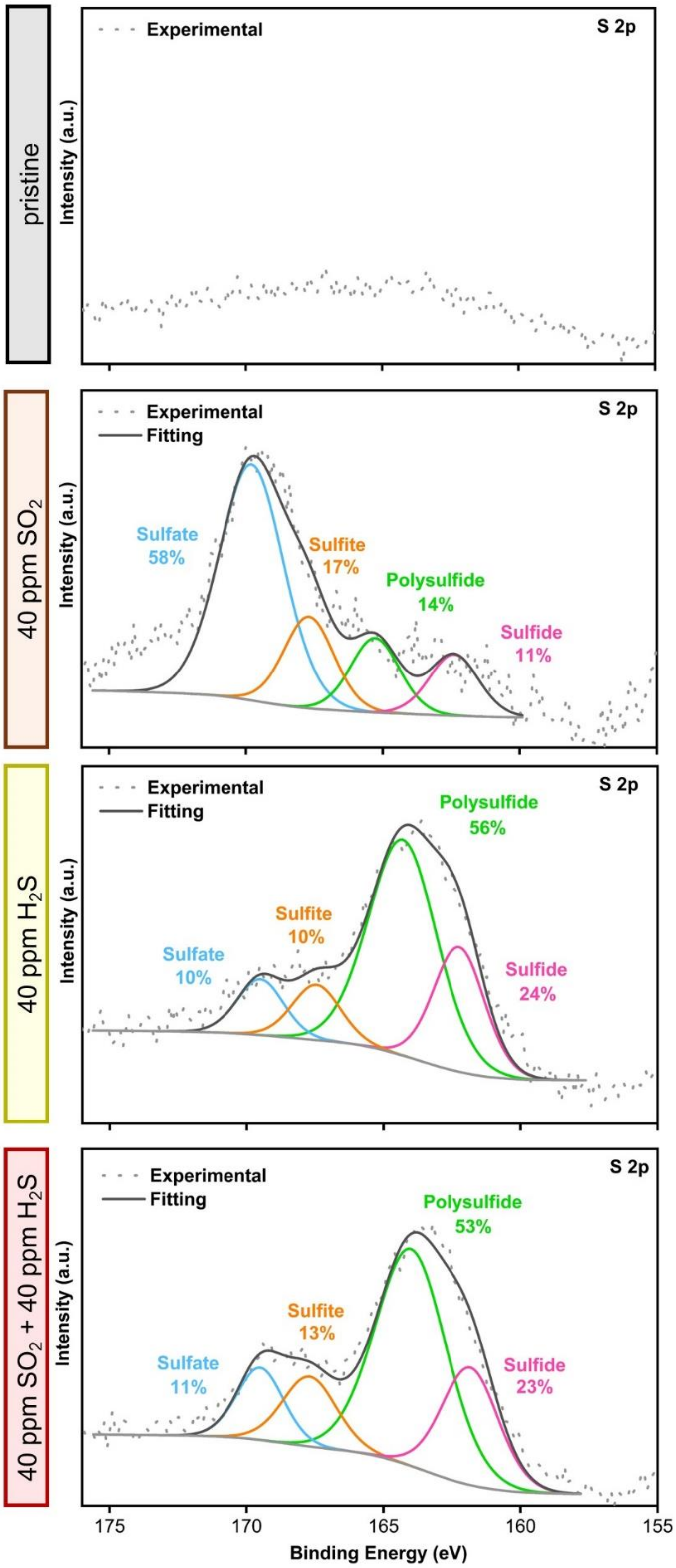

Figure S67. High-resolution ex situ XPS spectra in the S 2p region comparing pristine $Zn_3(HHTP)_2$, and material exposed to 40 ppm SO_2, 40 ppm H_2S, and dual exposure of both 40 ppm SO_2 and 40 ppm H_2S.

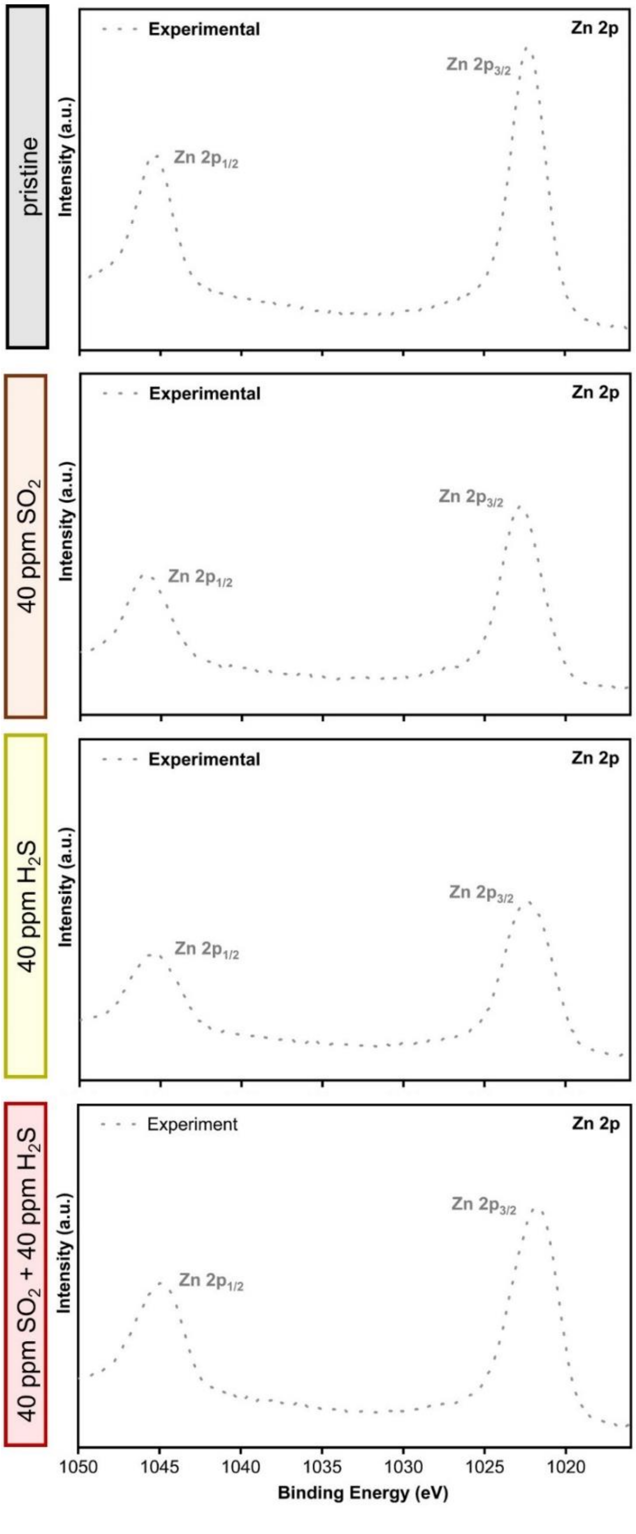

Figure S68. High-resolution ex situ XPS spectra in the Zn 2p region comparing pristine $Zn_3(HHTP)_2$, and material exposed to 40 ppm SO_2, 40 ppm H_2S, and dual exposure of both 40 ppm SO_2 and 40 ppm H_2S.

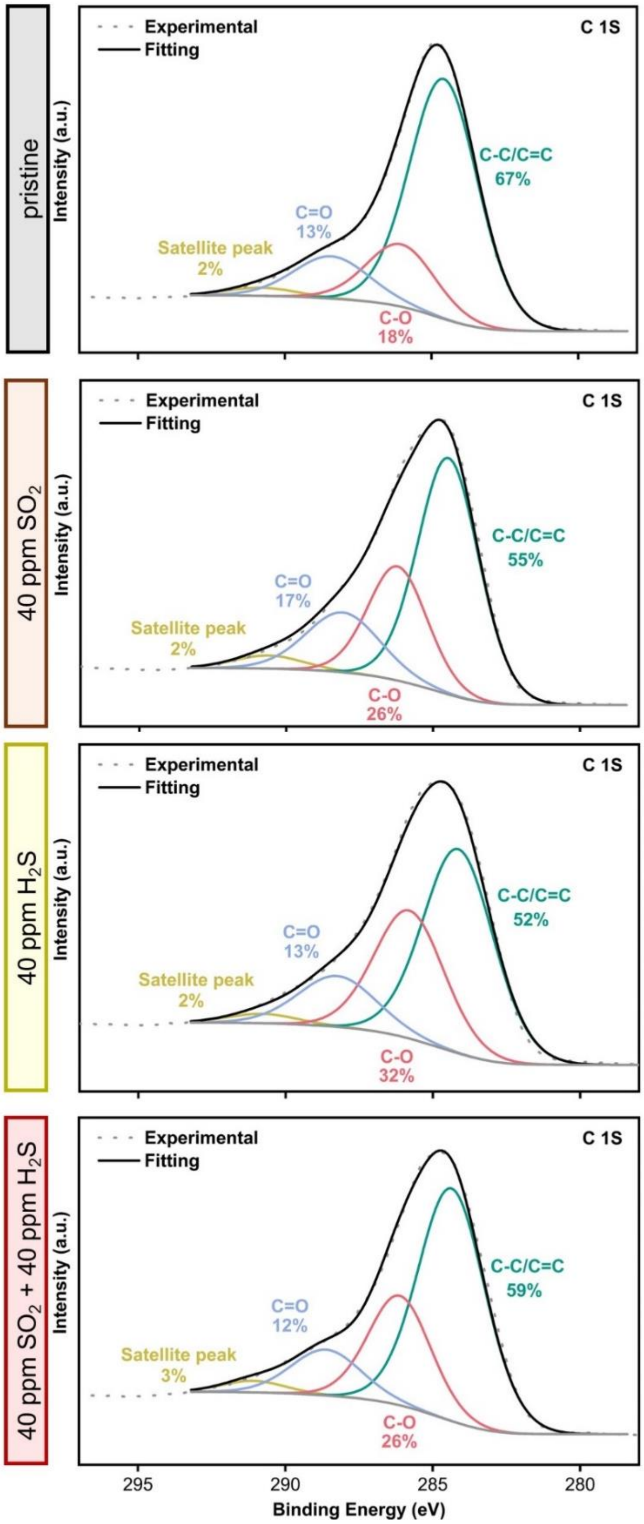

Figure S69. High-resolution ex situ XPS spectra in the C 1S region comparing pristine $Zn_3(HHTP)_2$, and material exposed to 40 ppm SO_2, 40 ppm H_2S, and dual exposure of both 40 ppm SO_2 and 40 ppm H_2S.

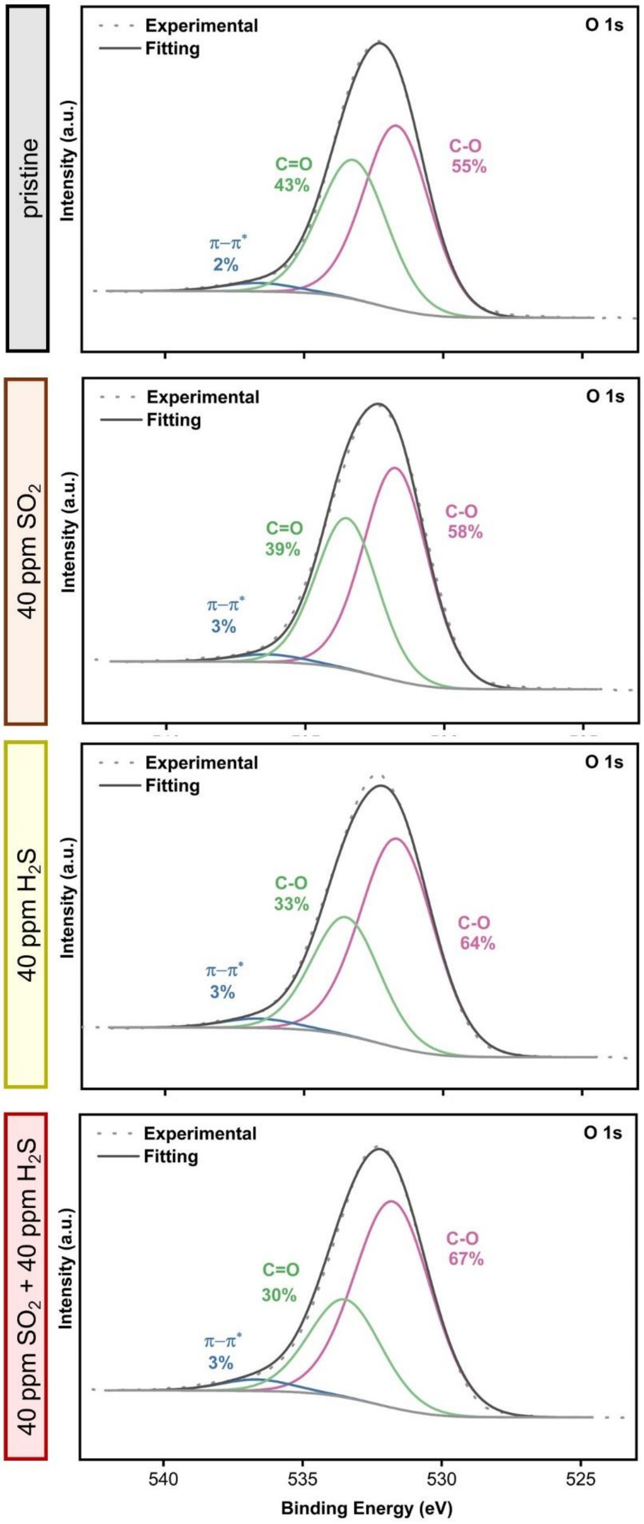

Figure S70. High-resolution ex situ XPS spectra in the O 1S region comparing pristine $Zn_3(HHTP)_2$, and material exposed to 40 ppm SO_2, 40 ppm H_2S, and dual exposure of both 40 ppm SO_2 and 40 ppm H_2S.

XIV.j. DRIFTS of $M_3(HHTP)_2$ exposed to NH_3

Upon exposure to 1% NH_3 in dry N_2 followed by 20 minutes of N_2 purging, $M_3(HHTP)_2$ (M=Ni, Cu, and Zn) exhibited differing spectral features (**Figure S71**). Ni- and $Zn_3(HHTP)_2$ materials exhibited a negative-going peak at ~3400 cm-1 indicating the removal of water upon exposure to NH_3. The notable absence of the negative-going peak at ~3400 cm^{-1} for the $Cu_3(HHTP)_2$ suggests that the Cu-based MOF interacts directly with the NH_3 gas in a typical manner of a p-type semiconductor interacting with a reducing gas. The increase in conductance following NH_3 exposure for Ni- and $Zn_3(HHTP)_2$ materials may result from a dehydration mechanism and subsequent ligand reorganization.

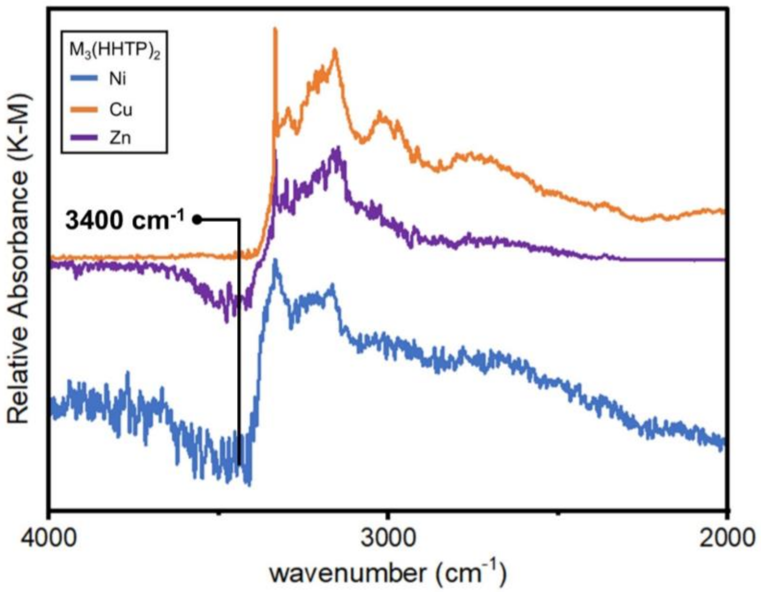

Figure S71. Comparative time resolved DRIFTS difference spectra of $Ni_3(HHTP)_2$ (blue), $Cu_3(HHTP)_2$ (orange), and $Zn_3(HHTP)_2$ (purple) exposed to 1% NH_3 in dry N_2 for 20 minutes followed by purging in dry N_2 for 20 minutes. Note: The y-axis shows absorbance intensity without normalization, allowing for direct comparisons of spectral intensities across the three MOFS.

XV. PXRD analysis of MOFs post-analyte exposure

To assess the crystallinity of the MOFs following exposure to either SO_2 or H_2S, the powder samples were subjected to either 40 ppm or 10,000 ppm of each analyte. MOF powder was placed in a vial and subjected to a flow of either 40 ppm or 10,000 ppm of analyte. 40 ppm analyte flow was achieved by varying the flowrates of the mass flow controller set up described in **Section XI** of the Supporting information. 10,000 ppm analyte flow was achieved by flowing the contents of a gas tank (1% analyte in a balance of N_2) to the powder at a flow rate of 4 mL/min. For each exposure, the powder was subjected to analyte flow for 20 minutes, then immediately analyzed via PXRD.

There was minimal structural degradation to the $M_3(HHTP)_2$ (M= Ni, Cu, Zn) upon exposure to SO_2 at 40 and 10,000 ppm concentrations. For all three MOFs exposed SO_2, there were decreases in peak intensity indicating slight material degradation. Upon exposure to H_2S at 40 and 10,000 ppm concentrations $M_3(HHTP)_2$ (M= Ni, Cu, Zn) exhibited slight decrease in peak intensity. However, upon exposure of 10,0000 ppm H_2S to $Cu_3(HHTP)_2$, MOF crystallinity was dramatically reduced due to strong interactions with the analyte as evidence by the strong decrease in peak intensity and shifting of peak position (**Figure S75b**).

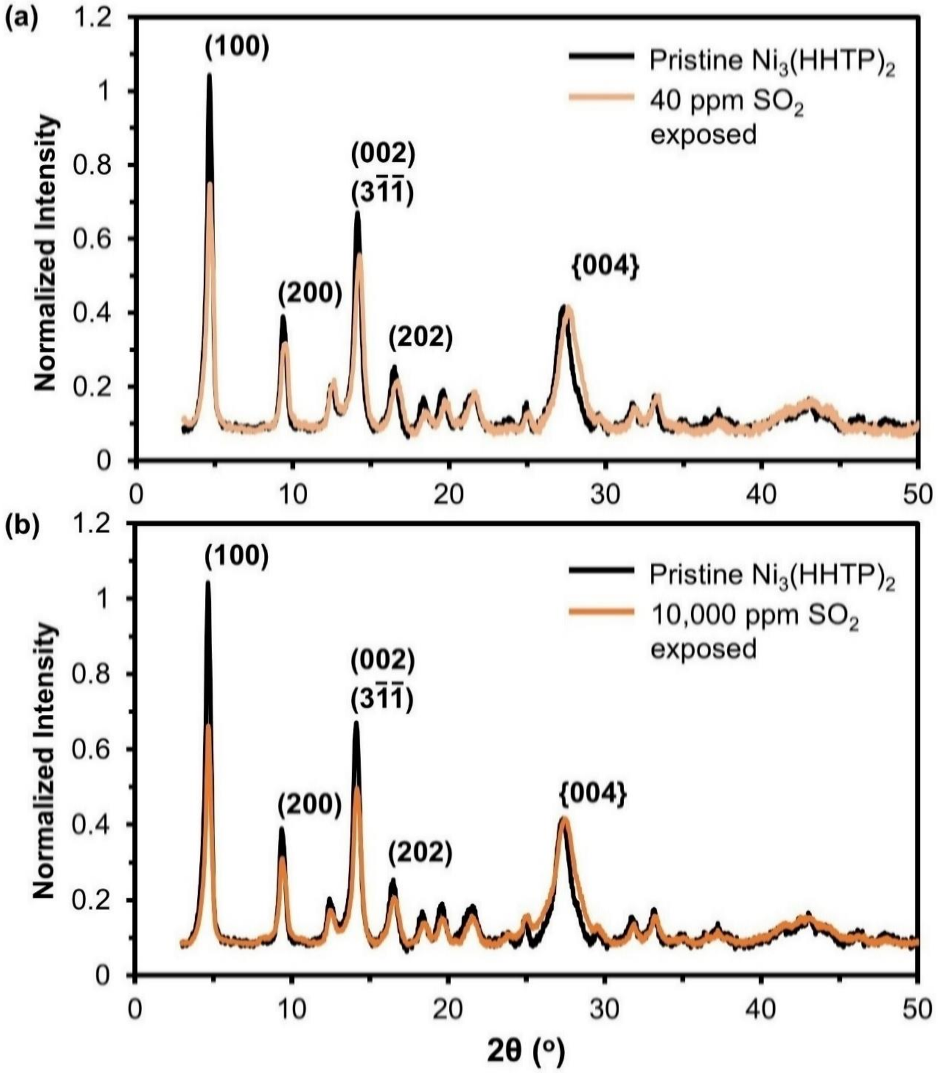

Figure S72. PXRD analysis of $Ni_3(HHTP)_2$ powder before and after exposure to (**a**) 40 ppm SO_2 and (**b**) 10,000 ppm SO_2.

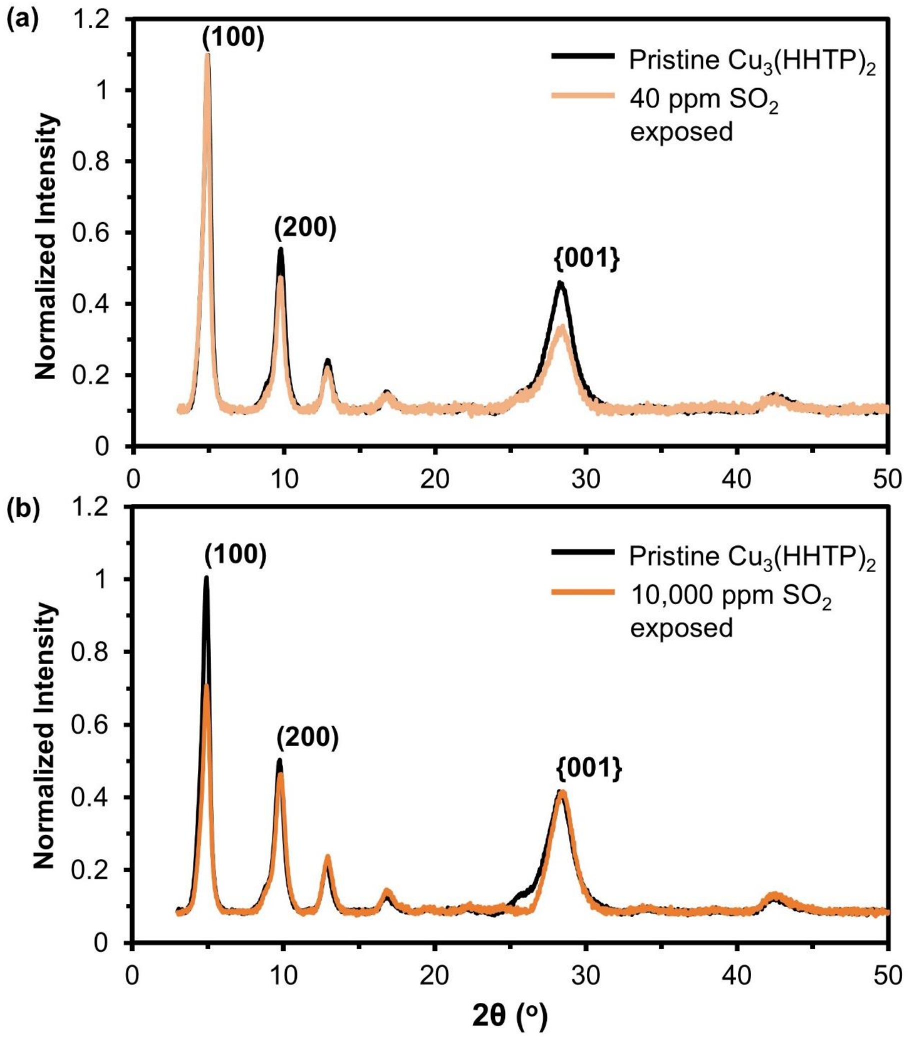

Figure S73. PXRD analysis of $Cu_3(HHTP)_2$ powder before and after exposure to (**a**) 40 ppm SO_2 and (**b**) 10,000 ppm SO_2.

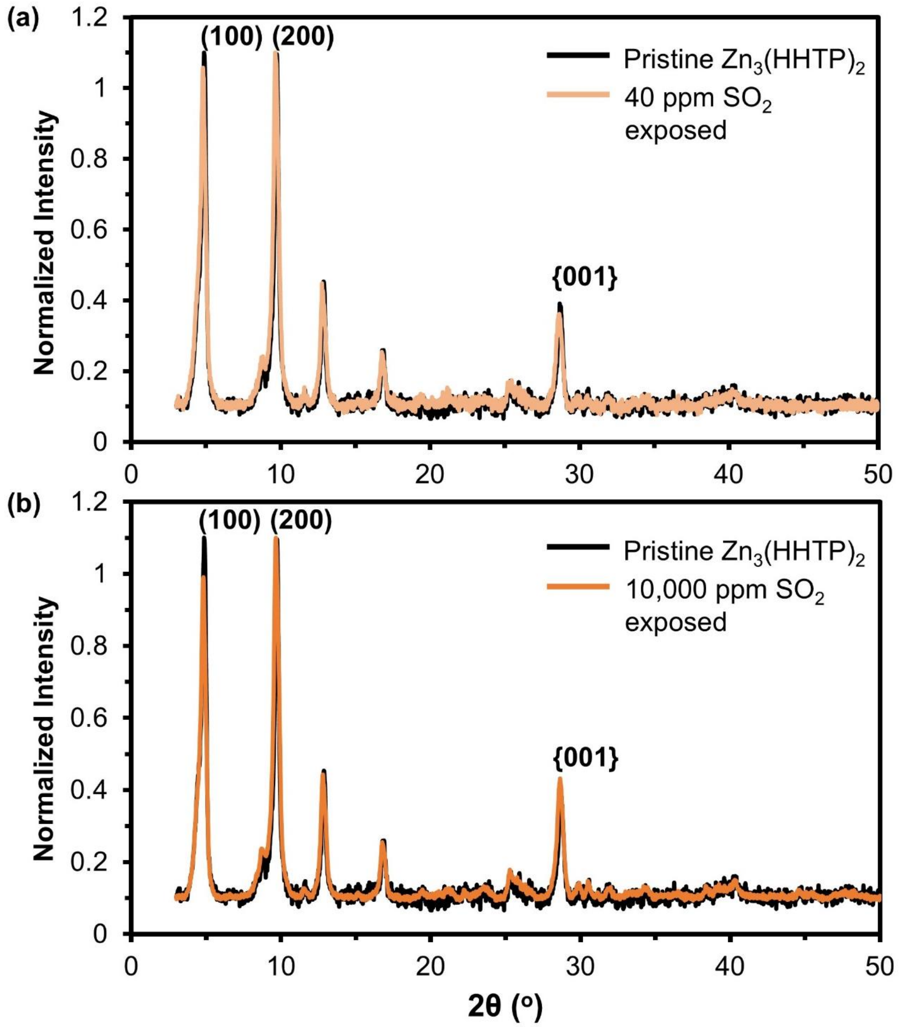

Figure S74. PXRD analysis of $Zn_3(HHTP)_2$ powder before and after exposure to (**a**) 40 ppm SO_2 and (**b**) 10,000 ppm SO_2.

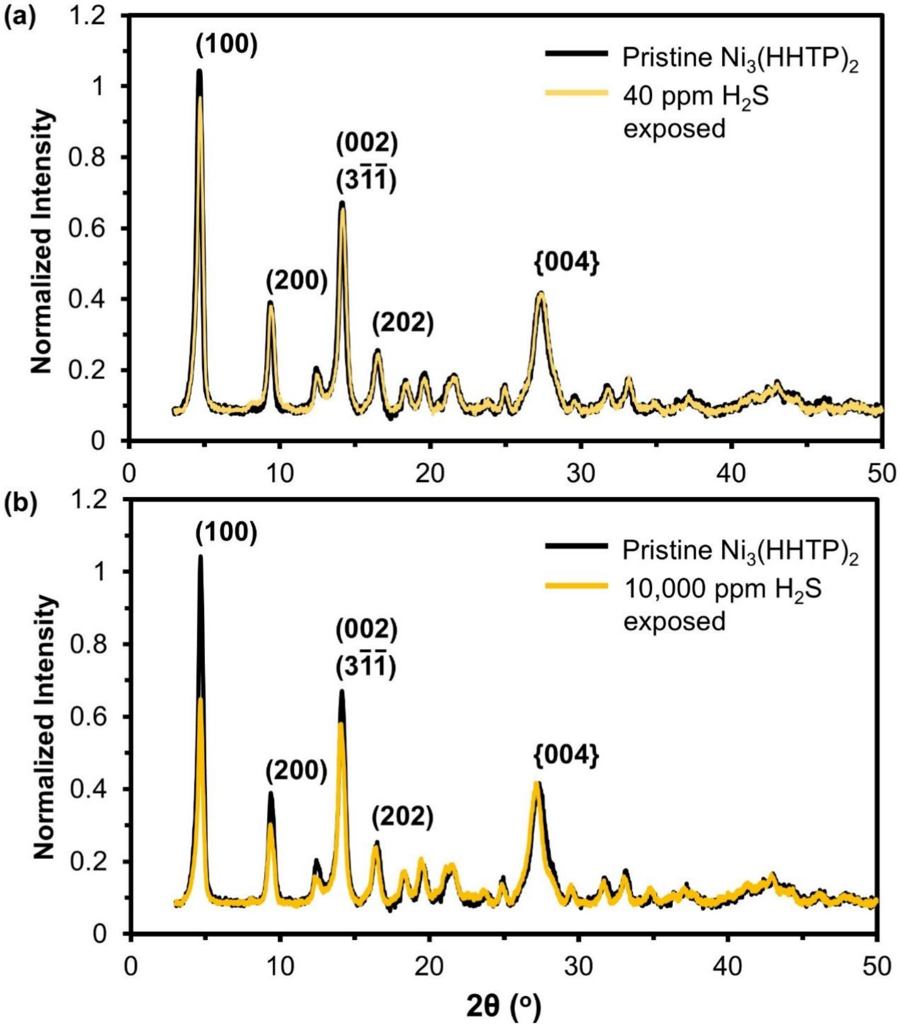

Figure S75. PXRD analysis of $Ni_3(HHTP)_2$ powder before and after exposure to (**a**) 40 ppm H_2S and (**b**) 10,000 ppm H_2S.

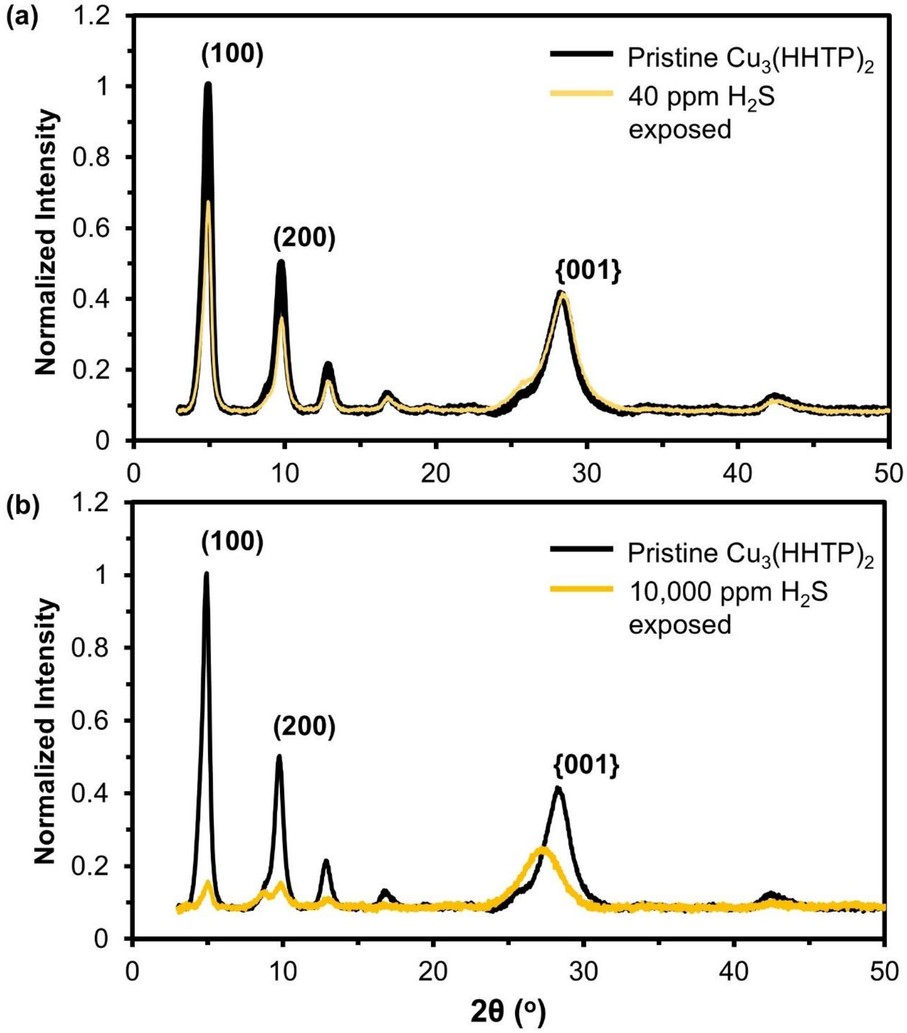

Figure S76. PXRD analysis of $Cu_3(HHTP)_2$ powder before and after exposure to (**a**) 40 ppm H_2S and (**b**) 10,000 ppm H_2S.

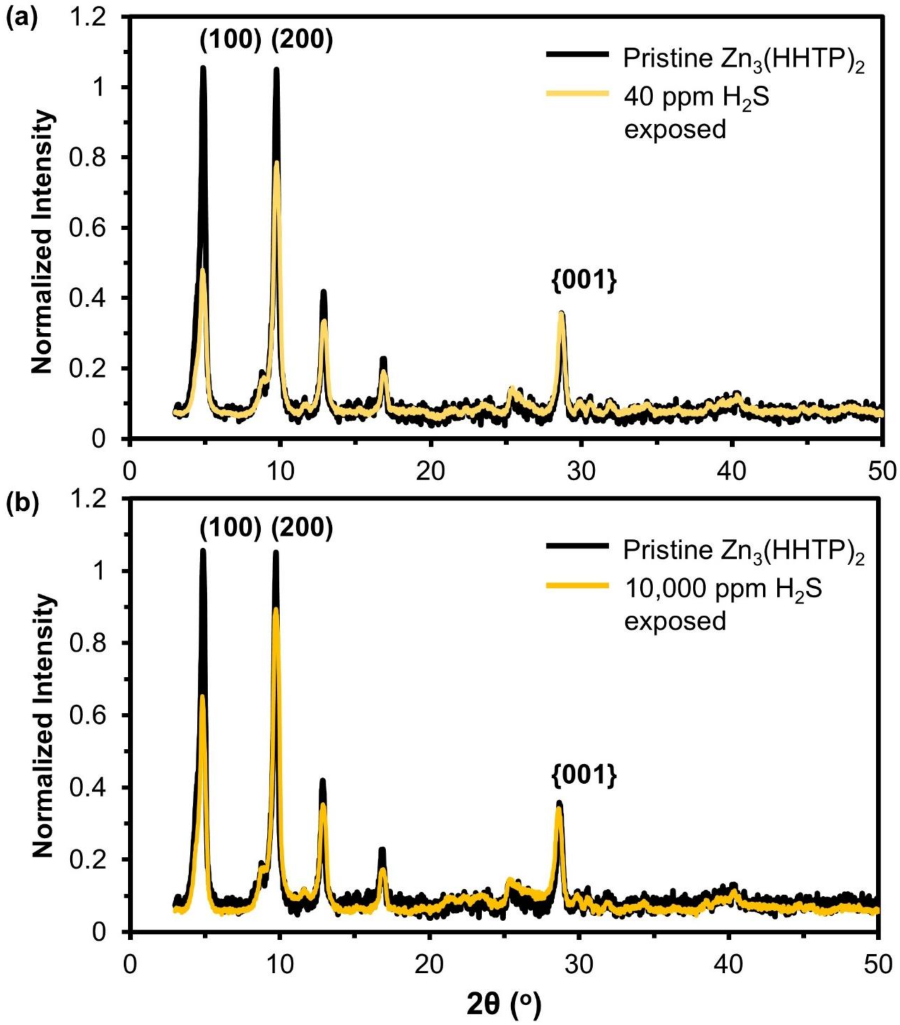

Figure S77. PXRD analysis of $Zn_3(HHTP)_2$ powder before and after exposure to (**a**) 40 ppm H_2S and (**b**) 10,000 ppm H_2S.

XVI. References

(1) Hmadeh, M.; Lu, Z.; Liu, Z.; Gándara, F.; Furukawa, H.; Wan, S.; Augustyn, V.; Chang, R.; Liao, L.; Zhou, F.; et al. New Porous Crystals of Extended Metal-Catecholates. *Chem. Mater.* **2012**, *24* (18), 3511-3513. DOI: 10.1021/cm301194a.

(2) Gittins, J. W.; Balhatchet, C. J.; Chen, Y.; Liu, C.; Madden, D. G.; Britto, S.; Golomb, M. J.; Walsh, A.; Fairen-Jimenez, D.; Dutton, S. E.; et al. Insights into the electric double-layer capacitance of two-dimensional electrically conductive metal–organic frameworks. *J. Mater. Chem. A.* **2021**, *9* (29), 16006-16015. DOI: 10.1039/D1TA04026J.

(3) Choi, J. Y.; Stodolka, M.; Kim, N.; Pham, H. T. B.; Check, B.; Park, J. 2D conjugated metal-organic framework as a proton-electron dual conductor. *Chem.* **2023**, *9* (1), 143-153. DOI: 10.1016/j.chempr.2022.09.016.

(4) Smith, M. K.; Mirica, K. A. Self-Organized Frameworks on Textiles (SOFT): Conductive Fabrics for Simultaneous Sensing, Capture, and Filtration of Gases. *J. Am. Chem. Soc.* **2017**, *139* (46), 16759-16767. DOI: 10.1021/jacs.7b08840.

(5) Ko, M.; Aykanat, A.; Smith, M. K.; Mirica, K. A. Drawing Sensors with Ball-Milled Blends of Metal-Organic Frameworks and Graphite. *Sensors (Basel)* **2017**, *17* (10). DOI: 10.3390/s17102192.

(6) Eagleton, A. M.; Ko, M.; Stolz, R. M.; Vereshchuk, N.; Meng, Z.; Mendecki, L.; Levenson, A. M.; Huang, C.; MacVeagh, K. C.; Mahdavi-Shakib, A.; et al. Fabrication of Multifunctional Electronic Textiles Using Oxidative Restructuring of Copper into a Cu-Based Metal-Organic Framework. *J. Am. Chem. Soc.* **2022**, *144* (51), 23297-23312. DOI: 10.1021/jacs.2c05510.

(7) Fantauzzi, M.; Elsener, B.; Atzei, D.; Rigoldi, A.; Rossi, A. Exploiting XPS for the identification of sulfides and polysulfides. *RSC Adv.* **2015**, *5* (93), 75953-75963. DOI: 10.1039/c5ra14915k.

(8) Luo, B.; Liu, Q.; Deng, J.; Li, S.; Yu, L.; Lai, H. Determining the lead-sulfur species formed on smithsonite surfaces during lead-ion enhanced sulfidation processing. *Appl. Surf. Sci.* **2020**, *506*. DOI: 10.1016/j.apsusc.2019.144628.

(9) Meng, Z.; Stolz, R. M.; De Moraes, L. S.; Jones, C. G.; Eagleton, A. M.; Nelson, H. M.; Mirica, K. A. Gas-Induced Electrical and Magnetic Modulation of Two-Dimensional Conductive Metal-Organic Framework. *Angew. Chem. Int. Ed. Engl.* **2024**, *63* (24), e202404290. DOI: 10.1002/anie.202404290.

(10) Lacey, M. J.; Yalamanchili, A.; Maibach, J.; Tengstedt, C.; Edström, K.; Brandell, D. The Li–S battery: an investigation of redox shuttle and self-discharge behaviour with $LiNO_3$-containing electrolytes. *RSC Adv.* **2016**, *6* (5), 3632-3641. DOI: 10.1039/c5ra23635e.

(11) Chang, C. Infrared studies of SO_2 on γ-alumina. *J. Catal.* **1978**, *53* (3), 374-385. DOI: 10.1016/0021-9517(78)90109-4.

(12) Siriwardane, R. V.; Woodruff, S. In Situ Fourier Transform Infrared Characterization of Sulfur Species Resulting from the Reaction of Water Vapor and Oxygen with Zinc Sulfide. *Ind. Eng. Chem. Res.* **1997**, *36* (12), 5277-5281. DOI: 10.1021/ie970343e.

(13) Nakamoto, K. *Infrared and Raman Spectra of Inorganic and Coordination Compounds*; 2008. DOI: 10.1002/9780470405888.

(14) Lane, M. D. Mid-infrared emission spectroscopy of sulfate and sulfate-bearing minerals. *Am. Mineral.* **2007**, *92* (1), 1-18. DOI: 10.2138/am.2007.2170.

(15) Savage, M.; Cheng, Y.; Easun, T. L.; Eyley, J. E.; Argent, S. P.; Warren, M. R.; Lewis, W.; Murray, C.; Tang, C. C.; Frogley, M. D.; et al. Selective Adsorption of Sulfur Dioxide in a Robust Metal-Organic Framework Material. *Adv. Mater.* **2016**, *28* (39), 8705-8711. DOI: 10.1002/adma.201602338.

(16) Elder, A. C.; Bhattacharyya, S.; Nair, S.; Orlando, T. M. Reactive Adsorption of Humid SO_2 on Metal–Organic Framework Nanosheets. *J. Phys. Chem. C* **2018**, *122* (19), 10413-10422. DOI: 10.1021/acs.jpcc.8b00999.

(17) Hadjiivanov, K. I.; Panayotov, D. A.; Mihaylov, M. Y.; Ivanova, E. Z.; Chakarova, K. K.; Andonova, S. M.; Drenchev, N. L. Power of Infrared and Raman Spectroscopies to Characterize

Metal-Organic Frameworks and Investigate Their Interaction with Guest Molecules. *Chem. Rev.* **2021**, *121* (3), 1286-1424. DOI: 10.1021/acs.chemrev.0c00487.
